# Transcription factor PBX4 regulates limb development and haematopoiesis in mice

**DOI:** 10.1111/cpr.13580

**Published:** 2024-01-17

**Authors:** Yan Ning, Shuguang Duo, Xiwen Lin, Hongbo Zhang, Jifeng Fei, Bao Zhang, Yanyun Zeng, Dan Xie, Jian Chen, Xiaowei Liu, Chunsheng Han

**Affiliations:** ^1^ State Key Laboratory of Stem Cell and Reproductive Biology, Institute of Zoology Chinese Academy of Sciences Beijing China; ^2^ Institute for Stem Cell and Regeneration Chinese Academy of Sciences Beijing China; ^3^ Beijing Institute for Stem Cell and Regenerative Medicine Beijing China; ^4^ Savaid Medical School University of Chinese Academy of Sciences Beijing China; ^5^ The Key Laboratory for Stem Cells and Tissue Engineering, Ministry of Education, Zhongshan School of Medicine Sun Yat‐sen University Guangzhou China; ^6^ Department of Pathology, Guangdong Provincial People's Hospital Guangdong Academy of Medical Sciences Guangzhou China; ^7^ Medical College of Jiaying University Meizhou China

## Abstract

The mammalian Pre‐B cell leukaemia transcription factors 1–4 (PBX1‐4) constitutes the PBC class of the homeodomain (HD)‐containing proteins, which play important roles in diverse developmental processes. The functions and the underlying molecular mechanisms of PBX1‐3 but not PBX4 have been extensively studied, and they have been reported to direct essential morphogenetic processes and organogenesis. In the present study, we generated knockin mice of FLAG‐tagged PBX4 and the Pbx4 knockout (KO) mice and carried out in‐depth characterisation of PBX4 expression and function. PBX4 was initially detected only in the testis among several organs of the adult mice and was expressed in spermatocytes and spermatids. However, no abnormality in spermatogenesis, but growth retardation and premature death after birth were observed in most adult Pbx4 KO mice. These animals were inactive and had shorter hindlimbs and lower numbers of reticulocytes and lymphocytes, probably caused by abnormalities at earlier developmental stages. Pbx4 mRNAs were indeed detected in several embryonic cell types related to limb development by in situ hybridisation and single‐cell RNA‐sequencing analysis. Pbx4 protein was also detected in the bone marrow of adult mice with a lower level compared with that in the testis. PBX4 preferentially binds to the promoters of a large number of genes including those for other HD‐containing proteins and ribosomal proteins whose mutations are related to anaemia. PBX4‐binding sites are enriched in motifs similar to those of other HD‐containing proteins such as PKNOX1 indicating that PBX4 may also act as a co‐transcription factor like other PBC proteins. Together, these results show that PBX4 participates in limb development and haematopoiesis while its function in spermatogenesis has not been revealed by gene KO probably due to the complementary effects of other genes.

## Graphical abstract

PBX1‐4 constitute the PBC class of the homeodomain‐containing proteins. Ning et al. generated *Pbx4* knockout mice and carried out in‐depth phenotypic analysis and found that PBX4 plays an important role in hindlimb development and haematopoiesis, consistent with the functions of the other PBC members.

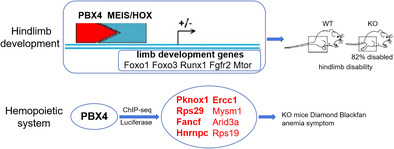

## 1 INTRODUCTION

The homeodomain (HD)‐containing Hox proteins are transcription factors and the major architects of the body plan during development throughout the animal kingdom.^1^ They are defined by a highly conserved DNA‐binding HD that binds to a TAAT core sequence, which occurs approximately once every 500 base pair within the genome.^2^ However, different Hox proteins are able to trigger very specific developmental programs. To explain this apparent paradox, it was proposed that the specificities of Hox proteins are achieved by their partnerships with cofactors.

PBX1‐4 constitute the mammalian PBC family of the HD‐containing proteins, and their non‐mammalian homologues have been identified in species as low as worms.^3^ PBX1 was initially identified as the product of a proto‐oncogene in human leukaemia induced by the expression of the fusion protein E2a‐PBX1.^4^, ^5^ PBX2‐4 were discovered based on sequence similarity searches, and they bear extensive homology to PBX1.^6^, ^7^ The *Drosophila* homologue of PBC proteins, the extradenticle (exd) gene product, was first identified as a cofactor of Hox proteins during development.^8^, ^9^ Although more and more Hox‐PBC partnerships have been identified, PBC proteins also interact with diverse non‐Hox proteins including signal transducers, nuclear receptors and chromatin remodelers.^3^

In mice, *Pbx1‐3* are widely expressed in diverse embryonic and adult tissues/organs with both overlapping and unique distributions. *Pbx1* knockout (KO) mice die at embryonic day (E) 15.5 with severe hypoplasia, ectopia, or aplasia in multiple organs as well as widespread defects of the axial and appendicular skeleton.^10^ *Pbx2* KO mice are viable and display no obvious phenotype.^11^ However, *Pbx1* ^−/−^/*Pbx2* ^−/−^ embryos lack hindlimbs altogether while *Pbx1* ^−/−^/*Pbx2* ^+/−^ embryos display loss of distal hindlimb elements, suggesting a quantitative model in which the threshold of PBC proteins is crucial for limb development.^12^ *Pbx3* KO mice die of central respiratory failure due to abnormal activity of inspiratory neurons in the medulla within several hours after birth.^13^

*Pbx4* was previous reported to be exclusively expressed in mouse testis by Northern blotting analysis while very low‐level expression was also detected in other organs such as brain, spleen, ovary, placenta and heart.^7^ Its mRNAs were also detected in the limb buds of mouse embryos by in situ hybridisation.^14^ In the testis, its mRNA and protein were specifically detected in pachytene spermatocytes, and the protein was highly enriched in the XY chromosomal bivalent, a hallmark of pachytene spermatocytes. In that study, multiple low level alternatively spliced variants of *Pbx4* were also detected in several organs including the testis. Currently, there has been no report for the phenotypic evaluation of *Pbx4* KO mice. Interestingly, *Pbx4* in zebrafish is expressed broadly during early‐stage embryonic development and the abolishment of its protein product due to a nonsense mutation resulted in disrupted segmental patterning in the hindbrain and anterior trunk, and the embryos die of multiple organ defects.^15^

In the present study, we set out to investigate the expression of *Pbx4* in spermatogenic cells. We detected and cloned two cDNAs predicted to encode the full‐length PBX4 and an N‐terminal truncated isoform. We produced knockin mice, in which a 3 × FLAG epitope is fused to the C‐end shared by the two protein isoforms, but only detected the full‐length protein exclusively in the testis. Gene KO mice aiming to abolish either the full‐length protein (KO) or both isoforms (KO‐2) were generated, and the spermatogenesis in both KO and KO‐2 mice was found to be normal. However, we observed growth retardation, hindlimb disability, premature death and anaemia of the KO mice. The expression of *Pbx4* in limb bud cells was confirmed by in situ hybridisation and single‐cell RNA‐sequencing analysis. We carried out ChIP‐seq analysis of the PBX4 target genes and found that genes involved in limb development and haematopoiesis were putative targets of PBX4. For the first time, these results indicate that PBX4 executes an important role in limb development and haematopoiesis by regulating the expression of a large number of genes.

## 2 RESULTS

### 2.1 Mouse *Pbx4* mRNAs are alternatively spliced and its protein is expressed in meiotic and postmeiotic spermatogenic cells

**FIGURE 1:**
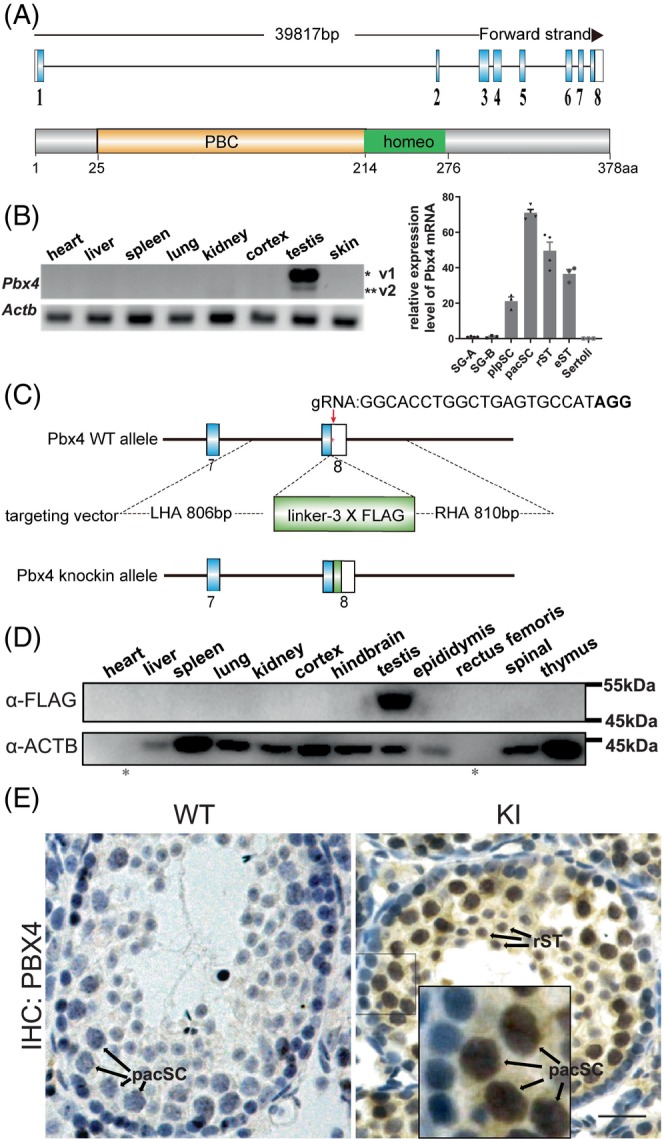
PBX4 is specifically expressed in mouse testis. (A) Schematic diagrams of *Pbx4* gene structure and PBX4 protein domains. Blue and white rectangles indicate protein‐coded exons and UTR regions, respectively. (B) Left panel: detection of *Pbx4* expression by RT‐PCRs in various tissues of adult mice. Right panel: quantitative RT‐PCR results of *Pbx4* expression in different testicular cells. eST, elongating spermatids; pacSC, pachytene spermatocytes; plpSC, preleptotene spermatocytes; rST, round spermatids; Sertoli, Sertoli cells; SG‐A, type A spermatogonia; SG‐B, type B spermatogonia. The expression levels are normalised by that of SG‐A, *n* ≥ 3. (C) Schematic diagram of PBX4‐FLAG knockin (KI) strategy. LHA: left homologous arm; RHA: right homologous arm. (D) Western blot analyses of PBX4 in multiple mouse organs using α‐FLAG. * labels the absence of ACTB signal in heart and rectus femoris samples due to low expression levels. Equal amounts of total protein in all samples were loaded based on quantification using the BCA kit. (E) Immunohistochemical (IHC) staining of PBX4‐FLAG in testicular sections using α‐FLAG. pacSC, pachytene spermatocytes; rST, round spermatids. Scale bar, 20 μm.

PBX4 proteins contain a 63‐residue HD typical of all Three Amino acids Loop Extension class of HD‐containing proteins and a PBC domain that is characteristic of the PBC subclass (Figure 1A, Figure S1). The human and mouse *Pbx4* genes are predicated to generate multiple alternatively spliced variants based on their annotations in the NCBI gene database. We carried out nested RT‐PCR analysis on mouse testis RNAs to identify *Pbx4* transcripts. We sequenced the cloned cDNAs and identified a total of 12 alternatively spliced variants (Figure S2A). Variant 1 encodes the full‐length PBX4 protein, which has 378 amino acids, and is predicted to have a molecular mass of 43 kDa. We amplified the sequences between exons 2 and 6 by RT‐PCR and found that only cDNAs of variant 1 and 2 could be visualised in the stained gel after electrophoresis and that variant 1 was much more abundant than variant 2 (Figure 1B). According to the mouse ENCODE transcriptome data, *Pbx4* is expressed in multiple organs such as the testis, the brain, the thymus and the spleen, and the expression level in the testis is more than 10‐fold higher than in any of the other organs (BioProject: PRJNA66167). Our RT‐PCR results showed that both variant 1 and 2 were only detected in the adult testis but not in other adult organs suggesting that the expression of *Pbx4* in any other organ, if any, is too low to be detected by RT‐PCR. We isolated different testicular cells including type A spermatogonia, type B spermatogonia, preleptotene spermatocytes, pachytene spermatocytes, round spermatids, elongating spermatids and Sertoli cells and examined *Pbx4* expression in these cells and found that it was high in different types of spermatocytes and spermatids c (Figure 1B, Figure S2B).

The cDNA of full‐length PBX4 was cloned into a eukaryotic expressing vector, by which a FLAG‐tagged protein (FLAG‐PBX4) can be expressed. After the plasmid was transfected into the 293FT cells, the protein was detected by the anti‐FLAG antibody (α‐FLAG) to have a molecular mass of about 50 kDa, slightly bigger than expected (Figure S3A). We next generated a knockin mouse line, in which a 3× FLAG epitope was fused to the C‐terminus of PBX4 (Figure 1C, Figure S3B,C), to investigate the expression of PBX4 because we found that a home‐made antibody against PBX4 (α‐PBX4) is only suitable for Western blotting assays but not for immunostaining on tissue sections (Figure S3D,E). We conducted Western blotting analysis of PBX4‐FLAG expression in multiple adult organs by using α‐FLAG and found that only the full‐length PBX4 was exclusively expressed in the testis, consistent with the RT‐PCR result (Figure 1D). We also performed immunostaining of the PBX4‐FLAG on the testis sections and found that PBX4 was present in spermatocytes and spermatids but not in spermatogonia (Figure 1E). The immunostaining signal in spermatogenic cells using α‐FLAG was evenly distributed in the nucleus in sharp contrast to the non‐specific signal enriched in acrosomes of spermatid using α‐PBX4 (Figure S3E).

### 2.2 Spermatogenesis is not disrupted in *Pbx4* knockout mice

**FIGURE 2:**
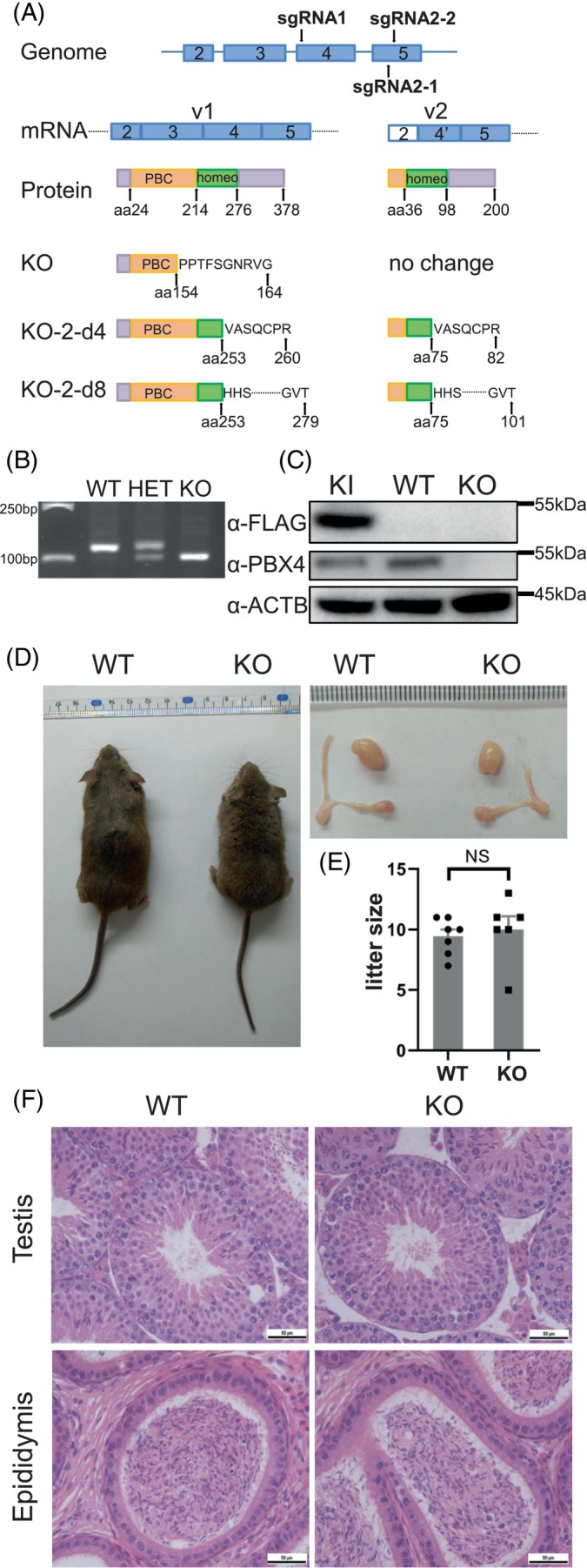
*Pbx4* knockout (KO) does not affect mouse spermatogenesis. (A) Schematic illustration of the predicted effects of *Pbx4* KO on the proteins encoded by transcript variant 1 (v1) and 2 (v2). Gene KO using sgRNA2‐1 and sgRNA2‐2 resulted in two mutant alleles KO‐2‐d4 and KO‐2‐d8, which were confirmed by sequencing. The corresponding predicted truncated proteins were labelled by the names of these two alleles. (B) Genotyping results of WT, HET (heterozygous deletion) and KO (homozygous deletion) mice by PCRs with 120F and 120R primers. (C) Validation of KO of PBX4 by Western blotting with α‐PBX4. The PBX4‐FLAG knockin (KI) mouse was used as a control for the PBX4 signal on Western blot. The original full image is shown as Figure S3D. (D) The morphology of WT and KO mice and their testes and epididymis. (E) Comparison of litter sizes of WT and KO mice. (F) Haematoxylin and eosin staining of testicular and epididymis sections in WT and KO mice. Scale bar, 50 μm.

We generated conventional KO mouse lines by using the CRISPR‐Cas9 technology to study its function in spermatogenesis. A small guide RNA (sgRNA1) was designed to target the genomic region corresponding to the PBC domain in exon 4 (Figure 2A). An allele with a 19‐bp deletion was detected by PCR in a founder mouse and confirmed by sequencing (Figure S4A). This founder mouse was crossed with WT mice to generate the heterozygous offspring which were further bred to generate the homozygous F2 KO mice (KO mice hereafter representing homozygous KO mice of this mutant allele at or after F2) (Figure 2B). Consistent with the predication that this 19‐bp deletion results in a frameshift and premature termination of the translation, PBX4 was confirmed to be absent in the testicular lysate of the KO mice by Western blotting using α‐PBX4 (Figure 2C, see Figure S3D for the original image).

Most male and female KO mice were infertile because they were unable to mate due to their abnormal physical conditions and behaviours (see next section for details) as indicated by the absence of virginal plugs in the females when either KO males and WT females or WT males and KO females were caged together. However, the testes of all KO mice were as big as the WT ones (Figure 2D). A small number of male KO mice sired offspring, and the average litter size of these fertile KO males were similar to that of the WT males (Figure 2E). In the haematoxylin and eosin‐stained testis and epididymis sections of the KO mice, all types of spermatogenic cells were present with no apparent anomalies (Figure 2F).

Since the 19‐bp deletion is located in the predicted coding region of mRNA variant 1 but not of variant 2 (Figure 2A), the undisrupted spermatogenesis of the KO mice may be due to a complimentary effect of a hypothetical shorter protein isoform encoded by mRNA variant 2 despite that this predicted isoform has not been detected by Western blotting (Figure 1D). To test this hypothesis, we used another two gRNAs that target the coding regions of both variant 1 and 2 mRNAs (sgRNA2‐1 and sgRNA2‐2) and generated a female founder mouse containing two mutant alleles with a 4‐bp and an 8‐bp deletion (KO‐2‐d4 and KO‐2‐d8; Figure S4B), both of which are predicted to result in frameshifts and premature termination of translation of both the full‐length and the hypothetical shorter PBX4 isoforms (Figure 2A). These two mutant alleles were passed to their F2 homozygous mice, which were collectively named KO‐2 mice, for phenotypical evaluations. Again, both the male and female KO‐2 mice were fertile, and spermatogenesis in males was normal (Figure S5). These results indicate that either PBX4 has no function in spermatogenesis or it has a function, of which the loss in KO mice may be compensated by other PBC proteins (see Section 3).

### 2.3 *Pbx4* KO mice undergo growth retardation, premature death and defective hindlimb development after birth

**FIGURE 3:**
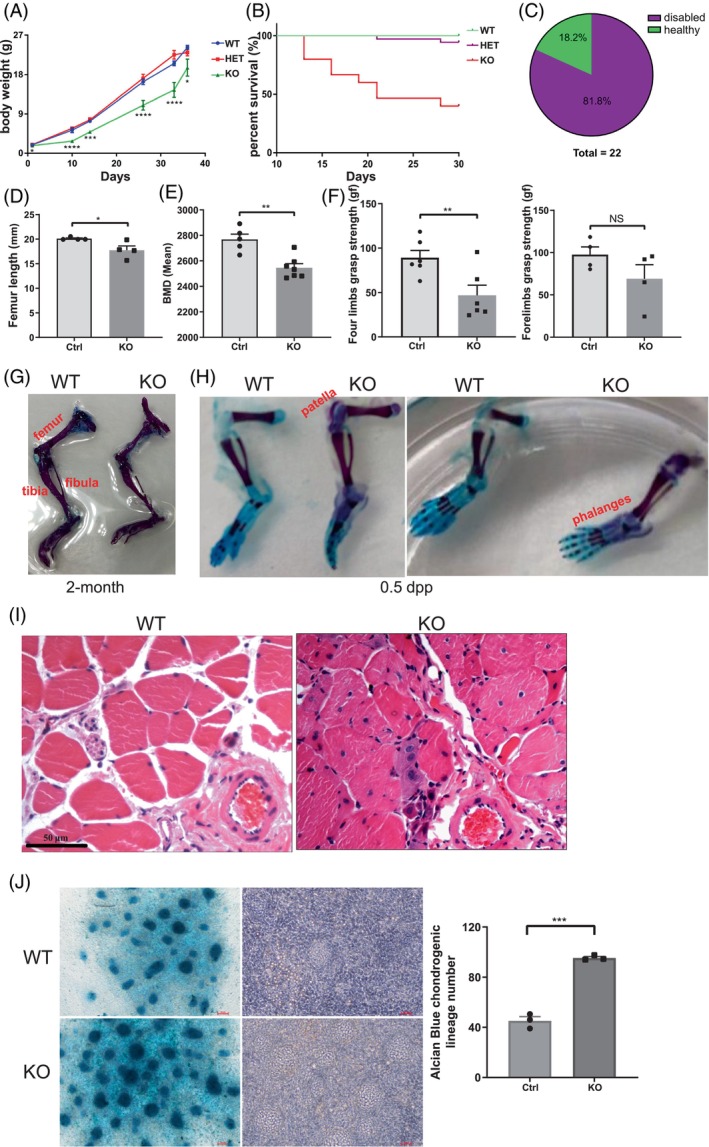
Pbx4 deletion resulted in growth delay, premature death and hindlimb disability in mice. (A) Growth curves of WT, HET and KO mice, *n* ≥ 3. (B) Survival curves of WT (*n* = 17), HET (*n* = 36) and KO (*n* = 15) mice. (C) The percentage of disabled and healthy KO mice. (D–F) Phenotypic evaluation of KO mice in femur length, femoral bone mineral density and limb grasp strength. Ctrl, control group that includes both WT and HET mice at the same age as the KO adult mice, *n* ≥ 4. (G, H) Alcian blue (cartilage)/alizarin red (bone) staining of WT and KO mice. (I) HE staining results of rectus femoris of WT and KO mice. Scale bar, 50 μm. (J). Alcian Blue staining of in vitro differentiated chondrogenic nodes from E11.5 hindlimb bud mesenchymal cells. Left panel, stained cells. Middle panel, unstained cells. Right panel, quantitative results of stained differentiated chondrogenic nodes. Note that stained and unstained pictures are not from the same area. Scale bar, 100 μm. *n* = 3. *, **, ***, **** represent *p*‐values less than 0.05, 0.01, 0.001, and 0.0001, respectively.

In order to reduce biometric variations caused by genetic background instability of the early generations of the C57B6/ICR KO mice, we back‐crossed these heterozygous or homozygous mutants with the ICR WT mice to reach at least 99% pure ICR background for further phenotypic evaluations. The ratios of the F2 offspring with the +/+, +/− and −/− genotypes were 1:2:1, indicating that there was no embryonic death of the mutant mice. Neonatal mice of the different genotypes were indistinguishable in terms of physical appearance and behaviour at the first week postpartum. However, growth retardation and inactivity were observed in KO mice but not in heterozygous mutants since 7 days postpartum (dpp) (Figure 3A). The KO mice died of bad health conditions including reduced weight, inactivity and hair loss mainly at two stages. One was between days 7 and 20 when KO pup mice became inactive to move, and they probably were unable to get enough milk from the mother during their competition with their WT and heterozygous siblings. The other was 4 months after birth when their health deteriorated quickly probably due to lack of movement and anaemia. About half of the KO mice died within 1 month after birth and most of them died before 6 months of age (Figure 3B).

About 18% of the KO mice were as normal as their WT littermates in terms of appearance and activity (Figure 3C). The remaining 82% were visually abnormal with inactivity, and they were not able to stand on their hindlimbs and their hindfeet were deformed to point backwards since 6–8 weeks after birth (Videos 1 and 2). Further phenotypic evaluations were carried out by comparing KO mice and WT and heterozygous littermates, which were named control mice collectively. The KO mice had shorter femurs, lower bone mineral density (BMD), and weaker total grasp strength than control littermates while their grasp strength of the forelimbs was indifferent from the control group (Figure 3D–F). Therefore, we inferred that the grasp strength of the hindlimbs of the KO mice was weaker than the control mice supported by the observation that the KO hindlimbs were smaller and thinner than WT ones in adult mice (Figure 3G). We reasoned that the defective hindlimbs of the KO mice might originate from certain bone abnormality at an earlier developmental stage. Therefore, we conducted skeleton Alcian blue/Alizarin red staining of the 0.5 dpp KO mice and found that ossification in both patella and phalanges occurred prematurely, and the cartilages of knee joint and ankle joint became ossified in KO mice (Figure 3H). We also prepared cross‐sections of the rectus femoris of WT and KO mice. HE staining indicated that the boundaries of muscle fibres become unclear and the number of cell nuclei per unit area increases, indicating abnormal development and/or atrophy of limb muscles (Figure 3I).

As skeletal progenitors are derived from resident limb bud mesenchymal cells that can differentiate in vitro into the same cellular lineages present in the limb, we isolated limb bud cells from E11.5 KO and WT mice and compared their abilities to differentiate into chondrogenic lineage. Interestingly, more chondrogenic nodes were observed when KO limb bud cells were induced to differentiate (Figure 3J). It seemed that the embryonic KO mice underwent premature limb development resulting in earlier ossification in both patella and phalanges and abnormal ossification of the joint cartilage.

We next carried out RNA‐sequencing (RNA‐seq) analysis to identify genes that are potentially regulated by PBX4 in hindlimb bud cells of the E11.5 mice by comparing gene expression in KO vs WT mice. A total of 424 differentially expressed genes (DEGs) were identified (*p* < 0.05). The top three enriched gene ontology (GO) biological process (BP) terms in the down‐regulated DEGs, i.e., genes that are positively regulated by PBX4, are ‘embryonic digit morphogenesis’, ‘heart development’ and ‘multicellular organism development’. Four terms related to appendicular development (‘embryonic digit morphogenesis’, ‘embryonic forelimb morphogenesis’, ‘embryonic limb morphogenesis’ and ‘embryonic hindlimb morphogenesis’) are seen among the terms with FDR < 10% (Table S1 and Figure S6). In comparison, although we also see term ‘skeletal system development’ among the enriched terms in up‐regulated DEGs, the top three terms in this set are ‘blood coagulation’, ‘haemostasis’ and ‘collagen fibril organisation’. Therefore, genes that are positively and negatively regulated by PBX4 in limb bud cells are basically different while they overlap by those involved in skeletal system development supporting the role of PBX4 in hindlimb development.

### 2.4 Single‐cell RNA‐sequencing data reveals *Pbx4* expressed in embryonic limb cells and spermatogenic cells

**FIGURE 4:**
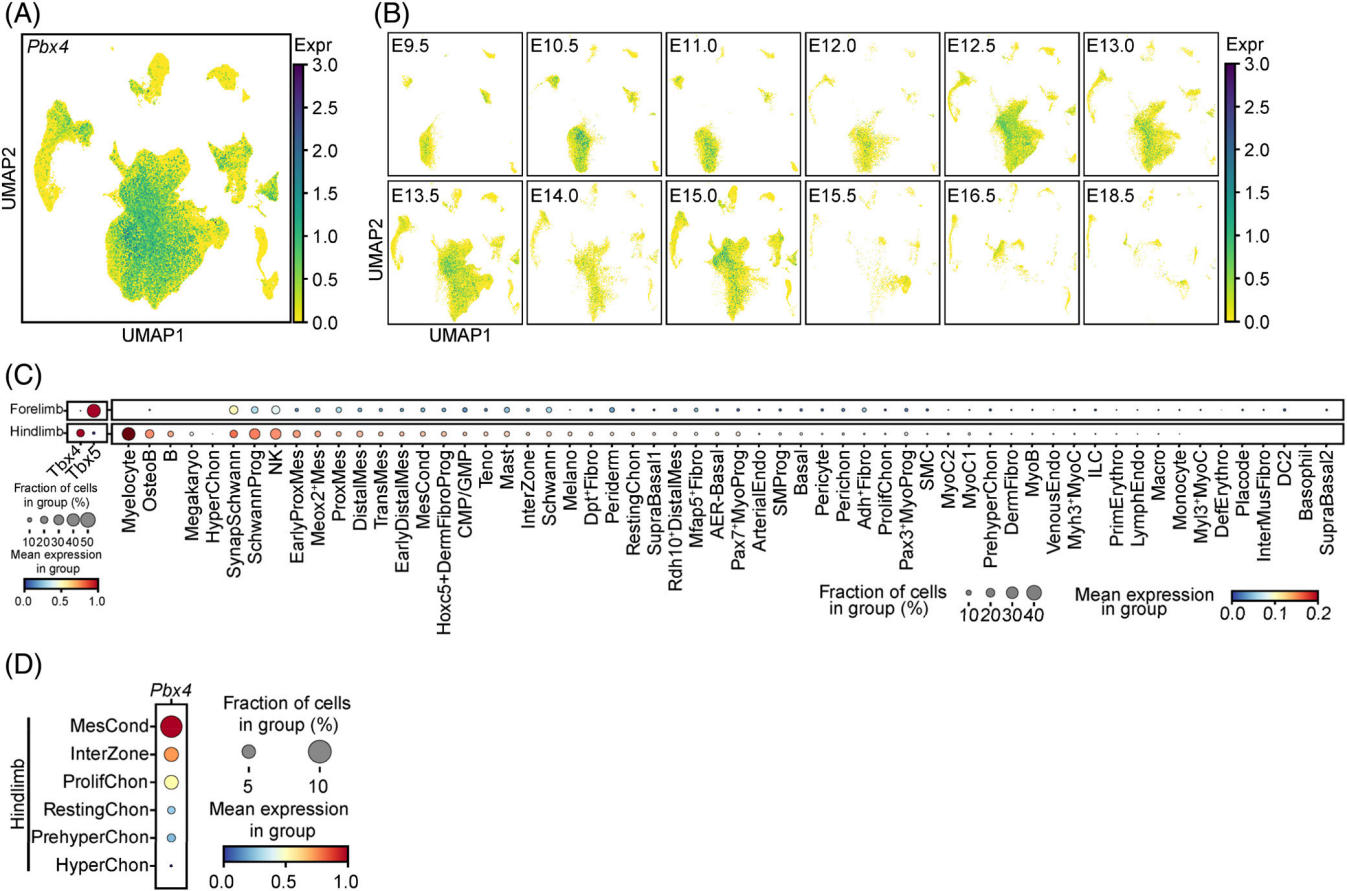
The expression level of *Pbx4* in mouse limb at single‐cell resolution. (A) Uniform manifold approximation and projection (UMAP) plot showing the expression level of *Pbx4* in cell atlas of mouse limb. The expression level is the normalised and logarithmic value of raw counts. (B) UMAP plot showing the expression level of *Pbx4* in cell atlas of mouse limb at each stage. E9.5 means embryonic day 9.5. (C) Dot plot showing the differentially expression level of *Pbx4* between forelimb and hindlimb in each cell type of mouse limb. *Tbx4* and *Tbx5* are the marker genes for hindlimb and forelimb, separately. For *Tbx4* and *Tbx5*, the expression level is standardised between 0 and 1 by gene. For *Pbx4*, the expression level is the normalised and logarithmic value of raw counts. (D) Dot plot showing the expression level of *Pbx4* in cell types of cartilage of mouse hindlimb. The expression level is standardised between 0 and 1 by gene. AER, apical ectodermal ridge; ArterialEndo, arterial Endo; ArtiChon, articular Chon; Chon, chondrocyte; ChondroProg, chondrogenic progenitor; CMP/GMP, common myeloid progenitors/granulocyte‐monocyte progenitors; DC2, Dendritic Cell 2; DefErythro, definitive erythrocyte; DefReticulo definitive reticulocyte; DermFibro, dermal Fibro; DermFibroProg, DermFibro progenitor; Endo, endothelial; Fibro, fibroblast; HyperChon, hypertrophic Chon; InterMusFibro, muscle interstitial Fibro; LMPP/ELP, lymphoid‐primed multipotent progenitor/early lymphoid progenitors; LympEndo, lymphatic Endo; Macro, macrophage; Megakaryo, megakaryocyte; Melano, melanocyte; Mes, mesenchyme; MesCond, mesenchymal condensate cell; MyoB, myoblast; MyoC, myocyte; MyoProg, myogenic progenitor; NK, Natural killer; OCP, osteochondral progenitor; OsteoB, osteoblast; PeriChon, perichondrium; PrehyChon, prehypertrophic Chon; PrimErythro, primitive erythrocyte; ProlifChon, proliferating Chon; ProxMes, proximal Mes; SchwannProg, Schwann progenitor; SMC, smooth muscle cell; SMProg, smooth muscle progenitor; SynapSchwann, synaptic Schwann; Teno, tenocyte; TenoProg, Teno progenitor; TransMes, transitional Mes; VenousEndo, venous Endo.

We were unable to detect PBX4‐FLAG in embryonic limb cells or other cell types using whole‐mount immunostaining with the α‐FLAG antibody on the E11.5 embryos probably because the antibody cannot recognise the antigen well or the protein level or the fraction of expressing cells is low (Figure S7A) (see Section 3). Nevertheless, the *Pbx4* mRNA was detected in the limb buds of both forelimbs and hindlimbs, consistent with the result of a previous study directly submitted to the MGI database (Figure S7B; https://www.informatics.jax.org/image/MGI:3501611).^14^ To identify *Pbx4*‐expressing cell types, we carried out single‐cell RNA‐sequencing analysis of limb cells from E9.5 to E18.5 (Figure 4, Figure S7C,D) (http://biorxiv.org/content/early/2022/04/28/2022.04.27.489800.abstract).^16^, ^17^ Among the many cell types, *Pbx4* is expressed in a considerable number of them including myelocyte, osteoblast, B, synaptic Schwann cells, Schwann progenitor cells, NK cells, some mesenchymal cell types (early proximal mesenchymal cells, Meox2+ mesenchymal cells, proximal and distal mesenchymal cells, transient mesenchymal cells and early distal mesenchymal cells), and some cell types of cartilage (mesenchymal condensate cells, InterZone cells, proliferating chondrocyte, resting chondrocyte, prehypertrophic chondrocyte and hypertrophic chondrocyte) (Figure 4C,D). Interestingly, the fractions of some *Pbx4*+ cells, e.g., myelocyte and osteoblast, are higher in the hindlimb than in the forelimb, consistent with the defects in the former but not in the latter. We also conducted analysis of *Pbx4* expression in mouse tissues at single‐cell resolution and found that *Pbx4* positive cells are much more abundant in the testis of the 3‐week‐old mice followed by the testis of adult mice (Figure S8A).^18^ Among different testicular cells, *Pbx4*+ cells are the most abundant in spermatocytes followed by round and elongating spermatids (Figure S8B,C). These results not only confirmed the results by RT‐PCR and immunostaining detection of *Pbx4* expression in the testis and spermatogenic cells but also served as good positive controls for *Pbx4* expression detected in the other cell types in embryonic limbs.

### 2.5 *Pbx4* KO mice were anaemic

**FIGURE 5:**
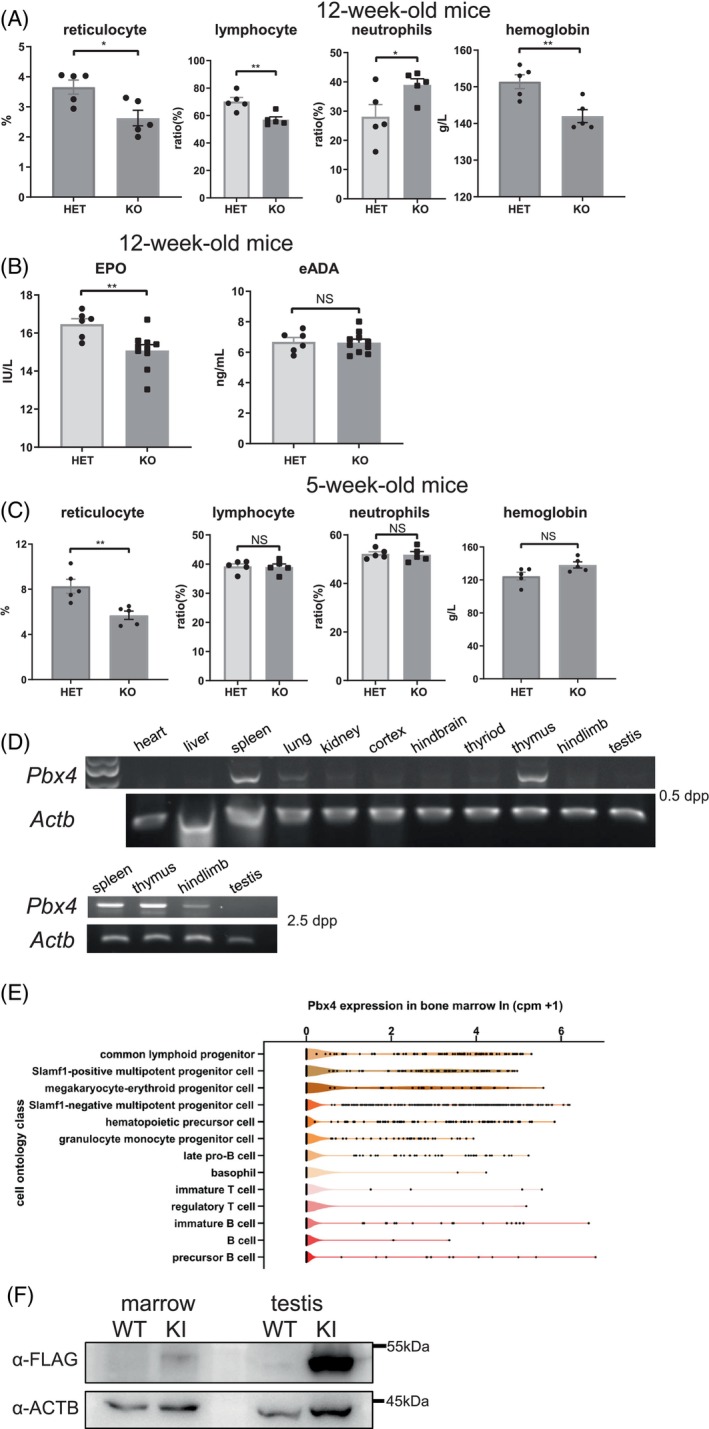
*Pbx4* KO mice were anaemic. (A–C) Selected items and quantitative results of blood test of KO and HET mice. *n* = 5 for results in (A) and (C); *n* ≥ 6 for (B). Results in (B) is generated by enzyme‐linked immunosorbent assay. eADA, erythrocyte adenosine deaminase; EPO, erythropoietin; *n* ≥ 6. (D) RT‐PCR detection of *Pbx4* in various organs of 0.5 and 2.5 dpp mice. (E) *Pbx4* expression in bone marrow cells by single‐cell RNA‐seq data from FACS sorted cells. These results are mined out from published dataset as described in the text. (F) The expression of PBX4 in testis and bone marrow of WT and KI mice was identified by Western blot with FLAG antibody. *, ** indicates that *p*‐values are less than 0.05, 0.01, respectively.

As growth retardation may also be caused by metabolic and/or haematopoietic anomalies, we next examined changes in several thyroid‐related hormones and blood cells in *Pbx4* KO mice. No difference was found in the levels of thyroid stimulating hormone, free triiodothyronine, free thyroxine, triiodothyronine and thyroxine between adult WT and KO mice (*p* < 0.05, Figure S9A). However, the KO adult (12‐week) mice had lower numbers of reticulocytes and lymphocytes, higher number of neutrophils and lower levels of haemoglobin and EPO compared with WT ones (Figure 5A–C). The number of reticulocytes in 5‐week mice is also lower than in WT mice at the same age. All the other blood test metrics except for haematocrit in 12‐week mice were not significantly different regardless of ages (Table S2A,B). These results suggested that the KO mice might have inherited bone marrow failure syndromes as early as 5 weeks after birth or even earlier.^19^

We then conducted RT‐PCRs to examine *Pbx4* expression in different organs of the neonatal (0.5 and 2.5 dpp) mice and found that it was expressed in spleen, thymus, hindlimb, lung, but not in the others including heart, liver and testis (Figure 5D). In order to further identify cells that express *Pbx4* in different organs/tissues of mice after birth, we also mined a dataset (GSE109774) that is about the transcriptomic characterisation of 20 mouse organs and tissues at single‐cell resolution.^20^ We found that *Pbx4* transcripts were detected in a number of cell types related to the haematopoietic and immune systems such as common lymphoid progenitor cell, Slamf1‐positive multipotent progenitor cell, megakaryocyte‐erythroid progenitor cell and haematopoietic precursor cell in the bone marrow (Figure 5E). *Pbx4* expression in these cells was consistent with the anaemic phenotype of its KO mice. In line with this, a low amount of PBX4‐3*FLAG expression was detected in bone marrow samples from KI mice by Western analysis (Figure 5F).

### 2.6 PBX4 targets to genes involved in gene regulation, limb development and haematopoiesis

**FIGURE 6:**
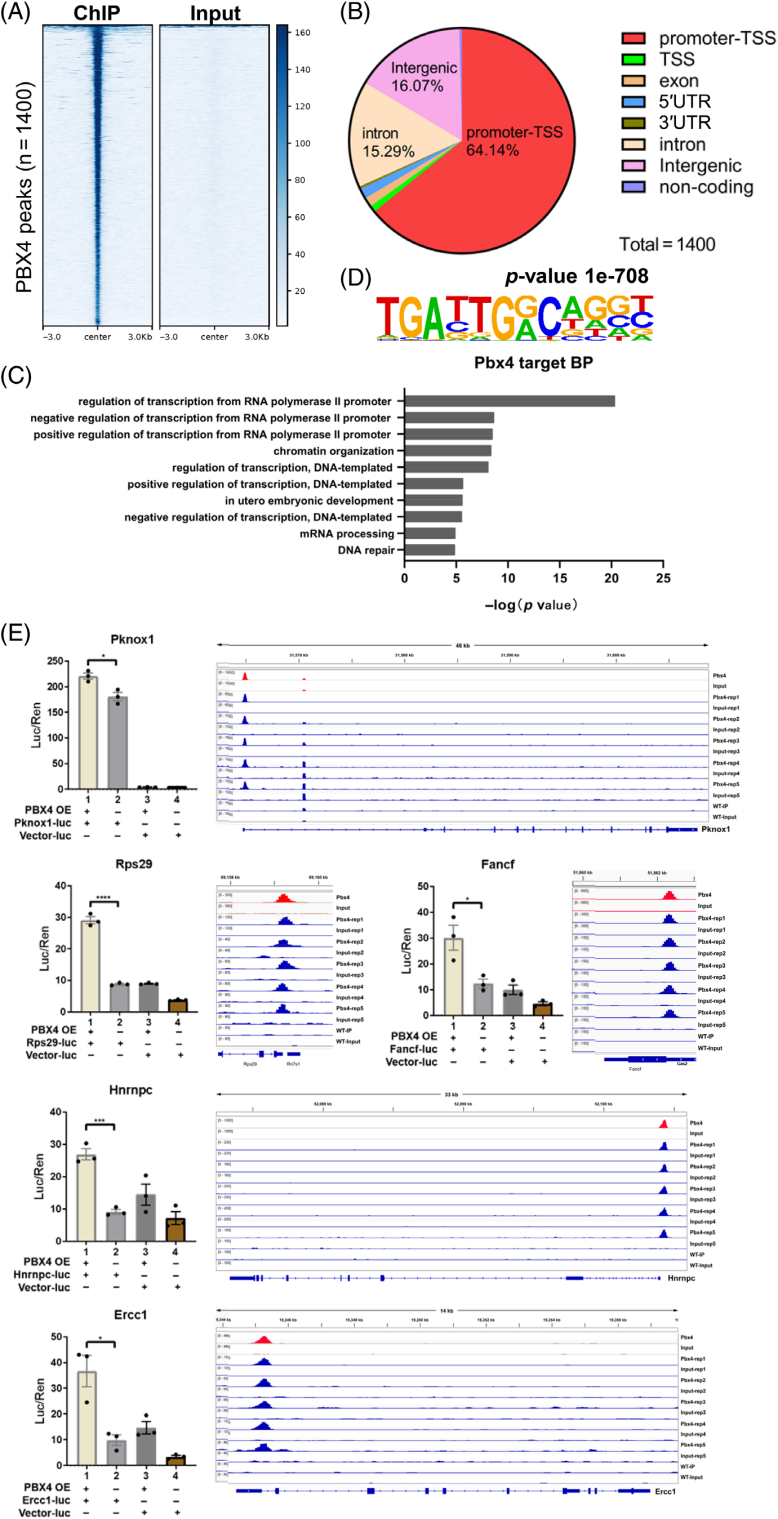
ChIP‐seq analysis of PBX4 biding sites in the mouse genome. (A) Heatmap of PBX4 ChIP‐seq peaks (*n* = 1400) in the mouse genome. Rows represent a 6‐kb window centred on PBX4 peak midpoints, sorted by the PBX4 ChIP‐seq signal intensities (averaged values from five biological replicates). (B) PBX4‐binding sites were classified by their genomic locations as indicated. (C) The top 10 enriched GO biological process (BP) terms for the putative PBX4 target genes. (D) The most significantly enriched de novo motif in PBX4 DNA‐binding sites identified by using HOMER. (E) Double luciferase experiment results (left) and ChIP‐seq tracks of PBX4 target genes. *n* = 3. *, **, ***, **** represent *p*‐values less than 0.05, 0.01, 0.001, and 0.0001, respectively.

To understand the molecular mechanism of PBX4 function, we carried out ChIP‐seq analysis of its binding site in the genome. Because it is only detected to be highly expressed in the adult testis, we used total testicular cells of adult mice to do the experiments. About 1400 binding sites (ChIP‐seq peaks) were identified from pooled analysis of five ChIP‐seq datasets generated from five different PBX4‐FLAG mice (Figure 6A). The peaks were highly and significantly enriched in promoters of 1328 genes (Figure 6B; Table S3). Almost all the top 10 enriched GO BP terms for the putative PBX4 target genes are related to gene expression regulation, and they are, for example, ‘negative/positive regulation of transcription from RNA polymerase II’, ‘chromatin organisation’ and ‘regulation of transcription, DNA‐templated’, suggesting PBX4 functions as a master regulator of gene expression (Figure 6C; Table S3). These GO terms cover a total of 397 genes, about 30% of the total target genes. Many of these target genes encode zinc finger proteins, and some are for Three Amino acids Loop Extension proteins such as PKNOX1/PREP1 and PBX3.

De novo motif enrichment analysis on the ChIP‐seq peaks revealed that the most significantly enriched motif (*p* value = 1e‐708; fold enrichment = 15) were identified in 60% of the peaks, and the best match of this motif is that of the PKNOX1 binding site (Figure 6D, Tables S3 and S4). Moreover, enrichment analysis of the known motifs showed that, among the top 10 enriched known motifs, 8 were for HD‐containing proteins such as PKNOX1, PBX3, PBX1, PBX2, MEIS1, EN1, TGIF1 and HOXA9 (Tables S3 and S4). PKNOX1 was initially identified as a functional partner of PBX proteins and plays important roles in haematopoiesis.^21^, ^22^ These results indicated that PBX4 is very likely to regulate gene expression by partnering with other HD‐containing proteins.

Interestingly, a sharp peak of PBX4 was observed at the promoter region of Pknox1 and the interaction of PBX4 with this region to activate gene expression was confirmed by the luciferase assays (Figure 6E). We also found that PBX4 bound to promoters and activate the expression of a number of genes involved in limb development such as *Foxo1, Foxo3, Runx1, Fgfr2* and *Mtor* ^23^ and genes in haematopoiesis such as *Rps29*, *Fancf*, *Hnrnpc* and *Ercc1* ^24^ as shown by the ChIP‐seq peak distribution plots and the luciferase assay results (Figure 6E; Table S3).

## 3 DISCUSSION

In the present study, we examined the expression and function of PBX4, the last member of the mammalian PBC family, of which the physiological function had not been reported. We found that spermatogenesis was not disrupted by its gene KO although PBX4 was highly expressed in meiotic and postmeiotic germ cells in adult male mice. Normal spermatogenesis in KO mice is supported by the lack of abnormal testicular morphological development and the unaffected litter size of some healthy KO male mice; despite that, in‐depth analysis of spermatogenesis warrants further investigation. Instead, abnormal hindlimb development and anaemia were observed in the KO mice. Genes differentially expressed in limb bud cells of KO and WT mice were identified and they are enriched in GO terms related to limb development. *Pbx4* mRNA was detected in cells related to limb development and haematopoiesis at the single‐cell resolution and low‐level protein was also detected in bone marrow by Western blotting. More importantly, a large number of target genes, of which some are involved in gene regulation, limb development and haematopoiesis, were identified by ChIP‐seq analysis.

It is common that the KO of an evolutionarily conserved gene results in no apparent phenotype in caged mice. This phenomenon is more often observed when spermatogenesis is studied. This is usually explained by two theories. One is that the KO phenotype cannot be revealed under a regular condition, and it can be apparent under a different condition, for example, when the mice live in the wild and experience survival stresses. The second one is that spermatogenesis is so important for the continuation of a species and a more robust safeguarding system is evolved in germ cells than in somatic cells. This system is likely activated in the gene KO mice by the compensatory effects of other genes. The mechanism of compensation can be complicated depending on the developmental processes and lacks in‐depth studies in the field.

It is interesting that we identified 12 different alternatively spliced variants of *Pbx4* by molecular cloning followed by sequencing, but only one full‐length protein encoded by the longest mRNA variant was detected by Western blotting. The other variants either do not encode proteins or produce low levels of protein isoforms that are hard to detect. If the latter is true, it may explain why the KO mice display no defect in spermatogenesis as other protein isoforms may compensate the KO effects of the full‐length protein. However, this explanation is more likely false because the predicted protein isoforms of these splice variants lack either the PBC or the HD domain that are believed to be important for the function of the proteins.^25^ Moreover, that the deletion of proteins encoded by the most abundant v1 and v2 variants in our KO‐2 mice still has no effect on spermatogenesis further suggests that the lack of defect in spermatogenesis in KO mice is not due to the redundant protein isoforms of PBX4. Indeed, the spermatogenic cells are well known for generating spliced variants for a large number of genes, but very few of the variants are known to have molecular functions.^26^, ^27^ It has been proposed that regulation in transcription and splicing are globally relaxed to generate noisy transcripts due to drastic chromatin reconfiguration during spermatogenesis.^28^ As PBX4 proteins are highly conserved from zebrafish to humans and it is highly expressed in spermatogenic cells suggesting a role in these cells, the best explanation for the lack of apparent phenotype in spermatogenesis in its KO mice is that its lost function is compensated by other PBC proteins. In line with this, sequences of mouse PBX1‐4 are highly conserved (Figure S1A). Moreover, it is reported that ectopic expression of the fly pbx gene edx rescues the phenotype of zebrafish *pbx4* mutant embryos.^15^

PBX4 was first reported by Wagner et al.^7^ to be enriched in the XY body in spermatocytes. When we examine the immunostaining picture in that study closely, we find that the authors might mislabel the acrosome in spermatids as the XY body in spermatocytes. Indeed, our home‐made α‐PBX4 recognises not only PBX4 but also some other proteins nonspecifically on Western blots, and it labels the acrosome nonspecifically in the sections of both WT and KO testes (Figure S3D,E). In contrast, the α‐FLAG only recognises FLAG‐tagged PBX4 specifically on Western blots. Immunostaining using this antibody reveals that PBX4 distributes in the nuclei of both spermatocytes and spermatids evenly, consistent with the staining pattern of most nucleus‐localised transcription factors.

That PBX4 plays a role in hindlimb development is reminiscent of the observations that PBX1 and PBX2 execute hierarchical, overlapping and interactive functions in appendicular skeleton development in previous studies using single or compound gene mutant mice.^12^, ^25^ More specifically, PBX4, like PBX1, seems to control the proliferation of several types of progenitor cells during limb development as indicated by both the abnormal ossification in several regions of the hindlimb and the hypertrophic formation of chondrogenic nodules from mesenchymal cells in vitro.^10^ Based on the similar phenotypes of the different *Pbx* gene KO mice, it is apparent that PBX proteins have evolutionarily conserved functions. That PBX4 plays a role in hindlimb development is also supported by the genes differentially expressed between KO and WT limb bud cells typical of limb development indicated by GO analysis. Although we were unable to identify specific cell types by immunostaining probably due to the low protein abundance, the presence of these *Pbx4* expressing cells were consistently indicated by in situ hybridisation assay and single‐cell RNA‐sequencing analysis. Therefore, it is likely that PBX4 is expressed and executes its function in cells related to limb development as early as at the stage of E9.5–18.5 but abnormal limb development in its KO mice is only apparent until after birth due to a long sequential regulatory pathway initiated by PBX4. That *Pbx4* KO mice are anaemic with decreased number of reticulocytes and lower level of EPO reveals that PBX4 is also involved in haematopoiesis similar to PBX1 that is required for maintenance of definitive haematopoiesis.^29^ Consistent with function of *Pbx4* gene in haematopoiesis, both its transcripts and protein were detected in several cell types in the bone marrow.

The results of our ChIP‐seq analyses provide several interesting and important clues for the molecular functions of PBX4. (1) PBX4 may act as a master regulatory transcription factor because its binding sites are mainly localised in the promoters of genes that are enriched in GO terms related to gene regulation. This is consistent with the molecular role of the PBC family members that usually act as cofactors of many other transcription factors including the Hox proteins that are at the top level of the transcription regulatory hierarchy.^1^, ^25^ (2) PBX4 also directly regulates non‐transcription factor genes that execute functions in particular developmental processes such as embryonic development (see the 7th enriched GO term in Figure 6C) and haematopoiesis (see tab ‘limb and hematopoiesis genes’ in Table S3). It is noteworthy that sharp ChIP‐seq peaks are located in the promoters of a number of genes that are involved in haematopoiesis and the regulatory role of PBX4 through these sites are confirmed by luciferase assays (Figure 6E). (3) PBX4 binds to a motif highly enriched in the ChIP‐seq peaks (*p* value = 1e‐708; fold enrichment = 15; identified in 60% of the peaks) with the core ‘TGAT’ tetranucleotides that has been recognised as the typical binding sites of other PBX proteins.^1^ As this motif is most similar to that of PKNOX1, PBX4, like other PBC members, probably also functions in partnership with other MEIS and/or HOX proteins.^25^

It is interesting that our ChIP‐seq experiment was conducted using testis cells, yet genes involved in non‐testicular processes such as haematopoiesis are identified as target genes. On a second thought, this means that PBX4 tends to target to the same set of genes that play context‐dependent roles in different organ/tissue cells. Unfortunately, the *Pbx4* single gene KO does not reveal the function of PBX4 in spermatogenesis. It may be informative to generate compound gene mutant mice with *Pbx4* KO in different combinations with the conditional ablation or reduction of the other *Pbx* genes to test whether these PBX proteins indeed play a role in spermatogenesis.

## 4 MATERIALS AND METHODS

### 4.1 Mice

The breeding and experiments of all animals used in this study were carried out in compliance with the relevant regulations of the Animal Welfare Committee of the Institute of Zoology, Chinese Academy of Sciences.

The generation of *Pbx4* mutant mice was conducted by following the procedure described in a previous study.^30^ Briefly, Cas9 mRNA (10 ng/μL) and sgRNAs (5 ng/μL) were injected into the cytoplasm of zygotes obtained by mating DBA males with superovulated C57BL/6J females. Injected embryos were transferred to the uteri of ICR surrogate female mice, and offsprings were genotyped by PCRs. The primers used in genotyping are listed in Table S5. For genetic background purification, heterozygous male mutants were repeatedly crossed with ICR female mice at each generation via a common breeding scheme known as ‘backcrossing’ till the genetic background was shifted towards a purity of 99.2% of the ICR background. For PBX4‐FLAG KI mice, donor DNA fragment containing the left and right homologous arms flanking the 3 × FLAG coding sequence was cloned into a plasmid modified from a knockin targeting construct (#59721, addgene) and amplified in bacteria. It was then cut off from the plasmid by enzymatic digestion and purified by using a silica column (28704, QIAGEN) (12 ng/μL) and injected together with Cas9 mRNA (10 ng/μL) and sgRNAs (5 ng/μL) to the two‐cell embryos. To minimise the chance that the FLAG tag changes the protein localisation and function, we used the I‐TASSER (Iterative Threading ASSEmbly Refinement) web tool (https://zhanggroup.org/I-TASSER), which was ranked as the No 1 server for protein structure prediction, to examine the predicted structure of PBX4, and found that its C‐terminus is exposed as a free end that may not play an important role in stabilizing the protein structure; we then added a minimum of 3 × FLAG plus a linker sequence, which together is about 30 amino acid long, to this end.

### 4.2 Fertility test

One adult male mice (2–5 months of age) was housed with two 8‐week‐old C57BL/6J WT female mice for 6 days. Female mice were checked for the presence of vaginal plugs every morning to examine mating activity. The procedure for the same male mouse was repeated two–three times with different female mice. The numbers of litters per male were recorded and calculated.

### 4.3 Antibody generation

A polyclonal antibody against mouse PBX4 (α‐PBX4) was produced commercially by ABclonal Technology (Wuhan, China). Briefly Japanese White Rabbits were injected with two synthetic peptides each of which was coupled with keyhole limpet haemocyanin. The two peptides are STVTKARRPRGQSSC (corresponding to amino acids 292–306 of the PBX4) and SPAGESGSFNWDAASN (corresponding to amino acids 362–378 of the PBX4). The rabbits were injected with the peptides five times with 2‐week intervals in‐between. The antiserum was subjected to affinity purification using the antigen to obtain the polyclonal antibody α‐PBX4.

### 4.4 Western blotting

In order to avoid the interference caused by antibodies in the blood of mice, it is necessary to use normal saline for the sampling of various tissues in mice, especially the liver, brain, heart and other organs that are susceptible to blood contamination. The bone marrow samples contain a large amount of immunoglobulin G (50 kDa) similar in size to PBX4‐3*flag (expected 46 kDa, electrophoresis *c*. 50 kDa), which will interfere with the results of Western blot experiments and need to be removed. We refer to the preclear step of removing immunoglobulin with Protein G Beads in the co‐IP‐MS experiment reported.^31^ The total amount of protein in the bilateral femoral bone marrow of one adult mouse obtained was about 1 mg. After full digestion with 200 μL RIPA lysate for 30 min, 5 μL Protein G Beads, blocked by 1% BSA for 1 h, were added and incubated by rotation on a rotor at 4°C for 1 h. Tissues were collected and washed twice with cold PBS. Upon being minced into small pieces with tissue scissors, the tissues were transferred to T10 homogeniser (IKA T10 basic ULTRA‐TURRAX, Germany) in Radio Immunoprecipitation Assay lysis buffer (P0013B, Beyotime) containing protease inhibitor cocktail. The mixture of various tissues was homogenised on ice for 30–200 s and incubated on ice for 30 min and centrifuge at 120,00 g for 10 min at 4°C to remove insoluble cell debris. The tissue extract was boiled with 5 × SDS loading buffer for 10 min. According to the molecular weight of protein, electrophoresis was carried out on SDS‐PAGE gel containing different concentrations of separation gel (8%–12%), and then imprinting was carried out on polyvinylidene fluoride membranes (88518, Thermo Fisher Scientific). The membrane was blocked with 5% skim milk in PBST (phosphate buffered saline, 0.1% Tween20) at room temperature for 1 h and then incubated in the dilution of primary antibody at 4°C for 4 h or overnight. After washing with PBST for three times, membranes were incubated in horseradish peroxidase‐conjugated secondary antibodies (diluted with PBST) for 2 h at room temperature and then washed with PBST for three times at room temperature by shaking. The Western blotting was finally detected by SuperSignal West Pico Plus Chemiluminescent Substrate (34577, Thermo Fisher Scientific) and imaged on Universal Hood II system (Bio‐Rad, USA) or Tanon‐5200 Chemiluminescent Imaging System (Tanon Science & Technology, China).

### 4.5 Chromatin immunoprecipitation sequencing and analysis

ChIP‐seq experiment was conducted by following the procedure in a previous study.^32^ Briefly, testicular cells from adult mice were prepared using a two‐step digestion method.^33^ About five million testicular cells were cross‐linked with 1% formaldehyde for 10 min at room temperature (methanol‐free, Cell signalling technology, 12606P). The reaction was quenched by addition of 2 M glycine, followed by wash with cold PBS twice. Cells were suspended in cell lysis buffer (10 mM Tris–HCl [pH 8.0], 1 mM EDTA, 10 mM NaCl, 0.2% NP‐40 and 1 × protease inhibitor cocktail) and incubated for 25 min on ice. After centrifugation, the nuclei were lysed with ChIP lysis buffer (50 mM Tris–HCl [pH 8.0], 10 mM EDTA, 1% SDS and 1 × protease inhibitor cocktail) for 15 min on ice. Chromatin were sheared to 200–500 bp fragments by Qsonica Q800R3 instrument. The fragments were diluted to 10 × volume with ChIP dilution buffer (16.7 mM Tris–HCl [pH 8.0], 1.2 mM EDTA, 167 mM NaCl, 0.01% SDS, 1.1% Triton X‐100 and 1 × protease inhibitor cocktail) then cellular debris was removed by centrifugation. About 1/20 volume of fragments were saved as an input sample. Before immunoprecipitation, Dynabeads Protein G beads (1003D, Invitrogen) were washed three times in block solution (0.5% BSA in PBS) and then rotated with α‐FLAG (F1804, SIGMA‐ALDRICH) for 8 h at 4°C. The other fragments were incubated with antibody‐bound beads rotating at 4°C, O/N. The beads were sequentially washed with following buffers: twice with low‐salt wash buffer (20 mM Tris–HCl [pH 8.0], 2 mM EDTA, 150 mM NaCl, 1% Triton X‐100 and 0.1% SDS), twice with LiCl buffer (10 mM Tris–HCl [pH 8.0], 1 mM EDTA, 0.25 M LiCl, 1% NP‐40 and 1% deoxycholic acid) and twice with TE buffer (10 mM Tris–HCl [pH 8.0], 1 mM EDTA). Bound chromatin was eluted twice with 125 μL elution buffer (10 mM Tris–HCl [pH 8.0], 1 mM EDTA, 1% SDS, 0.1 M NaHCO_3_, 5 mM DTT) for 15 min at 65°C with vortex and then reverse‐crosslinking at 65°C for 8 h. After RNase A and proteinase K treatment, DNA were purified by phenol/chloroform extraction method. The ChIP libraries were constructed with NEBNext Ultra II DNA Library Prep Kit for Illumina (E7645 and E7335, NEB), and qualified libraries were sequenced with an Illumina NovaSeq 6000 system to obtain paired‐end 150‐nucleotide (nt) reads.

The raw reads of ChIP‐seq were converted into a fastq file, and the sequencing quality was evaluated by FastQC; the reads were trimmed to remove the adapter sequence. The trimmed ChIP‐seq reads were mapped to UCSC mm10 genome by using Bowtie2 (v2.5.0) with default parameters. For PBX4, peak calling was performed using Pepr (v1.1.10) with default parameters and corresponding input as background, and the peak mapped to the blacklist (60) regions was removed. The annotation of genome location and repeat type is generated by HOMER (v4.11), and the heatmap is generated by the command line version of deepTools (v3.5.0). The 5‐kb box of the whole genome was calculated by deepTools, and the scatter plot was drawn by plotCorrelation using Pearson method. ChIPseeker (v1.24.0) was used to generate the distribution of PBX4‐binding sites on chromosomes, and HOMER (v4.11) was used to identify the rich motif in PBX4 peak under the default setting.

### 4.6 RNA‐sequencing analyses

Hindlimb buds were extracted from E11.5 embryos using forceps under a stereoscope. Total RNAs were isolated using TRIzol (15596‐026; Invitrogen). High‐throughput sequencing libraries were constructed using the NEB‐Next Ultra RNA Library Prep Kit for Illumina (E7760, NEB). Sequencing was performed on the Illumina HiSeq X10 platform (Novogene). The sequencing reads were mapped to the mouse genome (UCSC mm10) with TopHat (v2.0.6). Differentially expressed genes were identified by Cufflinks (v2.0.2).

### 4.7 Histological evaluation and immunostaining

Haematoxylin and eosin staining of testis sections was carried out by following a standard protocol.^31^ For immunofluorescence or immunohistochemistry experiments, paraffin sections were dewaxed, rehydrated and incubated with sodium citrate buffer (pH 6.0), at 98°C for 15 min to repair the antigen. Samples were diluted with 5% bovine serum albumin (BSA) in phosphate buffered saline (PBS) and blocked at room temperature for 1 h. The primary antibody was diluted with 5% BSA/PBS (1:100 dilution for both α‐PBX4 and α‐FLAG), and the sections were incubated overnight at 4°C. After washing three times with PBS, the diluted fluorescently labelled or horseradish peroxidase‐coupled secondary antibody (1:200 dilution) was incubated with the sections at room temperature for 1 h. For immunofluorescence, DNA was stained with 4′, 6‐diamino‐2‐phenylindole diluted with PBS. For immunohistochemistry, dilute DAB solution is used as the developer and the sections are covered for 1–5 min at room temperature. The reaction was then immediately stop with flowing H_2_O. The nuclei were stained with haematoxylin, and the slides were dehydrated in a gradient manner with ethanol, and made transparent with xylene. Images were taken using an optical microscope (ECLIPSE 80i, Nikon).

### 4.8 Skeletal preparation

Here, 0.5 dpp suckling mice were processed and stained for bone and cartilage as described.^34^ Mouse samples were kept in H_2_O for 1–2 h at room temperature and heat‐shocked at 65–70°C for 2 min. The skin was taken off, and the abdominal and thoracic viscera were removed using forceps. The samples were then fixed in 95% ethanol overnight. Transfer the mice to acetone and incubate overnight at room temperature to remove fat. The samples were rinsed with deionised water, stained using Alcian blue staining solution (150 mg/mL Alcian blue 8GX in 80% ethanol and 20% acetic acid). The samples were washed with 70% ethanol for 6–8 h for five times and then treated with 1% potassium hydroxide overnight or until the tissues were visibly cleared. The samples were stained with alizarin red solution (50 mg/mL alizarin red S in 0.2% potassium hydroxide) overnight. The samples were cleared in clear solution (1% potassium hydroxide, 20% glycerol) for 2 day, and kept in 80% glycerol.

### 4.9 In vitro differentiation of mouse hindlimb bud mesenchymal cells into chondrogenic micromass

E11.5 female mice were sacrificed, and the uterus was dissected.^35^ The anterior and posterior limb buds of the embryos were separated under a microscope, and the anterior quarter was removed. The hindlimb buds were digested with 1 mg/mL collagenase II/PBS at 37°C for 15 min. The single‐cell suspension was filtered through a 45 μm filter and centrifuged at 400 g for 6 min; the cells were suspended in culture medium (DMEM‐LG [C11330500BT, Invitrogen] supplemented with 10% fetal bovine serum, 2 mM l‐glutamine, 0.1 mM non‐essential amino acids, 1% penicillin/streptomycin) at a density of 10 million cells per mL. Then, 10 μL of each cell suspension was added to the centre of a 24‐well plate, incubated in a 37°C incubator for 30 min and then 1 mL of medium was gently added to each well. The medium was changed every other day, and Alcian staining was conducted at day 5 of culture.

### 4.10 Luciferase assay

*Pbx4* cDNA was cloned into a pFLAG‐CMV‐4 vector, and the PBX4‐targeted regions for genes to be studied were amplified from mouse genomic DNAs and cloned into a PGL4.23‐luciferase vector (E8411, Promega). Then, 200 ng *Pbx4*‐expressing plasmid, 100 ng PGL4.23‐luciferase plasmid‐containing DNA element to be examined, and the 20 ng pRL‐TK‐Renilla plasmid were cotransfected into 293FT cells on 48‐well plates using the X‐Transcell reagent (bjyf‐Bio technology) following the manufacturer's protocol. Then, 48 h after transfection, luciferase activity was examined using the Dual‐Luciferase Report Assay System (Promega) on the Synergy4 (Bio‐Tek) platform.

### 4.11 MicroCT test BMD and femur length

X‐ray microtomographic equipment, a PE Quantum FX microCT Imaging System (PerkinElmer, USA), was used to determine the bone growth and bone density of mouse. The X‐ray source was set at 90 KV and 160 μA. Mice were scanned up to a field of view of 24 mm. Data analyses were performed using analyze11 software (Mayo Clinic, USA). During the experiments, the animals were anaesthetised with a mixture of isoflurane (0.75%–1.5%) and oxygen (100%). Mouse breathing rate and body temperature were monitored.

### 4.12 Grip strength test

The mouse is placed over a base plate in front of a grasping tool (either T‐shaped, or grid) with an adjustable height. The Grip Strength Meter (47,200, UGO BASILE srl, ITALY) automatically measures grip strength (i.e., peak force and time resistance) of forelimb or four limbs (via the grid) of mice.

### 4.13 Isolation of testicular cells by gravity sedimentation

Testicular cells were isolated by following a protocol as described previously.^36^ Briefly, testes were digested with collagenase (1 mg/mL, 5 min, 37°C) and trypsin (0.25%, 5 min, 37°C) after removal of the albuginea. Dispersed cells were suspended in DMEM medium (product no. 11965‐092; GIBCO) with 0.5% BSA (product no. a9647; Sigma) and then loaded into a glass cylinder on top of 600 mL of BSA solution of 2%–4% BSA gradient in DMEM. After 3 h of sedimentation, the cell fractions were collected from the bottom of the cylinder. Different types of cells were isolated from mice of different ages as followings: Sertoli cells from 6‐dpp mice, preleptotene spermatocytes and pachytene spermatocytes from 17‐dpp mice, round spermatids and elongating spermatids from 60‐dpp adult mice. The purity of each cell type was confirmed by quantitative real‐time reverse transcription polymerase chain reaction (RT‐PCR) assessment of their marker genes (Figure S1D). Primer sequences are listed in Table S5.

### 4.14 Whole‐mount in situ hybridisation

Digoxigenin labelled RNA probes were made, using PCR products as template and T7 RNA polymerases. E11.5 embryos were dissected and fixed with 4% paraformaldehyde. For each probe, one embryo was treated with 10 μg/mL Proteinase K for 30 min. The post‐hybridisation washes and antibody incubation were performed by following a protocol as described previously.^37^ Signals were developed with BM purple.

### 4.15 Analysis of single‐cell RNA‐sequencing data of mouse embryonic limb cells

The three datasets of mouse limb were downloaded from ArrayExpress (E‐MTAB‐10514), ENCODE portal (ENCSR713GIS) and Gene Expression Omnibus (GSE142425). Preprocessing included data normalisation (pp.normalize_per_cell with 10,000 counts per cell after normalisation), logarithmise (pp.log1p), highly variable genes detection (pp.highly_variable_genes and select for highly correlated ones as described in83) per batch and merging, data feature scaling (pp.scale), cell cycle and technical variance regressing (tl.score_gene_cell_cycle and pp.regress_out(adata, [‘S_score’, ‘G2M_score’, ‘n_counts’, ‘percent_mito’])) and principal components analysis (tl.pca with 100 components) performed using the Python package Scanpy (v.1.8.2). bbknn (v.1.5.1) was used to correct for batch effect between sample identities with the following parameters (n_pcs = 100, metric = ‘euclidean’, neighbors_within_batch = 3, trim = 299, approx = False). Following this, further dimension reduction was performed using Uniform Manifold Approximation and Projection (scanpy tl.umap with default parameters) based on the corrected neighbohood graph of bbknn. Leiden graph‐based clustering (scanpy tl.leiden with default parameters) was performed to obtain unsupervised cell classification.

### 4.16 Analysis of single‐cell RNA‐sequencing data of mouse multiple tissues and testis

The dataset was downloaded from GSE (GSE176063). The following quality control steps were performed: (i) cells that expressed more than 3000 genes and contains more than 10,000 counts were excluded; (ii) cells in which over 20% of unique molecular identifier were derived from the mitochondrial genome were removed. Doublets were detected by using the Scrublet (v0.2.1). Data Preprocessing were performed as the same as the mouse embryonic limb. bbknn (v.1.5.1) was used to correct for batch effect between sample identities with the following parameters (n_pcs = 49, metric = ‘euclidean’, neighbors_within_batch = 3, trim = 200, approx = False). Following this, further dimension reduction was performed using Uniform Manifold Approximation and Projection (scanpy tl.umap with default parameters) based on the corrected neighbourhood graph of bbknn. Leiden graph‐based clustering (scanpy tl.leiden with default parameters) was performed to obtain unsupervised cell classification. The identification of marker genes for each cell cluster were performed by using DEAPLOG (with default parameters) (https://www.biorxiv.org/content/10.1101/2022.12.21.521359v1).

### 4.17 Mining of mouse single‐cell transcriptomic data of multiple tissues

Single‐cell transcriptomic data published by the Tabula Muris consortium were mined for the expression of *Pbx4* in cells of different adult mouse organs/tissues.^20^ The website associated with the published paper (https://tabula-muris.ds.czbiohub.org/) provides a search tool. In the search tool, select ‘FACS’ and ‘ALL’ for the Method and the Tissue attributes, respectively, type ‘*Pbx4*’ in the Gene input field; expression information and plots for *Pbx4* expression in different cell types are displayed. We observed that most of *Pbx4* expressing cell types are from the bone marrow. Therefore, we downloaded the expression data and replotted *Pbx4* expression in 13 cell types related to immune and haematopoiesis as shown in Figure 5E.

### 4.18 Statistical analysis

Statistical analysis was performed with Student's *t*‐test. The values were presented as mean ± SEM. Excel 2016 or GraphPad Prism 8.0.2 was used to perform statistical analyses. All the experiments reported were repeated at least three independent times. *p < 0.05, **p < 0.01, ***p < 0.001, ****p < 0.0001, NS, not significant.

## AUTHOR CONTRIBUTIONS

CH supervised the project, conceived and designed the study, wrote the manuscript. YN designed and performed the experiments, analysed the data and wrote the manuscript. SD generated the *Pbx4* gene KO and knockin mice. XiL conducted bioinformatic analyses. JC isolated spermatogenic cells. DX conducted ChIP‐seq experiment. XiaoL performed immunostaining and genotyping. YZ and JF performed the whole‐mount ISH experiment and revised the manuscript. BZ and HZ conducted the scRNA‐seq analysis of mouse limb bud cells and testicular cells and revised the manuscript.

## FUNDING INFORMATION

This work was supported by the National Key R&D Program of China (2018YFE0201100) and the National Natural Science Foundation of China (31970795).

## CONFLICT OF INTEREST STATEMENT

The authors declare no conflicts of interest.

## Supporting information


**Figure S1.** Sequence comparisons of PBC family proteins. (A) PBX1, PBX2, PBX3 and PBX4 alignment (left) and identity (right). (B) PBC family proteins conserved domains. (C) Multi‐alignment analyses and identity of PBX4 orthologues across 5 species.
**Figure S2.** *Pbx4* mRNA expression. (A) Structures of alternatively spliced variants of *Pbx4*, of which cDNAs are cloned from mouse testis. The first strand cDNA was obtained by reverse transcription with P1a1 primer and then amplified and sequenced with P1s and P1a2 to obtain the transcript variants sequence of *Pbx4*. (B) Characterisation of isolated spermatogenic cells by RT‐PCR examination of genes that are differentially expressed.
**Figure S3.** Validation of antibodies used in immunostaining. (A) The expression of PBX4‐FLAG protein in 293 cells was detected by Western blotting using α‐PBX4 and α‐FLAG. (B) Genotyping of WT, homozygous (HOM) and heterozygous (HET) KI mice by PCRs with KI‐F and KI‐ R primers. (C) Sequencing validation of the *Pbx4*×FLAG KI allele. (D) Validation of KI and KO results by using different antibodies in Western blotting assays. (E) Immunohistochemical (IHC) staining of PBX4 in testicular sections using PBX4 antibody. There are a lot of nonspecific signals in KO testicles. Scale bar, 50 μm.
**Figure S4.** Genotyping by sequencing of *Pbx4* KO and *Pbx4*‐KO‐2 mice. (A) Sanger sequencing of the WT and the mutant allele with a 19‐bp deletion. (B) Sanger sequencing of the WT and two mutant alleles in the KO‐2 mice with a 4‐bp an 8‐bp deletion, respectively (KO‐2‐d4 and KO‐2‐d8). The right panel shows the genotyping results of the *Pbx4*‐KO‐2 mice. For KO‐2‐d4, the mutated region was first cloned by PCR (top picture). As the products from WT and KO mice cannot be distinguished by their sizes, they were digested by the BglI restriction enzyme as its site is only introduced in the mutant allele. As a result, the KO PCR product is cut into two smaller pieces (bottom picture). For the KO‐2‐d8 allele, the mutated region was first cloned by PCR (top picture). Similarly, the products from WT and KO mice cannot be distinguished by their sizes, but they can be distinguished by PCRs with a primer pair that specifically amplify the wild‐type allele but not the mutant allele (bottom).
**Figure S5.** Phenotypic evaluation of *Pbx4* KO‐2 mice. (A) Pictures of mice and their testes and epididymis from WT and *Pbx4* KO‐2 mice. (B) Comparison of litter size in WT and KO‐2 mice, *n* ≥ 5. (C) The expression of PBX4 in testicles of WT and KO‐2 mice were identified by Western blotting with α‐PBX4. (D) H&E staining of testicular and epididymis sections in WT and KO‐2 mice.
**Figure S6.** Representative GO terms of biological process categories enriched in up‐and down‐regulated genes of limb development of E11.5 mice hindlimb buds. FDR < 10%.
**Figure S7.** Expression and localisation of PBX4 in mice limbs. (A) PBX4‐FLAG and PRRX1 IF staining of E11.5 mice limb bud section, PBX4 presents nonspecific signal. (B) Expression of *Pbx4* using whole‐mount in situ hybridisation on E11.5 mouse embryos. 3′UTR probe is an RNA probe with a length of 656 nt located upstream of polyA. The corresponding position of MTF#1810 probe is across exons and 3′TUR region. (C) UMAP plot showing the cell types of mouse limb. (D) UMAP plot showing the cell types of mouse limb at each stage. E9.5 means embryonic day 9.5.
**Figure S8.** The expression level of *Pbx4* in mouse tissue at single‐cell resolution (A) Dot plot showing the expression level of *Pbx4* in tissue at different developmental stage of mouse. The expression level is the normalised and logarithmic value of raw counts. (B) UMAP plot showing the cell types of mouse testis (left panel) and expression level of *Pbx4* in mouse testis (right panel). (C) Dot plot showing the expression level of *Pbx4* in cell type of mouse testis. The expression level is standardised between 0 and 1 by gene.
**Figure S9.** Thyroid‐related hormones and the development of rectus femoris in adult KO and WT mice (A) Detection of seral thyroid hormones in adult WT and *Pbx4* KO adult. TSH, thyroid stimulating hormone, FT3, free triiodothyronine, FT4, free thyroxine, T3, Triiodothyronine, T4, thyroxine. (B) HE staining results of rectus femoris of WT and KO mice. Scale bar, 50 μm.

**Video 1.** Morphology and activity of heterozygous (HET) and homozygous (KO) *Pbx4* knockout mice.

**Video 2.** Morphology and activity of normal and disabled *Pbx4* KO mice.

**Table S1.** RNA‐seq results for gene expression in hindlimb bud cells.

**Table S2.** Blood test results for *Pbx4* KO mice.

**Table S3.** ChIP‐seq peaks, related genes and enriched GO BP terms.

**Table S4_1.** De novo motifs enriched in PBX4 ChIP‐seq peaks.

**Table S4_2.** Known motifs enriched in PBX4 ChIP‐seq peaks.

**Table S5.** Sequences of primers and other DNA.

**Table S6.** *Pbx4* KO mice no off‐target effect.

## Data Availability

All data of ChIP‐seq and RNA‐seq have been deposited to Science Data Bank (31253.11.sciencedb.06978) and to the NCBI Gene Expression Omnibus (GSE224371).
