# Supplementary material for: Transcription factor PBX4 regulates limb development and haematopoiesis in mice
Source: Cell Prolif. 2024 Jan 17;57(5):e13580. doi: 10.1111/cpr.13580 (PMC11056705; doi:10.1111/cpr.13580)

Fig. S1

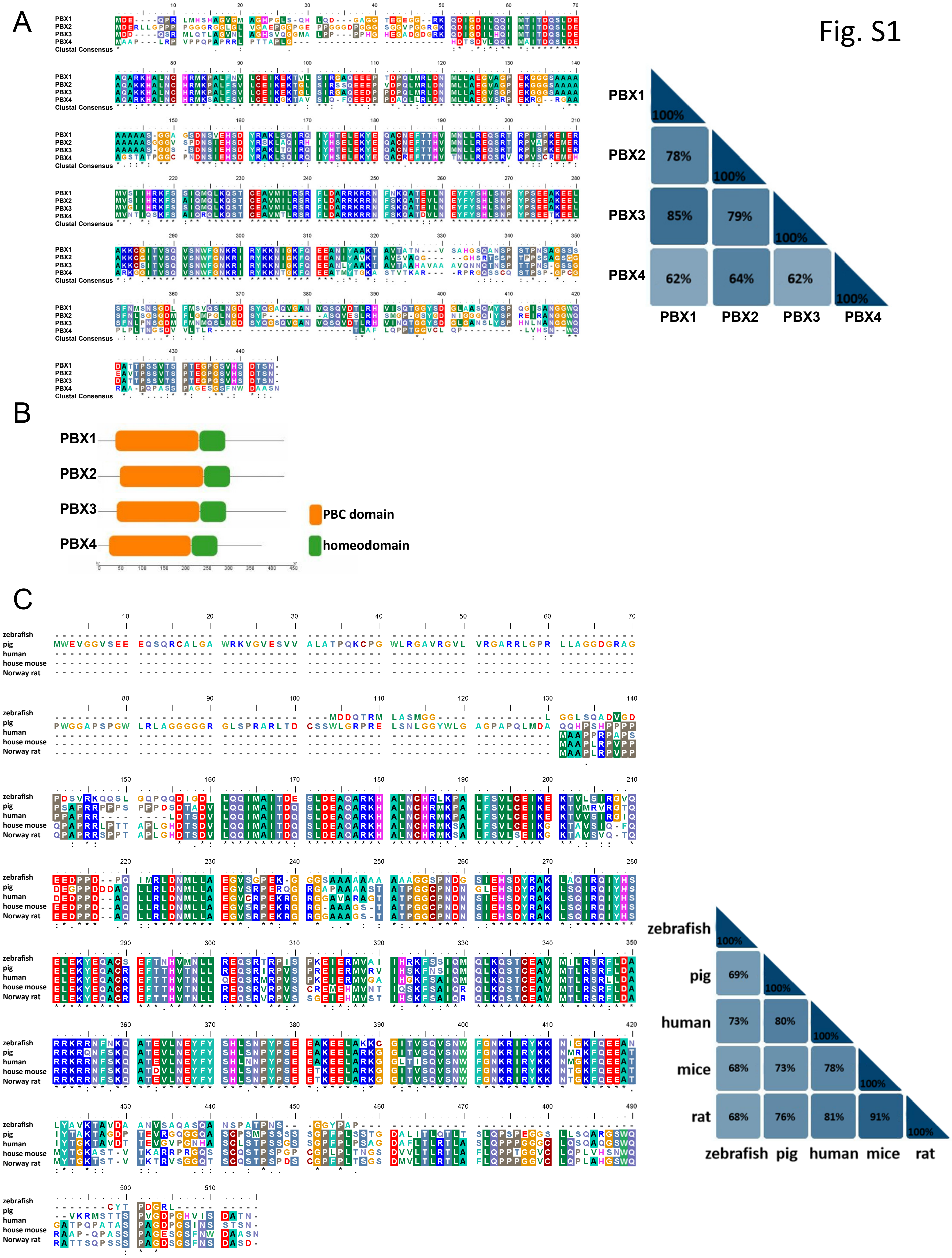
A
PBX1
PBX2
PBX3
PBX4
Clustal Consensus
PBX1
PBX2
PBX3
PBX4
100%
78%
85%
79%
62%
64%
62%
B
PBX1
PBX2
PBX3
PBX4
PBC domain
homeodomain
C
zebrafish
pig
human
house mouse
Norway rat
zebrafish
pig
human
mice
rat
100%
69%
73%
80%
68%
73%
78%
68%
76%
81%
91%

Fig. S2

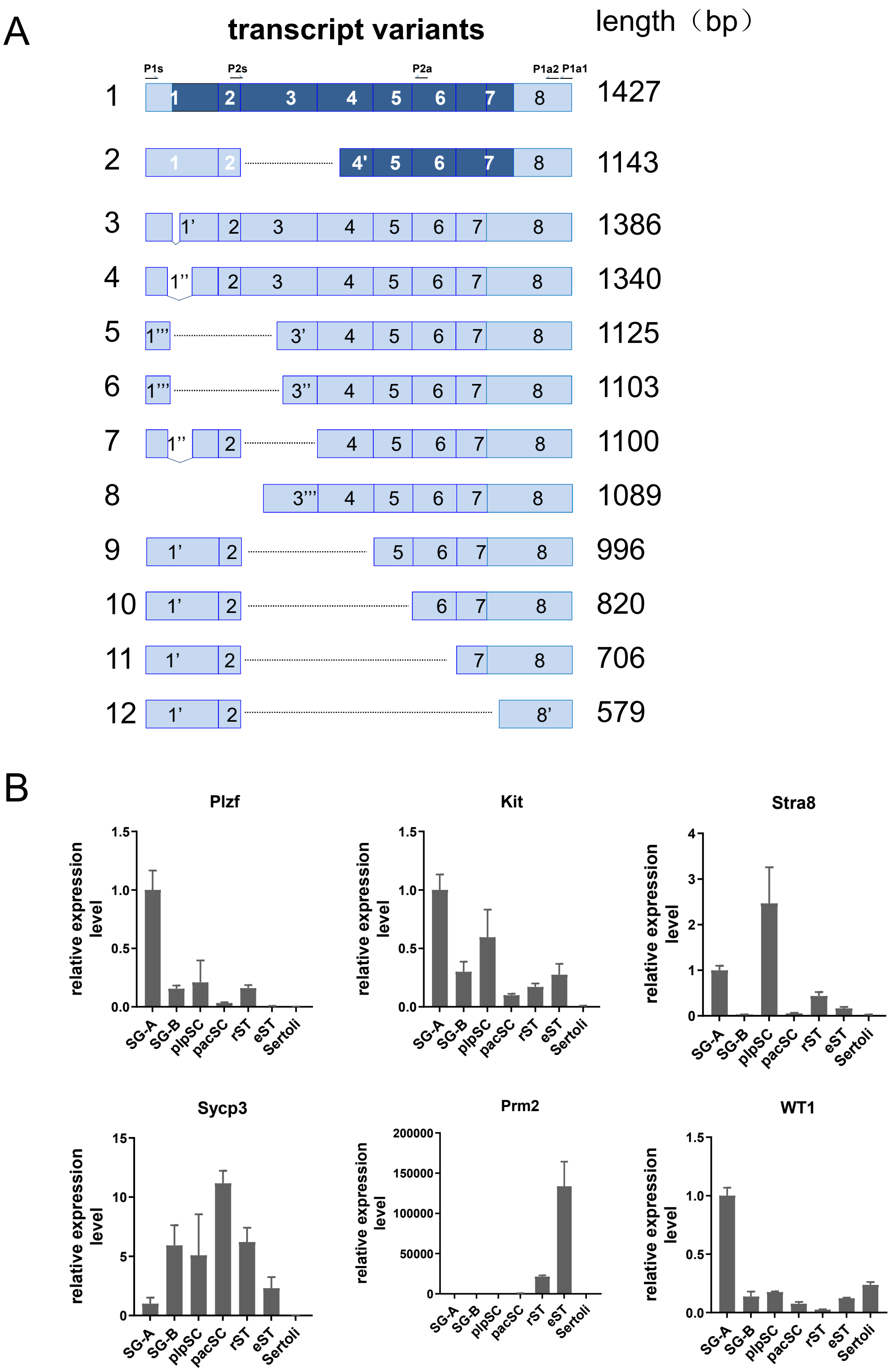
A
transcript variants
length（bp）
P1s
P2s
P2a
P1a2
P1a1
1 1427
2 1143
3 1386
4 1340
5 1125
6 1103
7 1100
8 1089
9 996
10 820
11 706
12 579
B
Plzf
Kit
Stra8
Sycp3
Prm2
WT1
relative expression level
SG-A
SG-B
plpSC
pacSC
rST
eST
Sertoli

Fig. S3

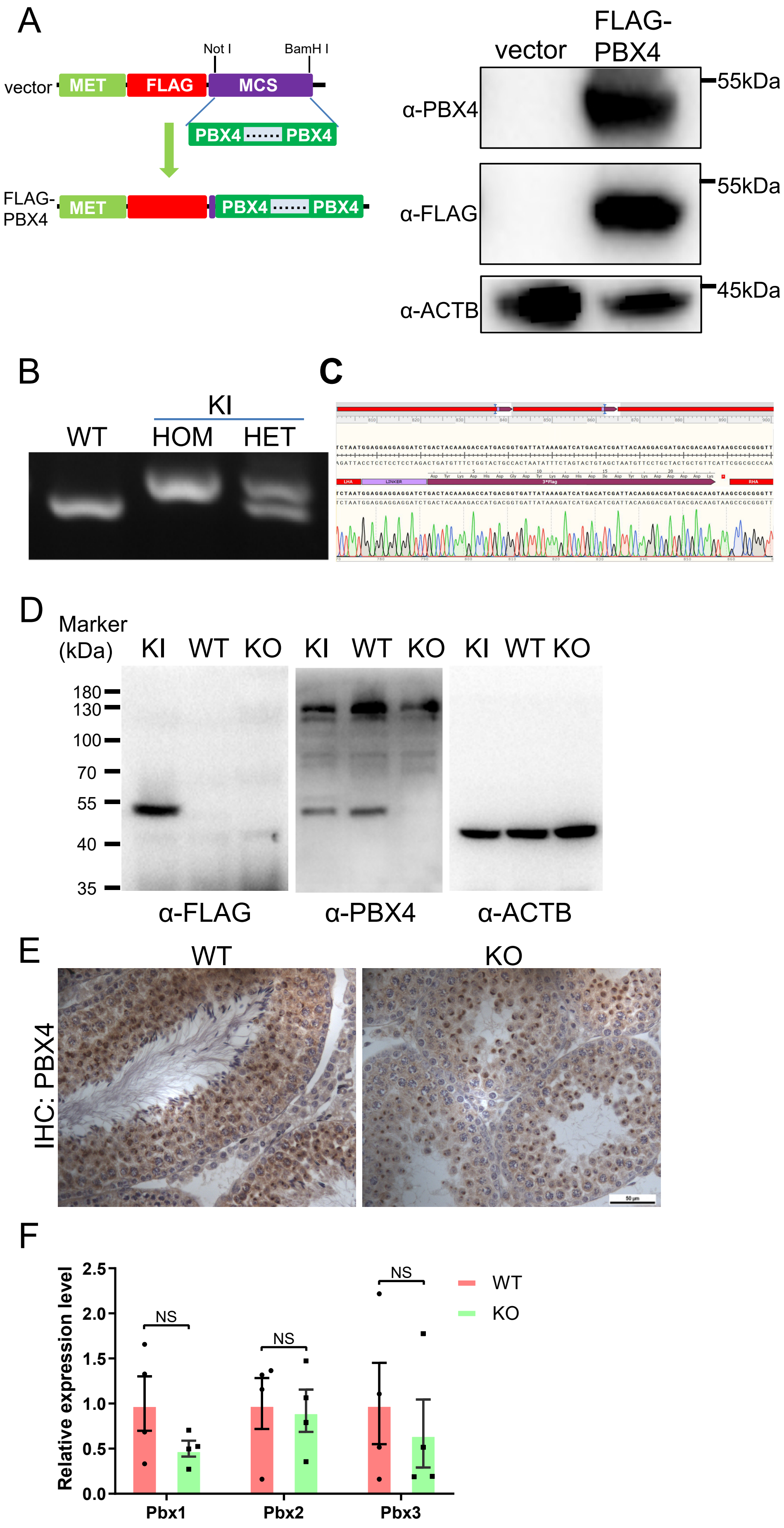
A
vector
MET
FLAG
MCS
Not I
BamH I
PBX4 PBX4
FLAG-PBX4
MET
PBX4 PBX4
vector
FLAG-PBX4
α-PBX4
55kDa
α-FLAG
55kDa
α-ACTB
45kDa
B
KI
WT
HOM
HET
C
D
Marker (kDa)
KI WT KO KI WT KO KI WT KO
180
130
100
70
55
40
35
α-FLAG
α-PBX4
α-ACTB
E
WT
KO
IHC: PBX4
F
Relative expression level
2.5
2.0
1.5
1.0
0.5
0.0
NS
NS
NS
WT
KO
Pbx1
Pbx2
Pbx3

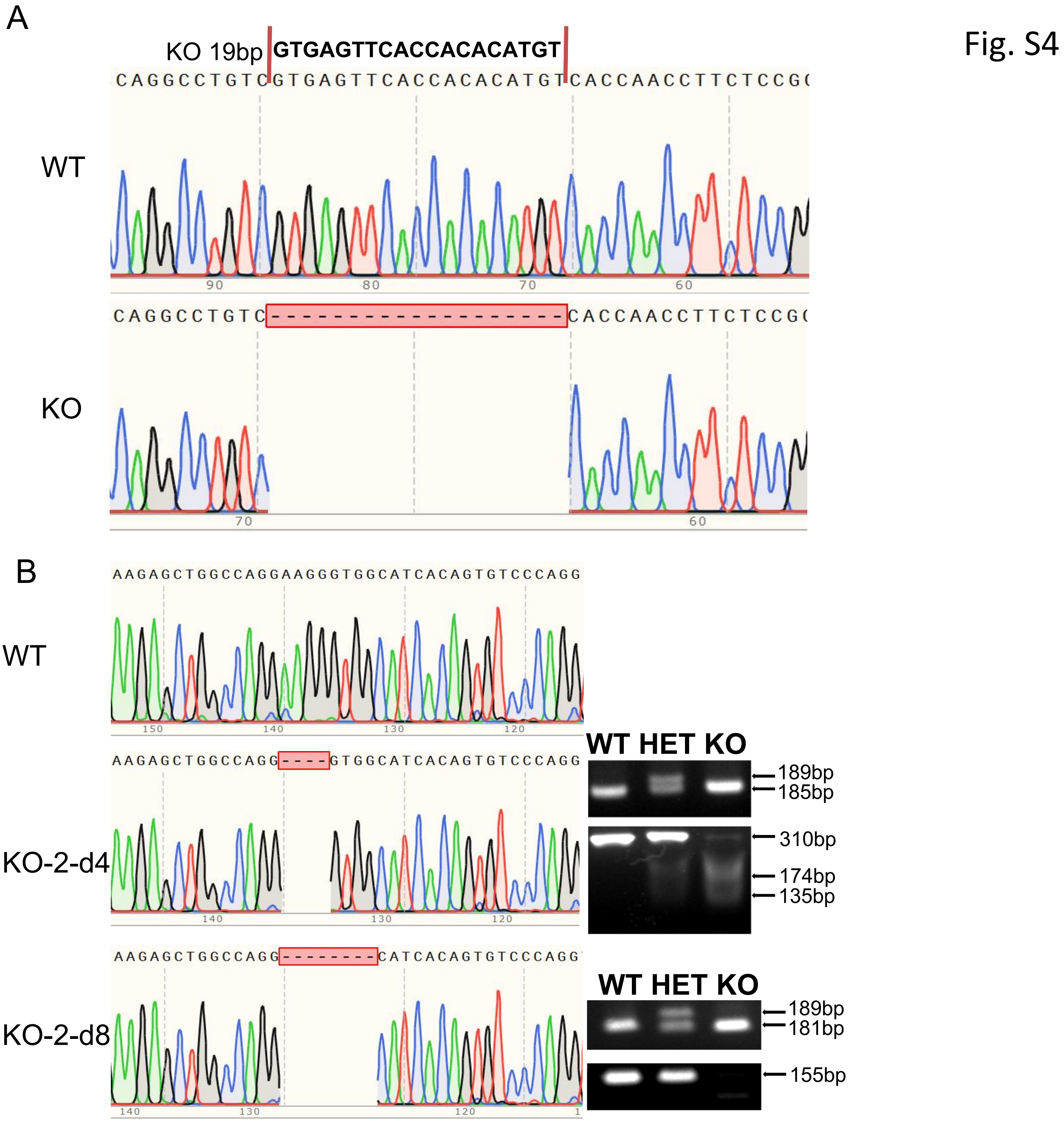
A
Fig. S4
KO 19bp GTGAGTTCACCACACATGT
CAGGCCTGTCGTGAGTTCACCACACATGTCACCAACCTTCTCCG
WT
90
80
70
60
CAGGCCTGTC-------------------CACCAACCTTCTCCG
KO
70
60
B
AAGAGCTGGCCAGGAAGGGTGGCATCACAGTGTCCCAGG
WT
150
140
130
120
AAGAGCTGGCCAGG----GTGGCATCACAGTGTCCCAGG
KO-2-d4
WT HET KO
189bp
185bp
310bp
174bp
135bp
AAGAGCTGGCCAGG--------CATCACAGTGTCCCAGG
KO-2-d8
WT HET KO
189bp
181bp
155bp

Fig. S5

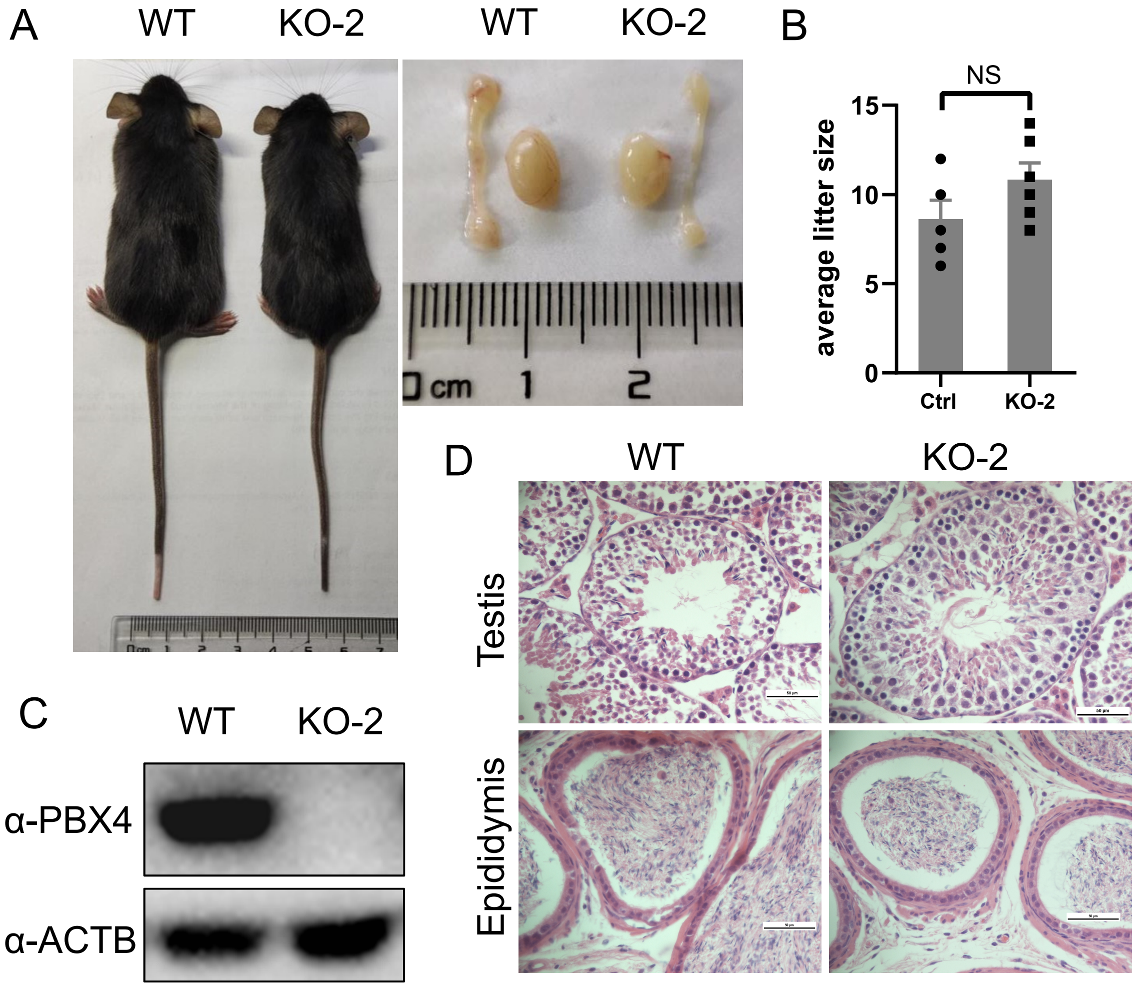
A
WT
KO-2
WT
KO-2
0 cm
1
2
B
NS
average litter size
15
10
5
0
Ctrl
KO-2
C
WT
KO-2
α-PBX4
α-ACTB
D
WT
KO-2
Testis
Epididymis

Fig. S6

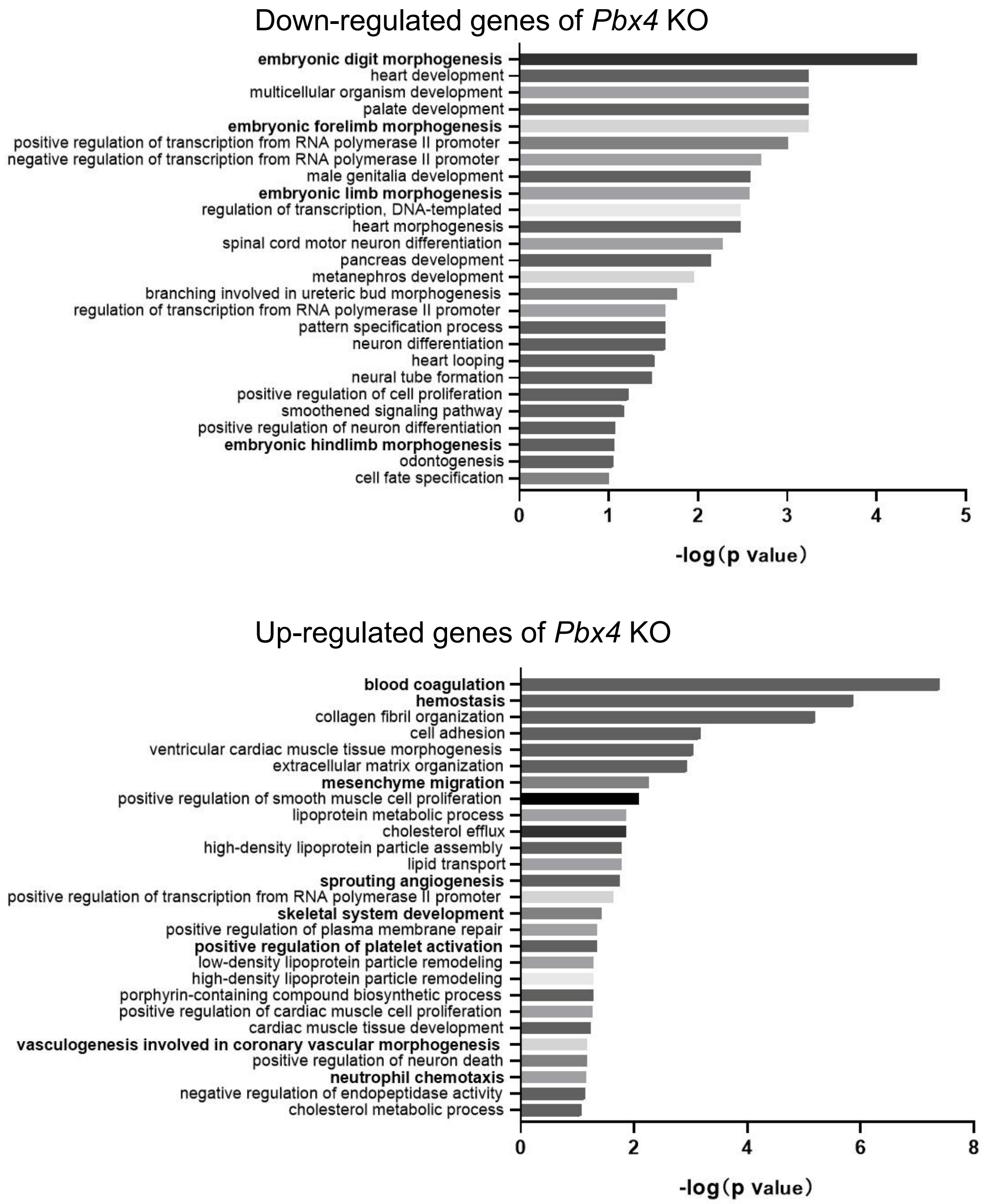
Down-regulated genes of Pbx4 KO
embryonic digit morphogenesis
heart development
multicellular organism development
palate development
embryonic forelimb morphogenesis
positive regulation of transcription from RNA polymerase II promoter
negative regulation of transcription from RNA polymerase II promoter
male genitalia development
embryonic limb morphogenesis
regulation of transcription, DNA-templated
heart morphogenesis
spinal cord motor neuron differentiation
pancreas development
metanephros development
branching involved in ureteric bud morphogenesis
regulation of transcription from RNA polymerase II promoter
pattern specification process
neuron differentiation
heart looping
neural tube formation
positive regulation of cell proliferation
smoothened signaling pathway
positive regulation of neuron differentiation
embryonic hindlimb morphogenesis
odontogenesis
cell fate specification
0 1 2 3 4 5
-log(p value)
Up-regulated genes of Pbx4 KO
blood coagulation
hemostasis
collagen fibril organization
cell adhesion
ventricular cardiac muscle tissue morphogenesis
extracellular matrix organization
mesenchyme migration
positive regulation of smooth muscle cell proliferation
lipoprotein metabolic process
cholesterol efflux
high-density lipoprotein particle assembly
lipid transport
sprouting angiogenesis
positive regulation of transcription from RNA polymerase II promoter
skeletal system development
positive regulation of plasma membrane repair
positive regulation of platelet activation
low-density lipoprotein particle remodeling
high-density lipoprotein particle remodeling
porphyrin-containing compound biosynthetic process
positive regulation of cardiac muscle cell proliferation
cardiac muscle tissue development
vasculogenesis involved in coronary vascular morphogenesis
positive regulation of neuron death
neutrophil chemotaxis
negative regulation of endopeptidase activity
cholesterol metabolic process
0 2 4 6 8
-log(p value)

Fig. S7

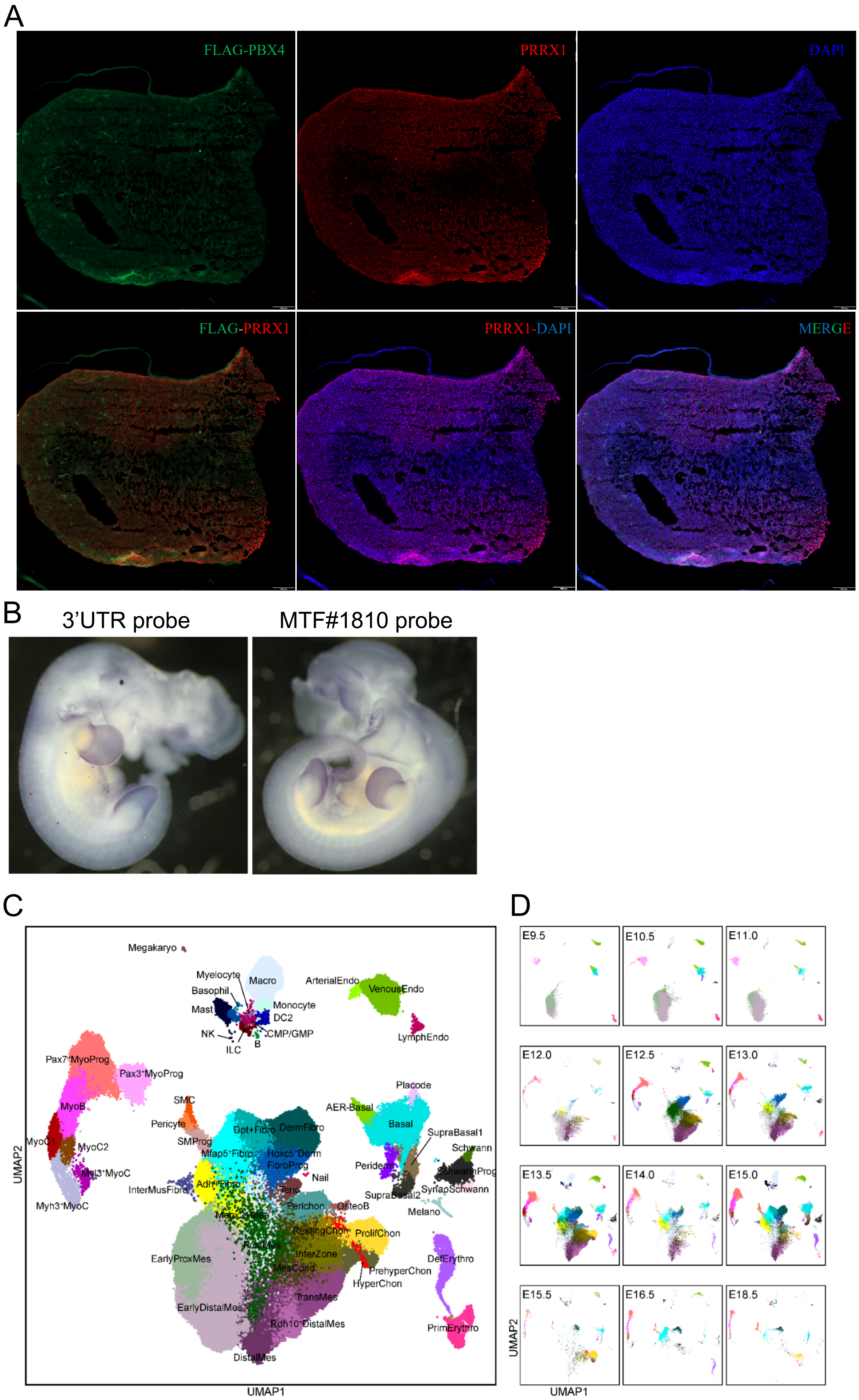
A
FLAG-PBX4
PRRX1
DAPI
FLAG-PRRX1
PRRX1-DAPI
MERGE
B
3'UTR probe
MTF#1810 probe
C
D
UMAP1
UMAP2
E9.5
E10.5
E11.0
E12.0
E12.5
E13.0
E13.5
E14.0
E15.0
E15.5
E16.5
E18.5

Fig. S8

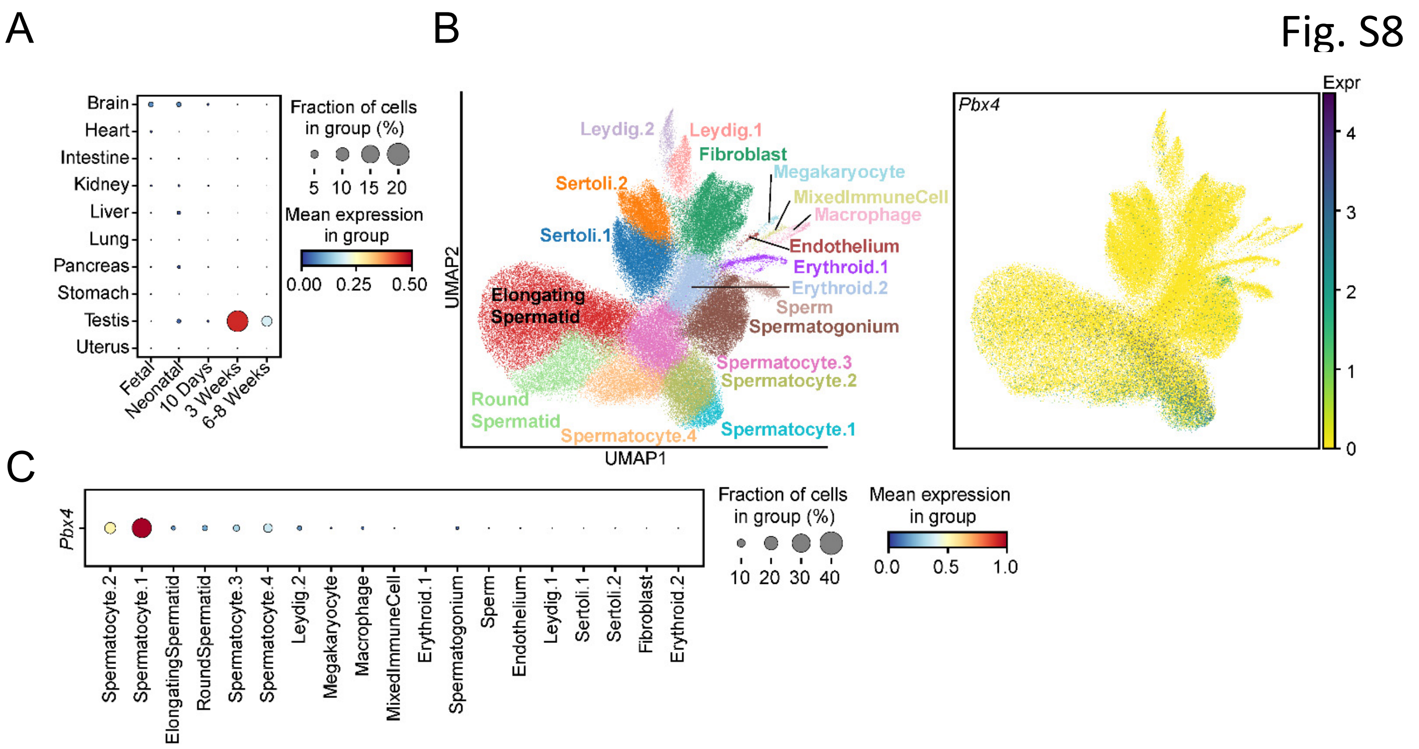
A
Brain
Heart
Intestine
Kidney
Liver
Lung
Pancreas
Stomach
Testis
Uterus
Fetal
Neonatal
10 Days
3 Weeks
6-8 Weeks
Fraction of cells in group (%)
5 10 15 20
Mean expression in group
0.00 0.25 0.50
B
UMAP2
UMAP1
Leydig.2
Leydig.1
Fibroblast
Megakaryocyte
MixedImmuneCell
Macrophage
Sertoli.2
Sertoli.1
Endothelium
Erythroid.1
Erythroid.2
Sperm
Spermatogonium
Elongating Spermatid
Spermatocyte.3
Spermatocyte.2
Round Spermatid
Spermatocyte.4
Spermatocyte.1
Pbx4
Expr
4
3
2
1
0
C
Pbx4
Spermatocyte.2
Spermatocyte.1
ElongatingSpermatid
RoundSpermatid
Spermatocyte.3
Spermatocyte.4
Leydig.2
Megakaryocyte
Macrophage
MixedImmuneCell
Erythroid.1
Spermatogonium
Sperm
Endothelium
Leydig.1
Sertoli.1
Sertoli.2
Fibroblast
Erythroid.2
Fraction of cells in group (%)
10 20 30 40
Mean expression in group
0.0 0.5 1.0

Fig. S9

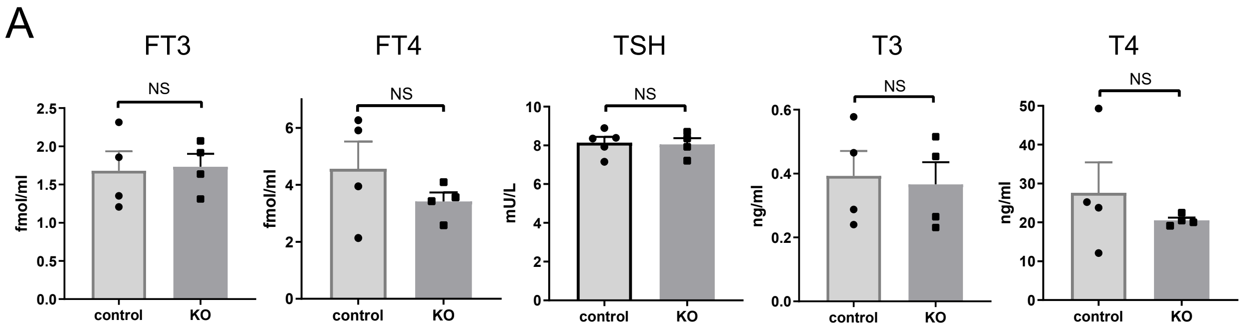

Supplement: Supplementary file 1 — Figure S1. Sequence comparisons of PBC family proteins. (A) PBX1, PBX2, PBX3 and PBX4 alignment (left) and identity (right). (B) PBC family proteins conserved domains. (C) Multi‐alignment analyses and identity of PBX4 orthologues across 5 species. Figure S2. Pbx4 mRNA expression. (A) Structures of alternatively spliced variants of Pbx4, of which cDNAs are cloned from mouse testis. The first strand cDNA was obtained by reverse transcription with P1a1 primer and then amplified and sequenced with P1s and P1a2 to obtain the transcript variants sequence of Pbx4. (B) Characterisation of isolated spermatogenic cells by RT‐PCR examination of genes that are differentially expressed. Figure S3. Validation of antibodies used in immunostaining. (A) The expression of PBX4‐FLAG protein in 293 cells was detected by Western blotting using α‐PBX4 and α‐FLAG. (B) Genotyping of WT, homozygous (HOM) and heterozygous (HET) KI mice by PCRs with KI‐F and KI‐ R primers. (C) Sequencing validation of the Pbx4×FLAG KI allele. (D) Validation of KI and KO results by using different antibodies in Western blotting assays. (E) Immunohistochemical (IHC) staining of PBX4 in testicular sections using PBX4 antibody. There are a lot of nonspecific signals in KO testicles. Scale bar, 50 μm. Figure S4. Genotyping by sequencing of Pbx4 KO and Pbx4‐KO‐2 mice. (A) Sanger sequencing of the WT and the mutant allele with a 19‐bp deletion. (B) Sanger sequencing of the WT and two mutant alleles in the KO‐2 mice with a 4‐bp an 8‐bp deletion, respectively (KO‐2‐d4 and KO‐2‐d8). The right panel shows the genotyping results of the Pbx4‐KO‐2 mice. For KO‐2‐d4, the mutated region was first cloned by PCR (top picture). As the products from WT and KO mice cannot be distinguished by their sizes, they were digested by the BglI restriction enzyme as its site is only introduced in the mutant allele. As a result, the KO PCR product is cut into two smaller pieces (bottom picture). For the KO‐2‐d8 allele, the mutated region wa [file CPR-57-e13580-s006.pdf]
