# Supplementary material for: Transcription factor PBX4 regulates limb development and haematopoiesis in mice
Source: Cell Prolif. 2024 Jan 17;57(5):e13580. doi: 10.1111/cpr.13580 (PMC11056705; doi:10.1111/cpr.13580)
Supplement: Supplementary file 4 — Table S1. RNA‐seq results for gene expression in hindlimb bud cells. [file CPR-57-e13580-s004.docx]

| Term | Count | % | PValue | Genes |
| --- | --- | --- | --- | --- |
| GO:0042733~embryonic digit morphogenesis | 7 | 10.94 | 4.23E-08 | SHH, SALL1, OSR1, HOXD13, HOXD12, TBX3, TBX2 |
| GO:0007507~heart development | 9 | 14.06 | 2.41E-06 | EDNRA, SHH, SALL1, ALDH1A2, OSR1, ID3, ISL1, SOX4, STRA6 |
| GO:0007275~multicellular organism development | 15 | 23.44 | 2.43E-06 | FOXA1, SEMA5B, ZFP521, HOXD13, UNC5C, HOXD12, HOXA13, ISL1, TBX3, TBX2, CLGN, SHH, SIX2, MGP, ID3 |
| GO:0060021~palate development | 6 | 9.38 | 3.93E-06 | SHH, FOXF2, OSR1, TCF21, TBX3, TBX2 |
| GO:0035115~embryonic forelimb morphogenesis | 5 | 7.81 | 4.08E-06 | SHH, ALDH1A2, OSR1, HOXA13, TBX3 |
| GO:0045944~positive regulation of transcription from RNA polymerase II promoter | 15 | 23.44 | 8.16E-06 | FOXA1, ZFP521, FOXF2, OSR1, TCF21, HOXD13, HOXA13, ISL1, TBX3, TBX2, SHH, SALL1, SIX2, MZF1, SOX4 |
| GO:0000122~negative regulation of transcription from RNA polymerase II promoter | 13 | 20.31 | 2.11E-05 | TLE4, FOXA1, PTCH1, OSR1, TCF21, ISL1, TBX3, TBX2, SHH, SALL1, ID3, MZF1, NKX3-2 |
| GO:0030539~male genitalia development | 4 | 6.25 | 3.06E-05 | SHH, HOXD13, HOXA13, TBX3 |
| GO:0030326~embryonic limb morphogenesis | 5 | 7.81 | 3.48E-05 | SHH, ALDH1A2, PTCH1, HOXD13, GDF5 |
| GO:0006355~regulation of transcription, DNA-templated | 14 | 21.88 | 5.52E-05 | TLE4, FOXA1, FOXF2, HOXD13, HOXD12, HOXA13, ISL1, TBX3, TBX2, SHH, SIX2, MZF1, SOX4, NKX3-2 |
| GO:0003007~heart morphogenesis | 5 | 7.81 | 5.89E-05 | ALDH1A2, PTCH1, ISL1, TBX3, TBX2 |
| GO:0021522~spinal cord motor neuron differentiation | 4 | 6.25 | 1.01E-04 | SHH, PTCH1, ISL1, SOX4 |
| GO:0031016~pancreas development | 4 | 6.25 | 1.46E-04 | SHH, ALDH1A2, ISL1, NKX3-2 |
| GO:0001656~metanephros development | 4 | 6.25 | 2.53E-04 | SHH, SIX2, OSR1, ID3 |
| Term | Count | % | PValue | Genes |
| GO:0001658~branching involved in ureteric bud morphogenesis | 4 | 6.25 | 4.50E-04 | SHH, SALL1, PTCH1, TCF21 |
| GO:0006357~regulation of transcription from RNA polymerase II promoter | 14 | 21.88 | 6.43E-04 | FOXA1, FOXF2, OSR1, TCF21, HOXD13, HOXA13, ISL1, TBX3, TBX2, LBX2, SALL1, SIX2, NKX3-2, ZFP771 |
| GO:0007389~pattern specification process | 4 | 6.25 | 6.91E-04 | SHH, PTCH1, HOXD13, HOXD12 |
| GO:0030182~neuron differentiation | 6 | 9.38 | 7.50E-04 | FOXA1, SHH, SALL1, ALDH1A2, ID3, ISL1 |
| GO:0001947~heart looping | 4 | 6.25 | 0.00109 | SHH, ALDH1A2, TBX3, TBX2 |
| GO:0001841~neural tube formation | 3 | 4.69 | 0.00125 | SHH, PTCH1, SOX4 |
| GO:0008284~positive regulation of cell proliferation | 8 | 12.50 | 0.00263 | GADD45GIP1, EDNRA, SHH, ALDH1A2, ISL1, SOX4, TBX3, TBX2 |
| GO:0007224~smoothened signaling pathway | 4 | 6.25 | 0.00325 | FOXA1, SHH, SALL1, PTCH1 |
| GO:0045666~positive regulation of neuron differentiation | 4 | 6.25 | 0.00445 | FOXA1, SHH, SALL1, GDF5 |
| GO:0035116~embryonic hindlimb morphogenesis | 3 | 4.69 | 0.00472 | SHH, OSR1, TBX3 |
| GO:0042476~odontogenesis | 3 | 4.69 | 0.00499 | SHH, OSR1, ID3 |
| GO:0001708~cell fate specification | 3 | 4.69 | 0.00583 | SHH, TBX3, TBX2 |
