# Supplementary material for: Transcription factor PBX4 regulates limb development and haematopoiesis in mice
Source: Cell Prolif. 2024 Jan 17;57(5):e13580. doi: 10.1111/cpr.13580 (PMC11056705; doi:10.1111/cpr.13580)
Supplement: Supplementary file 5 — Table S2. Blood test results for Pbx4 KO mice. [file CPR-57-e13580-s005.docx]

| 12-week mice | HET | | KO | |  |
| --- | --- | --- | --- | --- | --- |
|  | Mean | SEM | Mean | SEM | P value |
| White blood cells, WBC （ *10^9/L ） | 15.60 | 3.15 | 12.68 | 1.78 | 0.443 |
| Monocyte percentage, Mon% （ % ） | 0.60 | 0.26 | 0.84 | 0.26 | 0.530 |
| Percentage of eosinophils, Eos% （ % ） | 0.18 | 0.06 | 0.30 | 0.05 | 0.172 |
| Percentage of basophil, Bas% （ % ） | 0.34 | 0.04 | 0.26 | 0.02 | 0.126 |
| Red blood cell, RBC(*10^12/L) | 10.82 | 0.28 | 11.48 | 0.29 | 0.138 |
| Mean red blood cell volume, MCV （ fL ） | 50.72 | 0.40 | 51.28 | 0.38 | 0.336 |
| Mean erythrocyte hemoglobin content, MCH （ pg ） | 15.72 | 0.55 | 14.78 | 0.33 | 0.180 |
| Mean erythrocyte hemoglobin concentration, MCHC （ g/L ） | 293.00 | 4.17 | 287.20 | 2.15 | 0.252 |
| Coefficient of variation of erythrocyte distribution width, RDW-CV （ % ） | 22.62 | 1.18 | 23.58 | 1.10 | 0.568 |
| Standard deviation of erythrocyte distribution width, RDW-SD （ fL ） | 26.72 | 0.92 | 26.64 | 0.97 | 0.954 |
| Platelet count, PLT （ *10^9/L ） | 1179.20 | 102.26 | 1201.00 | 72.01 | 0.866 |
| Mean platelet volume, MPV （ fL ） | 8.16 | 0.15 | 8.04 | 0.31 | 0.738 |
| Platelet distribution width, PDW （ fL ） | 9.74 | 0.21 | 9.48 | 0.38 | 0.568 |
| plateletcrit, PCT （ % ） | 1.16 | 0.13 | 1.15 | 0.11 | 0.927 |
| platelet -larger cell ratio, P-LCR （ % ） | 12.32 | 1.26 | 9.84 | 0.44 | 0.100 |

| 5-week mice | HET | | KO | |  |
| --- | --- | --- | --- | --- | --- |
|  | Mean | SEM | Mean | SEM | P value |
| White blood cells, WBC （ *10^9/L ） | 5.78 | 0.66 | 6.58 | 0.93 | 0.501 |
| Monocyte percentage, Mon% （ % ） | 5.26 | 0.14 | 5.64 | 0.23 | 0.195 |
| Percentage of eosinophils, Eos% （ % ） | 2.60 | 0.38 | 2.72 | 0.42 | 0.836 |
| Percentage of basophil, Bas% （ % ） | 0.80 | 0.15 | 0.82 | 0.07 | 0.908 |
| Red blood cell, RBC(*10^12/L) | 8.04 | 0.37 | 8.57 | 0.21 | 0.245 |
| Mean red blood cell volume, MCV （ fL ） | 61.28 | 1.33 | 62.40 | 0.96 | 0.514 |
| Mean erythrocyte hemoglobin content, MCH （ pg ） | 15.56 | 0.49 | 15.92 | 0.33 | 0.559 |
| Mean erythrocyte hemoglobin concentration, MCHC （ g/L ） | 253.60 | 3.31 | 255.00 | 1.87 | 0.722 |
| Coefficient of variation of erythrocyte distribution width, RDW-CV （ % ） | 20.12 | 0.89 | 20.42 | 0.74 | 0.802 |
| Standard deviation of erythrocyte distribution width, RDW-SD （ fL ） | 37.26 | 2.14 | 37.94 | 0.96 | 0.780 |
| Platelet count, PLT （ *10^9/L ） | 915.80 | 98.74 | 958.60 | 33.33 | 0.692 |
| Mean platelet volume, MPV （ fL ） | 7.78 | 0.32 | 7.84 | 0.29 | 0.893 |
| Platelet distribution width, PDW （ fL ） | 8.36 | 0.67 | 9.28 | 0.83 | 0.413 |
| plateletcrit, PCT （ % ） | 0.70 | 0.06 | 0.75 | 0.03 | 0.486 |
| platelet -larger cell ratio, P-LCR （ % ） | 11.44 | 2.57 | 12.22 | 2.44 | 0.831 |
