# Supplementary material for: Transcription factor PBX4 regulates limb development and haematopoiesis in mice
Source: Cell Prolif. 2024 Jan 17;57(5):e13580. doi: 10.1111/cpr.13580 (PMC11056705; doi:10.1111/cpr.13580)
Supplement: Supplementary file 6 — Table S3. ChIP‐seq peaks, related genes and enriched GO BP terms. [file CPR-57-e13580-s009.docx]

| Term | Count | % | PValue | -LOG10  (PValue) | Genes |
| --- | --- | --- | --- | --- | --- |
| regulation of transcription from RNA polymerase II promoter | 179 | 13.69 | 4.61E-21 | 20.34 | GM14124, ZFP446, ATF2, ZFP963, ZFP961, ZFP967, ZFP966, UBP1, RORB, AW146154, IKZF3, ZFP119A, NR3C2, ZFP119B, HMGN5, ZFP281, KAT5, ZMIZ2, RUVBL2, CREB3L2, RUVBL1, KMT5A, SOX9, CCNL2, PKNOX1, HMGN2, ZFP960, 2010315B03RIK, HMGN3, SOX7, ZFP39, HMGN1, ZFP975, ZFP973, GM7072, GM14139, ZFP976, RFX2, ARID5A, ZBTB32, RFX3, RFX1, THAP1, RUNX1, MED7, MYCL, ZFP46, ZFP174, ZFP691, ATF7, ZFP971, ZFP574, HDGFL2, CGGBP1, ZFP345, ZFP869, CTBP2, ZFP868, GLO1, ZFP866, GATA2, FOXO3, ZBTB5, FOXO1, MBIP, CAMTA1, E4F1, OTX1, ZBTB7A, RREB1, ZSCAN18, BHLHA9, ZBTB18, SMAD2, TFAP2C, GM14430, POU2F1, ZFP994, ZFP873, BRPF3, ZFP998, ZFP239, PBX3, NFATC3, 2610008E11RIK, MAFA, TRERF1, SMARCA2, MLXIPL, ZFP68, NR4A3, SP2, SP1, SP4, RFXAP, SP3, CREBRF, HBP1, MXD1, SIM1, RCOR1, NFE2L1, ZFP991, MXD4, EZH2, GM14322, CSRNP2, GM5141, ZFP367, PHF20, GM14325, HNF4G, ZFP809, ZFP808, DMBX1, HEY1, ZIC3, DMRTB1, SIX3, ZFP120, CIC, ZFP931, ZFP930, ZFP655, ZFP935, PEG3, ZFP934, ZFP932, TCF12, ZFP938, OLIG2, ARID1B, ETV5, GM14295, ETV6, TOX2, GM10778, LCORL, KDM7A, ZFP788, RBPJL, AU041133, CEBPG, FOXK2, MLLT1, ZBTB45, NR2C2, ZFP97, E2F1, HES1, MEF2D, ZFP541, RXRG, ZFP386, STOX1, ZFP143, MBD4, IRX2, ZFP951, ZFP950, ZFP433, ZFP438, GM15446, USP22, ZFP959, DMRT3, ZFP958, POU4F1, HINFP, AI987944, GM3604, KLF5, NFIA, NFIC, GM6710, FOSB, TAF4, ZFP157, GM45871 |
| negative regulation of transcription from RNA polymerase II promoter | 99 | 7.57 | 2.23E-09 | 8.65 | TCERG1, ATF2, ZFP961, ENO1, ZFP281, KAT5, KMT5A, SOX9, HMGN2, TLE4, ARID5A, ZBTB32, DICER1, TLE6, THAP1, RUNX1, ZFP46, ZFP174, ATF7, ZFP971, CGGBP1, GATA2, FOXO3, ZBTB5, SCRT2, E4F1, SUDS3, ZBTB7A, RREB1, ZBTB18, CBX8, HEXIM2, WFS1, ZFP239, 2610008E11RIK, COQ7, MLXIPL, NR4A3, SP2, ID2, CREBRF, PAF1, HBP1, MXD1, NFE2L1, FGFR2, PHF19, MXD4, EZH2, GM14322, TCF25, CHD8, GM14325, YBX1, DMBX1, XPO1, HEY1, SIN3B, SIN3A, SUFU, ZMYM5, DNMT3B, ZFP120, CIC, JARID2, NCOA2, RBM15, PEG3, ZFP932, SIRT6, PEX2, OLIG2, ETV5, ETV6, SFPQ, CRY1, STRAP, HDAC4, AU041133, LIN37, UHRF1, NR2C2, DNAJB5, E2F1, HES1, SUZ12, IRX2, HNRNPAB, POU4F1, HINFP, GATAD2A, AI987944, PER2, KLF5, PER3, NFIC, GM6710, ZFP157, GM45871 |
| positive regulation of transcription from RNA polymerase II promoter | 116 | 8.87 | 3.02E-09 | 8.52 | TCERG1, ATF2, PRDM9, MAML1, UBP1, RORB, IKZF3, RPS6KA5, KAT5, ZMIZ2, RUVBL2, CREB3L2, NFATC2IP, KAT7, SOX9, PKNOX1, HMGN3, CDK5RAP3, RFX2, ARID5A, RFX3, DICER1, RUNX1, KAT6B, PRKD2, ATF7, CASZ1, AKNA, CTBP2, GATA2, FOXO3, FOXO1, HLTF, CAMTA1, E4F1, SMYD3, OTX1, RREB1, BCL9L, ZBTB18, SMAD2, TFAP2C, POU2F1, JUP, SS18L2, PBX3, NFATC3, MAFA, COQ7, TRERF1, SMARCA2, MLXIPL, NR4A3, SP1, RFXAP, SP3, CREBRF, HNRNPD, PIN1, ATM, NFE2L1, FGFR2, GLP1R, TOP2A, CSRNP2, SETD3, CHD8, AKAP8L, HNF4G, YBX1, PTBP1, HEY1, SIN3A, ZIC3, SIX3, NCK1, NCOA2, ACTR2, BCAS3, PEG3, TCF12, BAZ1B, ETV5, ETV6, TOX2, SFPQ, PTMA, HDAC4, RBPJL, CEBPG, FOXK2, NR2C2, RNF4, PSMC3IP, MAPK7, E2F1, HES1, TRP53BP1, MEF2D, RXRG, STOX1, ZFP143, RTRAF, CDKN2A, POU4F1, HINFP, KLF5, NFIA, NFIC, TCEA1, FOSB, CRLF3, SSBP2, SSBP3, RBMX, SSBP4 |
| chromatin organization | 47 | 3.59 | 4.11E-09 | 8.39 | HDAC4, N6AMT1, PRDM9, UHRF1, PHF20, CHD8, CHD6, HMGN5, ING3, KAT5, RUVBL2, HLTF, RUVBL1, KAT7, SUDS3, SMYD3, KMT5A, JARID2, HMGN2, ZBTB7A, HMGN3, HMGN1, KDM6A, SUZ12, RNF20, CBX8, KDM4A, H2AZ2, KDM4B, USP49, 0610010K14RIK, BRPF3, KMT5C, USP22, DAPK3, BAZ1B, SMARCA2, ARID1B, KANSL1, KAT6B, KANSL2, ANP32E, RCCD1, RCOR1, KDM7A, PHF19, EZH2 |
| regulation of transcription, DNA-templated | 116 | 8.87 | 7.88E-09 | 8.10 | ATF2, PRDM9, ZFP967, KEAP1, RORB, NR3C2, RPS6KA5, KAT5, KHSRP, RUVBL2, CREB3L2, RUVBL1, KAT7, SOX9, PKNOX1, SOX7, ZFP39, TLE4, TLE3, ZFP973, ZFP977, RFX2, RFX3, RFX1, TLE6, THAP1, RUNX1, MYCL, ZFP46, KAT6B, ATF7, LRIF1, CASZ1, GM14305, ZFP866, GATA2, FOXO3, NLK, FOXO1, SCRT2, MBIP, E4F1, OTX1, ZBTB7A, SMAD2, TFAP2C, POU2F1, MTERF2, PBX3, NFATC3, FOXN3, MAFA, FOXN2, SMARCA2, NR4A3, SP1, CNOT3, SP3, CREBRF, HBP1, SIM1, NFE2L1, PHF19, GM21814, GM14288, GM14326, GM14327, PHF20, ZFP768, 6720489N17RIK, HNF4G, YBX1, ZFP809, DMBX1, ING3, HEY1, SIN3B, SIN3A, DMRTB1, SIX3, ZFP120, NCOA2, ZFP932, ZFP936, OLIG2, ETV5, ETV6, SFPQ, SETBP1, GM10778, RBPJL, TGFBRAP1, FOXK2, MLLT1, NR2C2, SPTY2D1, GM38396, E2F1, HES1, TRP53BP1, MEF2D, RXRG, ZFP781, RNF20, IRX2, GM10033, DMRT3, POU4F1, HINFP, 3300002I08RIK, KLF5, NFIA, NFIC, EU599041, TCEA1, FOSB |
| positive regulation of transcription, DNA-templated | 70 | 5.35 | 2.15E-06 | 5.67 | ZFP326, FANK1, SETD3, PHF20, CHD8, HNF4G, RORB, ING3, ZFP281, KAT5, ZIC3, RUVBL2, CREB3L2, RUVBL1, SOX9, PKNOX1, SOX7, TCF12, DYRK1A, RFX3, ARID1B, ETV5, RUNX1, ASPH, KAT6B, KDM7A, RBPJL, HDAC4, CASZ1, FOXK2, FOXO3, PRDM11, RNF4, FOXO1, MAPK9, CAND1, MED30, SERTAD2, E2F1, HES1, MEF2D, TRIM45, RNF20, SMAD2, FZD4, CDKN2A, USP22, NFATC3, MAFA, HNRNPAB, TRERF1, SMARCA2, HINFP, GATAD2A, MLXIPL, KLF5, NR4A3, PHF5A, KANSL1, NFIA, SP1, KANSL2, ID2, RFXAP, SP3, TCEA1, TAF4, CRLF3, SSBP3, RBMX |
| in utero embryonic development | 42 | 3.21 | 2.49E-06 | 5.60 | ITGB1, ATF2, MIR17, CHD8, AMD1, KEAP1, SMG9, YBX1, ENO1, SIN3A, RDH10, HES1, RBBP6, PLCG1, SLC39A1, MYH10, PSPH, SLC39A3, KDM6A, ZBTB18, SMAD2, NLRP4F, STIL, PRMT1, SLC30A1, UBE2A, COQ7, PRRC2B, HINFP, BYSL, RUNX1, GATAD2A, COPS3, SP2, SP1, SP3, ERCC2, NMT1, MYH9, ATF7, FGFR2, BCL2L1 |
| negative regulation of transcription, DNA-templated | 66 | 5.05 | 2.77E-06 | 5.56 | CHD8, RORB, ENO1, DMBX1, SCML2, RPS6KA5, ZFP281, KAT5, HEY1, SIN3B, SIN3A, SIX3, DNMT3B, KAT7, SOX9, KMT5A, CIC, JARID2, SOX7, TLE4, NCOA2, RFX3, SIRT6, NRG1, RUNX1, SFPQ, KAT6B, CRY1, ZFP174, HDAC4, SET, ZFP869, CTBP2, FOXK2, PRDM11, E2F1, HES1, SUDS3, RREB1, ZFP541, ZBTB7A, ZBTB18, SMAD2, KDM4A, HEXIM2, HSPA8, POU2F1, CDKN2A, ZFP438, FOXN3, TRERF1, SMARCA2, HINFP, GATAD2A, HNRNPL, PER2, MLXIPL, CENPF, ID2, NFIC, SP3, NOSTRIN, MPHOSPH8, RCOR1, MXD4, EZH2 |
| mRNA processing | 41 | 3.13 | 1.21E-05 | 4.92 | RBM27, ZFP326, TCERG1, AKAP8L, CELF6, YBX1, TSEN2, HNRNPLL, PRPF8, PNN, PTBP1, RBM4, ALKBH5, PABPN1, KHSRP, RBBP6, HNRNPA1, PRKACA, CMTR2, HSPA8, SRRM4, PRPF38A, USP49, PRPF40A, WBP11, LSM4, HNRNPL, PAN2, KIN, XAB2, SFPQ, DDX39A, PHF5A, NOVA1, RPUSD3, STRAP, SRSF4, HNRNPC, SRSF6, CACTIN, RBMX |
| DNA repair | 43 | 3.29 | 1.32E-05 | 4.88 | SWSAP1, MDC1, VCP, FBH1, UHRF1, FAAP24, PDS5B, HPF1, KAT5, RNF138, RUVBL2, SWI5, HLTF, RUVBL1, KAT7, TRP53BP1, ZBTB7A, RFC5, ZRANB3, UVRAG, MBD4, SLF2, FANCL, ERCC6L2, KMT5C, UBE2A, MLH1, INIP, HINFP, FANCF, RBX1, KIN, XAB2, SFPQ, RPA3, ERCC1, SHLD1, MSH5, ERCC2, SPRTN, ATM, HDGFL2, CUL4B |
