# Supplementary material for: Transcription factor PBX4 regulates limb development and haematopoiesis in mice
Source: Cell Prolif. 2024 Jan 17;57(5):e13580. doi: 10.1111/cpr.13580 (PMC11056705; doi:10.1111/cpr.13580)
Supplement: Supplementary file 7 — Table S4_1. De novo motifs enriched in PBX4 ChIP‐seq peaks. [file CPR-57-e13580-s010.pdf]

Homer *de novo* Motif Results (homer/Flag.vs.input/)

Known Motif Enrichment Results
Gene Ontology Enrichment Results
If Homer is having trouble matching a motif to a known motif, try copy/pasting the matrix file into STAMP
More information on motif finding results: HOMER | Description of Results | Tips
Total target sequences = 1338
Total background sequences = 41819
* - possible false positive

Rank	Motif	P-value	log P-pvalue	% of Targets	% of Background	STD(Bg STD)	Best Match/Details	Motif File
1	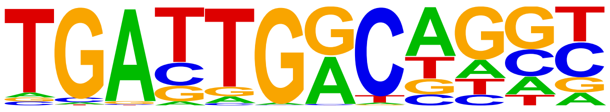	1e-708	-1.631e+03	59.42%	4.27%	41.4bp (67.3bp)	Pknox1(Homeobox)/ES-Prep1-ChIP-Seq(GSE63282)/Homer(0.960) More Information | Similar Motifs Found	motif file (matrix)
2	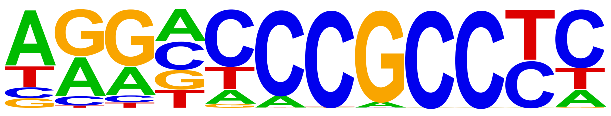	1e-280	-6.447e+02	54.63%	13.27%	53.1bp (75.1bp)	Sp2(Zf)/HEK293-Sp2.eGFP-ChIP-Seq(Encode)/Homer(0.899) More Information | Similar Motifs Found	motif file (matrix)
3	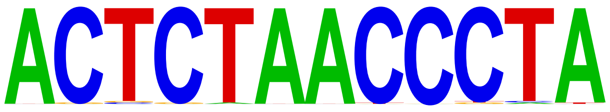	1e-99	-2.292e+02	3.29%	0.01%	54.1bp (51.4bp)	Spz1/MA0111.1/Jaspar(0.643) More Information | Similar Motifs Found	motif file (matrix)
4	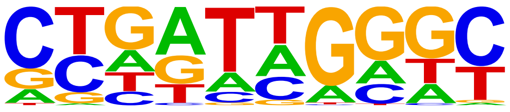	1e-92	-2.124e+02	14.20%	2.14%	52.4bp (62.8bp)	NFYA/MA0060.3/Jaspar(0.747) More Information | Similar Motifs Found	motif file (matrix)
5	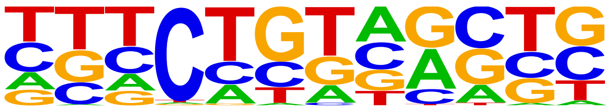	1e-57	-1.330e+02	1.94%	0.01%	56.9bp (32.8bp)	POL009.1_DCE_S_II/Jaspar(0.689) More Information | Similar Motifs Found	motif file (matrix)
6	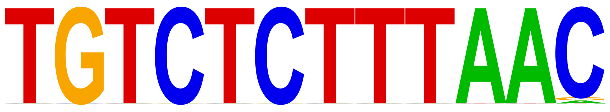	1e-54	-1.244e+02	1.64%	0.00%	76.6bp (22.1bp)	FOXE1/MA1487.1/Jaspar(0.666) More Information | Similar Motifs Found	motif file (matrix)
7	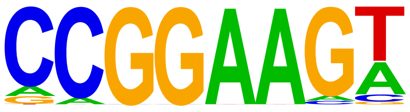	1e-53	-1.236e+02	18.83%	6.29%	56.6bp (79.5bp)	ELF1(ETS)/Jurkat-ELF1-ChIP-Seq(SRA014231)/Homer(0.975) More Information | Similar Motifs Found	motif file (matrix)
8	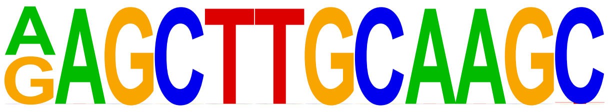	1e-48	-1.113e+02	1.49%	0.00%	47.6bp (9.0bp)	Nr2e3/MA0164.1/Jaspar(0.684) More Information | Similar Motifs Found	motif file (matrix)
9	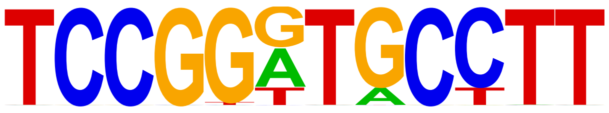	1e-48	-1.113e+02	1.49%	0.00%	58.2bp (42.4bp)	PB0077.1_Spdef_1/Jaspar(0.696) More Information | Similar Motifs Found	motif file (matrix)
10	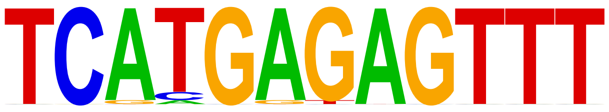	1e-45	-1.049e+02	1.42%	0.00%	69.8bp (25.0bp)	Nkx2-5(var.2)/MA0503.1/Jaspar(0.608) More Information | Similar Motifs Found	motif file (matrix)
11	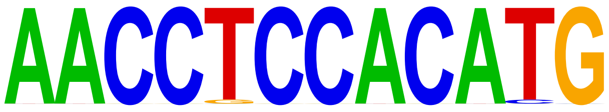	1e-38	-8.944e+01	1.49%	0.01%	16.6bp (20.0bp)	MXI1/MA1108.2/Jaspar(0.704) More Information | Similar Motifs Found	motif file (matrix)
12	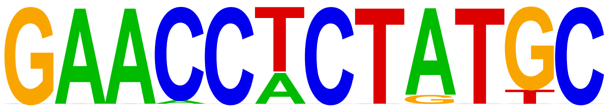	1e-38	-8.883e+01	1.57%	0.01%	60.2bp (32.6bp)	RUNX3/MA0684.2/Jaspar(0.605) More Information | Similar Motifs Found	motif file (matrix)
13	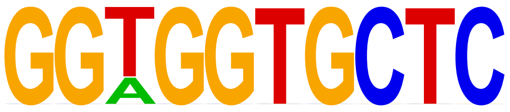	1e-37	-8.749e+01	2.91%	0.13%	44.5bp (52.9bp)	PB0196.1_Zbtb7b_2/Jaspar(0.654) More Information | Similar Motifs Found	motif file (matrix)
14	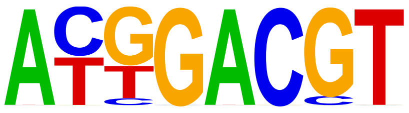	1e-27	-6.220e+01	5.90%	1.30%	43.9bp (75.4bp)	MF0002.1_bZIP_CREB/G-box-like_subclass/Jaspar(0.794) More Information | Similar Motifs Found	motif file (matrix)
15	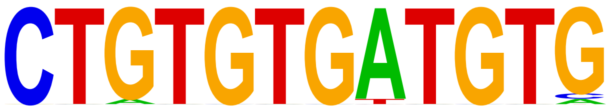	1e-25	-5.893e+01	1.12%	0.01%	76.3bp (37.3bp)	POL009.1_DCE_S_II/Jaspar(0.586) More Information | Similar Motifs Found	motif file (matrix)
16	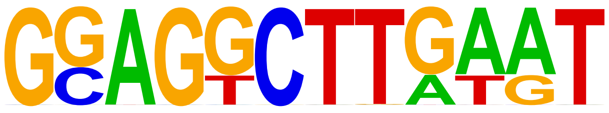	1e-24	-5.543e+01	0.82%	0.00%	49.4bp (3.5bp)	Nr2e3/MA0164.1/Jaspar(0.599) More Information | Similar Motifs Found	motif file (matrix)
17		1e-21	-4.959e+01	0.75%	0.00%	68.5bp (24.6bp)	NFATC2/MA0152.1/Jaspar(0.749) More Information | Similar Motifs	motif file

	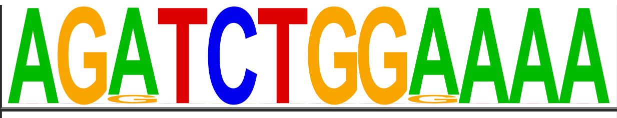						Found	(matrix)
18	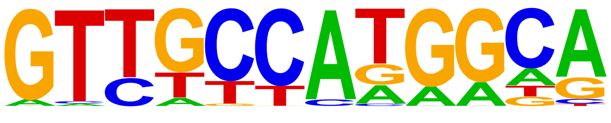	1e-20	-4.606e+01	4.63%	1.09%	50.4bp (75.1bp)	RFX3/MA0798.2/Jaspar(0.931) More Information | Similar Motifs Found	motif file (matrix)
19	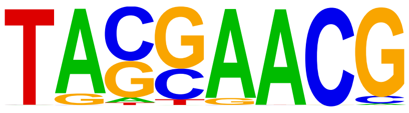	1e-18	-4.277e+01	8.89%	3.54%	54.4bp (72.4bp)	RFX7/MA1554.1/Jaspar(0.801) More Information | Similar Motifs Found	motif file (matrix)
20	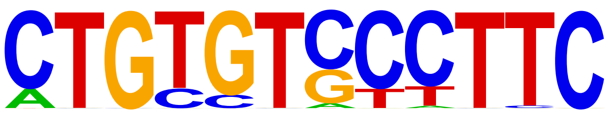	1e-16	-3.872e+01	1.35%	0.07%	55.5bp (57.5bp)	PU.1-IRF(ETS:IRF)/Bcell-PU.1-ChIP-Seq(GSE21512)/Homer(0.637) More Information | Similar Motifs Found	motif file (matrix)
21	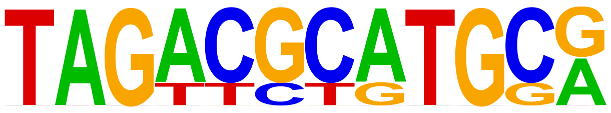	1e-16	-3.866e+01	0.75%	0.01%	56.8bp (44.0bp)	PB0147.1_Max_2/Jaspar(0.598) More Information | Similar Motifs Found	motif file (matrix)
22	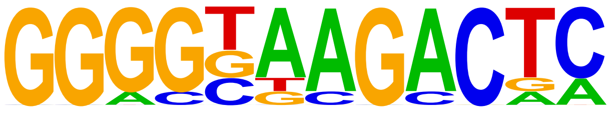	1e-16	-3.819e+01	0.60%	0.00%	36.5bp (43.9bp)	Tbox:Smad(T-box,MAD)/ESCd5-Smad2_3-ChIP-Seq(GSE29422)/Homer(0.655) More Information | Similar Motifs Found	motif file (matrix)
23	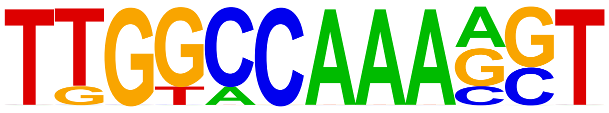	1e-14	-3.361e+01	0.75%	0.01%	84.9bp (38.7bp)	PPARE(NR),DR1/3T3L1-Pparg-ChIP-Seq(GSE13511)/Homer(0.693) More Information | Similar Motifs Found	motif file (matrix)
24	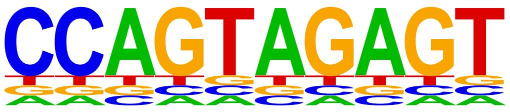	1e-14	-3.267e+01	0.60%	0.01%	83.5bp (37.8bp)	ZBTB32/MA1580.1/Jaspar(0.638) More Information | Similar Motifs Found	motif file (matrix)
25	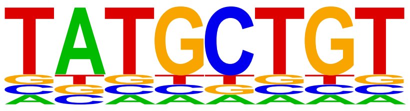	1e-12	-2.887e+01	1.79%	0.25%	70.9bp (63.1bp)	ZNF317/MA1593.1/Jaspar(0.802) More Information | Similar Motifs Found	motif file (matrix)
26	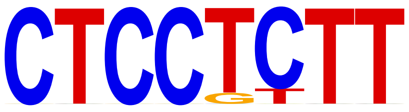	1e-12	-2.795e+01	7.40%	3.36%	51.9bp (70.6bp)	IKZF1/MA1508.1/Jaspar(0.676) More Information | Similar Motifs Found	motif file (matrix)
27	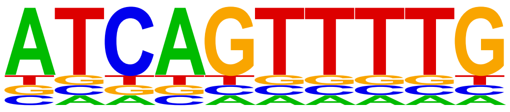	1e-12	-2.784e+01	0.52%	0.01%	43.9bp (35.4bp)	SIX2/MA1119.1/Jaspar(0.651) More Information | Similar Motifs Found	motif file (matrix)
28 *	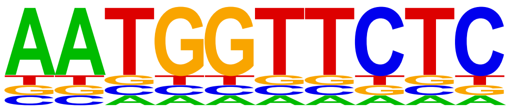	1e-11	-2.718e+01	0.60%	0.01%	51.0bp (26.9bp)	PB0062.1_Sox12_1/Jaspar(0.673) More Information | Similar Motifs Found	motif file (matrix)
29 *	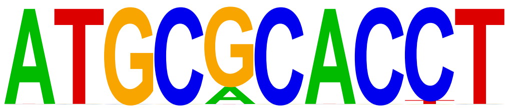	1e-8	-1.887e+01	0.75%	0.06%	56.9bp (63.0bp)	PB0089.1_Tcfe2a_1/Jaspar(0.702) More Information | Similar Motifs Found	motif file (matrix)
30 *	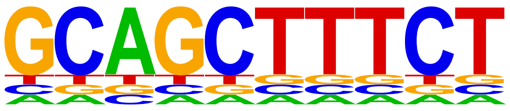	1e-7	-1.809e+01	0.75%	0.06%	53.8bp (55.9bp)	PB0137.1_Irf3_2/Jaspar(0.705) More Information | Similar Motifs Found	motif file (matrix)
