# Supplementary material for: Transcription factor PBX4 regulates limb development and haematopoiesis in mice
Source: Cell Prolif. 2024 Jan 17;57(5):e13580. doi: 10.1111/cpr.13580 (PMC11056705; doi:10.1111/cpr.13580)
Supplement: Supplementary file 8 — Table S4_2. Known motifs enriched in PBX4 ChIP‐seq peaks. [file CPR-57-e13580-s008.pdf]

Homer Known Motif Enrichment Results (homer/Flag.vs.input)

Homer *de novo* Motif Results
Gene Ontology Enrichment Results
Known Motif Enrichment Results (txt file)
Total Target Sequences = 1338, Total Background Sequences = 41910

Rank	Motif	Name	P-value	log P-pvalue	q-value (Benjamini)	# Target Sequences with Motif	% of Targets Sequences with Motif	# Background Sequences with Motif	% of Background Sequences with Motif	Motif File	SVG
1	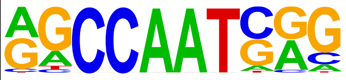	NFY(CCAAT)/Promoter/Homer	1e-617	-1.421e+03	0.0000	886.0	66.22%	3361.2	8.02%	motif file (matrix)	svg
2	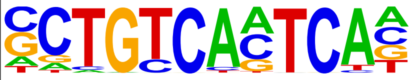	Pbx3(Homeobox)/GM12878-PBX3-ChIP-Seq(GSE32465)/Homer	1e-601	-1.385e+03	0.0000	608.0	45.44%	976.8	2.33%	motif file (matrix)	svg
3	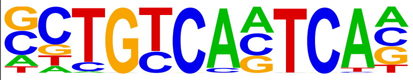	Pknox1(Homeobox)/ES-Prep1-ChIP-Seq(GSE63282)/Homer	1e-596	-1.374e+03	0.0000	607.0	45.37%	988.1	2.36%	motif file (matrix)	svg
4	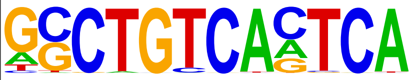	PBX1(Homeobox)/MCF7-PBX1-ChIP-Seq(GSE28007)/Homer	1e-354	-8.164e+02	0.0000	321.0	23.99%	344.8	0.82%	motif file (matrix)	svg
5	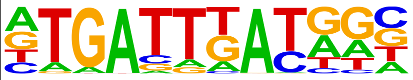	PBX2(Homeobox)/K562-PBX2-ChIP-Seq(Encode)/Homer	1e-251	-5.799e+02	0.0000	422.0	31.54%	1614.9	3.85%	motif file (matrix)	svg
6	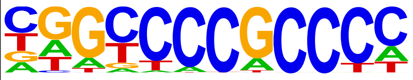	Sp2(Zf)/HEK293-Sp2.eGFP-ChIP-Seq(Encode)/Homer	1e-174	-4.019e+02	0.0000	842.0	62.93%	10946.9	26.12%	motif file (matrix)	svg
7	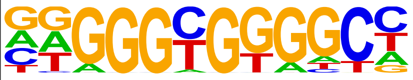	KLF1(Zf)/HUDEP2-KLF1-CutnRun(GSE136251)/Homer	1e-170	-3.924e+02	0.0000	662.0	49.48%	6895.6	16.45%	motif file (matrix)	svg
8	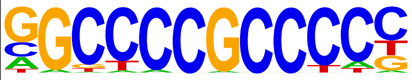	Sp1(Zf)/Promoter/Homer	1e-160	-3.702e+02	0.0000	461.0	34.45%	3434.1	8.19%	motif file (matrix)	svg
9	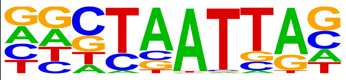	En1(Homeobox)/SUM149-EN1-ChIP-Seq(GSE120957)/Homer	1e-153	-3.541e+02	0.0000	493.0	36.85%	4120.0	9.83%	motif file (matrix)	svg
10	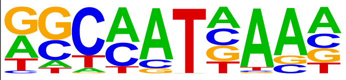	Hoxa9(Homeobox)/ChickenMSG-Hoxa9.Flag-ChIP-Seq(GSE86088)/Homer	1e-151	-3.483e+02	0.0000	603.0	45.07%	6257.2	14.93%	motif file (matrix)	svg
11	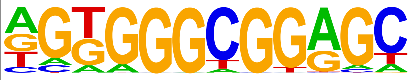	Sp5(Zf)/mES-Sp5.Flag-ChIP-Seq(GSE72989)/Homer	1e-142	-3.270e+02	0.0000	680.0	50.82%	8230.6	19.64%	motif file (matrix)	svg
12	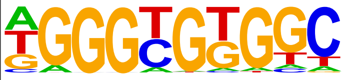	KLF5(Zf)/LoVo-KLF5-ChIP-Seq(GSE49402)/Homer	1e-110	-2.534e+02	0.0000	661.0	49.40%	9061.8	21.62%	motif file (matrix)	svg
13	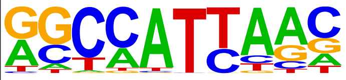	Nanog(Homeobox)/mES-Nanog-ChIP-Seq(GSE11724)/Homer	1e-105	-2.428e+02	0.0000	797.0	59.57%	12807.3	30.56%	motif file (matrix)	svg
14	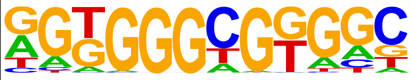	KLF14(Zf)/HEK293-KLF14.GFP-ChIP-Seq(GSE58341)/Homer	1e-101	-2.338e+02	0.0000	774.0	57.85%	12381.2	29.54%	motif file (matrix)	svg
15	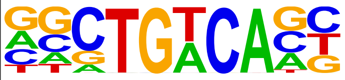	Meis1(Homeobox)/MastCells-Meis1-ChIP-Seq(GSE48085)/Homer	1e-97	-2.240e+02	0.0000	527.0	39.39%	6521.5	15.56%	motif file (matrix)	svg
16	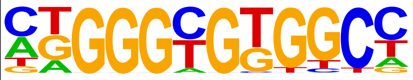	KLF6(Zf)/PDAC-KLF6-ChIP-Seq(GSE64557)/Homer	1e-93	-2.150e+02	0.0000	563.0	42.08%	7489.6	17.87%	motif file (matrix)	svg
17	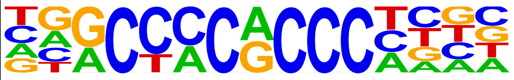	KLF3(Zf)/MEF-Klf3-ChIP-Seq(GSE44748)/Homer	1e-81	-1.882e+02	0.0000	370.0	27.65%	3891.2	9.29%	motif file (matrix)	svg
18	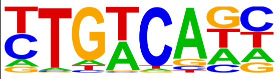	Tgif1(Homeobox)/mES-Tgif1-ChIP-Seq(GSE55404)/Homer	1e-79	-1.830e+02	0.0000	634.0	47.38%	9884.5	23.59%	motif file (matrix)	svg
19	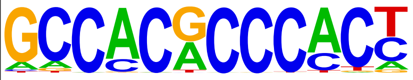	Klf9(Zf)/GBM-Klf9-ChIP-Seq(GSE62211)/Homer	1e-76	-1.764e+02	0.0000	316.0	23.62%	3061.4	7.31%	motif file (matrix)	svg
20	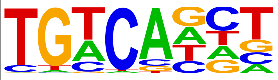	Tgif2(Homeobox)/mES-Tgif2-ChIP-Seq(GSE55404)/Homer	1e-58	-1.336e+02	0.0000	623.0	46.56%	10895.3	26.00%	motif file (matrix)	svg
21	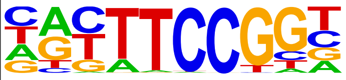	Elk4(ETS)/Hela-Elk4-ChIP-Seq(GSE31477)/Homer	1e-56	-1.309e+02	0.0000	317.0	23.69%	3761.7	8.98%	motif file (matrix)	svg
22	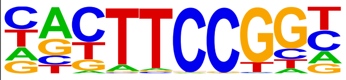	Elk1(ETS)/Hela-Elk1-ChIP-Seq(GSE31477)/Homer	1e-51	-1.188e+02	0.0000	304.0	22.72%	3716.6	8.87%	motif file (matrix)	svg
23	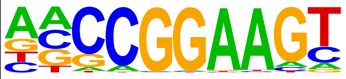	ELF1(ETS)/Jurkat-ELF1-ChIP-Seq(SRA014231)/Homer	1e-49	-1.144e+02	0.0000	281.0	21.00%	3334.7	7.96%	motif file (matrix)	svg
24	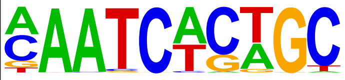	Gfi1b(Zf)/HPC7-Gfi1b-ChIP-Seq(GSE22178)/Homer	1e-47	-1.098e+02	0.0000	181.0	13.53%	1590.2	3.79%	motif file (matrix)	svg
25	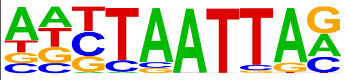	Lhx3(Homeobox)/Neuron-Lhx3-ChIP-Seq(GSE31456)/Homer	1e-46	-1.077e+02	0.0000	265.0	19.81%	3137.2	7.49%	motif file (matrix)	svg
26	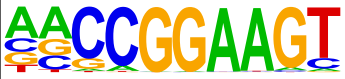	ETS(ETS)/Promoter/Homer	1e-44	-1.021e+02	0.0000	198.0	14.80%	1976.8	4.72%	motif file (matrix)	svg
27	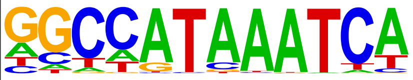	Hoxc9(Homeobox)/Ainv15-Hoxc9-ChIP-Seq(GSE21812)/Homer	1e-43	-1.005e+02	0.0000	124.0	9.27%	831.1	1.98%	motif file (matrix)	svg
28	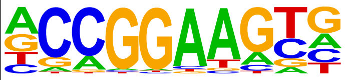	ETV4(ETS)/HepG2-ETV4-ChIP-Seq(ENCODE)/Homer	1e-37	-8.631e+01	0.0000	372.0	27.80%	5952.2	14.20%	motif file (matrix)	svg
29	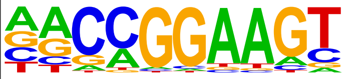	GABPA(ETS)/Jurkat-GABPa-ChIP-Seq(GSE17954)/Homer	1e-34	-7.875e+01	0.0000	289.0	21.60%	4244.2	10.13%	motif file (matrix)	svg
30	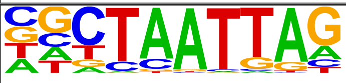	LHX9(Homeobox)/Hct116-LHX9.V5-ChIP-Seq(GSE116822)/Homer	1e-33	-7.602e+01	0.0000	222.0	16.59%	2874.2	6.86%	motif file	svg

										(matrix)	
31	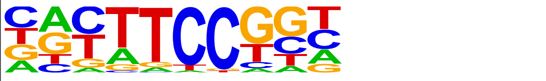	Fli1(ETS)/CD8-FLI-ChIP-Seq(GSE20898)/Homer	1e-31	-7.273e+01	0.0000	341.0	25.49%	5607.4	13.38%	motif file (matrix)	svg
32	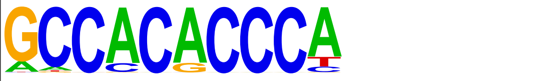	Klf4(Zf)/mES-Klf4-ChIP-Seq(GSE11431)/Homer	1e-29	-6.728e+01	0.0000	190.0	14.20%	2403.2	5.73%	motif file (matrix)	svg
33	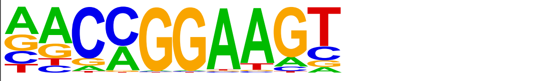	ETV1(ETS)/GIST48-ETV1-ChIP-Seq(GSE22441)/Homer	1e-29	-6.721e+01	0.0000	350.0	26.16%	5995.2	14.31%	motif file (matrix)	svg
34	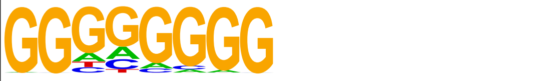	Maz(Zf)/HepG2-Maz-ChIP-Seq(GSE31477)/Homer	1e-24	-5.746e+01	0.0000	495.0	37.00%	10132.3	24.18%	motif file (matrix)	svg
35	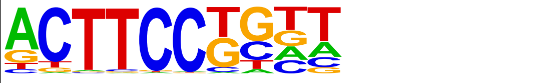	Elf4(ETS)/BMDM-Elf4-ChIP-Seq(GSE88699)/Homer	1e-24	-5.609e+01	0.0000	261.0	19.51%	4222.8	10.08%	motif file (matrix)	svg
36	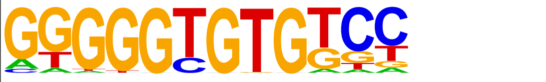	KLF10(Zf)/HEK293-KLF10.GFP-ChIP-Seq(GSE58341)/Homer	1e-21	-4.883e+01	0.0000	197.0	14.72%	2977.4	7.10%	motif file (matrix)	svg
37	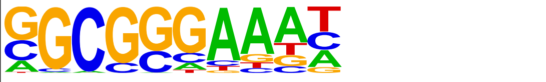	E2F4(E2F)/K562-E2F4-ChIP-Seq(GSE31477)/Homer	1e-19	-4.484e+01	0.0000	243.0	18.16%	4155.8	9.92%	motif file (matrix)	svg
38	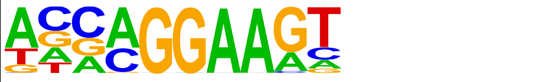	EHF(ETS)/LoVo-EHF-ChIP-Seq(GSE49402)/Homer	1e-19	-4.467e+01	0.0000	252.0	18.83%	4376.9	10.44%	motif file (matrix)	svg
39	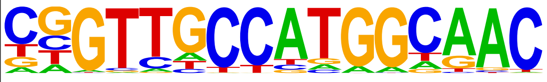	RFX(HTH)/K562-RFX3-ChIP-Seq(SRA012198)/Homer	1e-18	-4.310e+01	0.0000	60.0	4.48%	455.4	1.09%	motif file (matrix)	svg
40	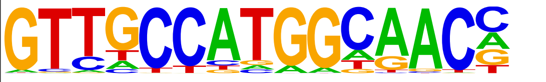	Rfx2(HTH)/LoVo-RFX2-ChIP-Seq(GSE49402)/Homer	1e-17	-4.027e+01	0.0000	60.0	4.48%	484.8	1.16%	motif file (matrix)	svg
41	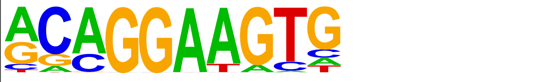	ETS1(ETS)/Jurkat-ETS1-ChIP-Seq(GSE17954)/Homer	1e-16	-3.784e+01	0.0000	242.0	18.09%	4381.2	10.45%	motif file (matrix)	svg
42	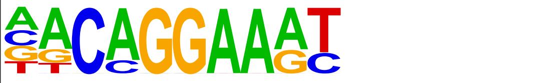	EWS:FLI1-fusion(ETS)/SK_N_MC-EWS:FLI1-ChIP-Seq(SRA014231)/Homer	1e-16	-3.765e+01	0.0000	151.0	11.29%	2268.7	5.41%	motif file (matrix)	svg
43	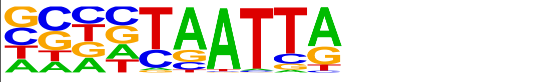	DLX1(Homeobox)/BasalGanglia-Dlx1-ChIP-seq(GSE124936)/Homer	1e-16	-3.765e+01	0.0000	159.0	11.88%	2444.2	5.83%	motif file (matrix)	svg
44	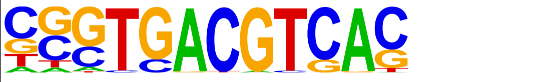	CRE(bZIP)/Promoter/Homer	1e-14	-3.356e+01	0.0000	102.0	7.62%	1339.0	3.20%	motif file (matrix)	svg
45	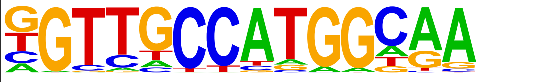	Rfx1(HTH)/NPC-H3K4me1-ChIP-Seq(GSE16256)/Homer	1e-13	-3.213e+01	0.0000	75.0	5.61%	846.4	2.02%	motif file (matrix)	svg
46	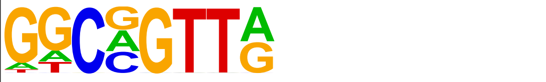	MYB(HTH)/ERMYB-Myb-ChIPSeq(GSE22095)/Homer	1e-13	-3.096e+01	0.0000	304.0	22.72%	6263.5	14.95%	motif file (matrix)	svg
47	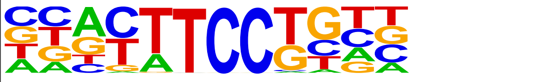	Etv2(ETS)/ES-ER71-ChIP-Seq(GSE59402)/Homer	1e-12	-2.927e+01	0.0000	190.0	14.20%	3443.4	8.22%	motif file (matrix)	svg
48	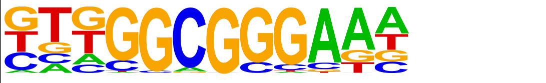	E2F3(E2F)/MEF-E2F3-ChIP-Seq(GSE71376)/Homer	1e-12	-2.897e+01	0.0000	254.0	18.98%	5058.2	12.07%	motif file (matrix)	svg
49	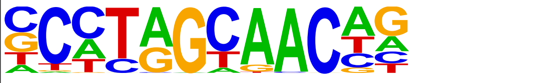	Rfx5(HTH)/GM12878-Rfx5-ChIP-Seq(GSE31477)/Homer	1e-12	-2.804e+01	0.0000	83.0	6.20%	1077.1	2.57%	motif file (matrix)	svg
50	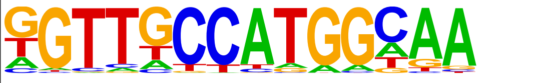	X-box(HTH)/NPC-H3K4me1-ChIP-Seq(GSE16256)/Homer	1e-12	-2.767e+01	0.0000	45.0	3.36%	396.1	0.95%	motif file (matrix)	svg
51	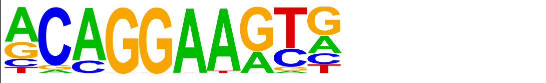	ERG(ETS)/VCaP-ERG-ChIP-Seq(GSE14097)/Homer	1e-11	-2.680e+01	0.0000	284.0	21.23%	5951.8	14.20%	motif file (matrix)	svg
52	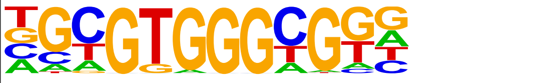	Egr2(Zf)/Thymocytes-Egr2-ChIP-Seq(GSE34254)/Homer	1e-10	-2.424e+01	0.0000	87.0	6.50%	1247.6	2.98%	motif file (matrix)	svg
53	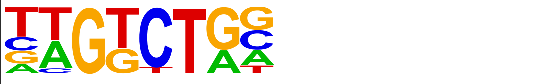	Smad3(MAD)/NPC-Smad3-ChIP-Seq(GSE36673)/Homer	1e-10	-2.338e+01	0.0000	453.0	33.86%	10860.6	25.92%	motif file (matrix)	svg
54	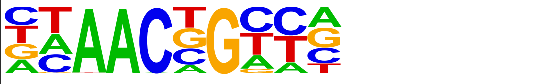	BMYB(HTH)/Hela-BMYB-ChIP-Seq(GSE27030)/Homer	1e-9	-2.132e+01	0.0000	225.0	16.82%	4693.5	11.20%	motif file (matrix)	svg
55	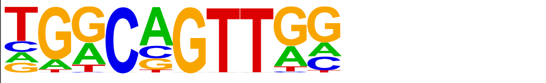	AMYB(HTH)/Testes-AMYB-ChIP-Seq(GSE44588)/Homer	1e-8	-2.023e+01	0.0000	238.0	17.79%	5097.5	12.16%	motif file (matrix)	svg
56	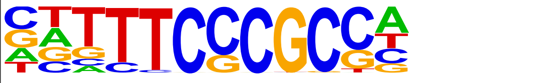	E2F7(E2F)/Hela-E2F7-ChIP-Seq(GSE32673)/Homer	1e-8	-2.009e+01	0.0000	72.0	5.38%	1037.8	2.48%	motif file (matrix)	svg
57	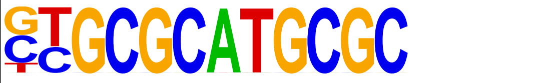	NRF(NRF)/Promoter/Homer	1e-8	-1.905e+01	0.0000	100.0	7.47%	1687.9	4.03%	motif file (matrix)	svg
58	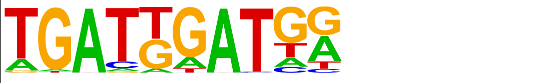	HOXA1(Homeobox)/mES-Hoxa1-ChIP-Seq(SRP084292)/Homer	1e-7	-1.819e+01	0.0000	49.0	3.66%	614.2	1.47%	motif file (matrix)	svg
59	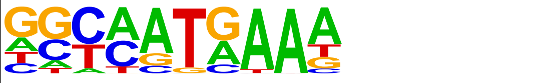	Hoxd10(Homeobox)/ChickenMSG-Hoxd10.Flag-ChIP-Seq(GSE86088)/Homer	1e-7	-1.818e+01	0.0000	121.0	9.04%	2216.0	5.29%	motif file (matrix)	svg
60	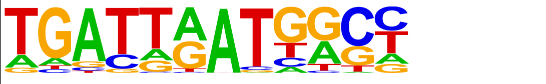	Hoxb4(Homeobox)/ES-Hoxb4-ChIP-Seq(GSE34014)/Homer	1e-7	-1.772e+01	0.0000	39.0	2.91%	435.6	1.04%	motif file (matrix)	svg
61	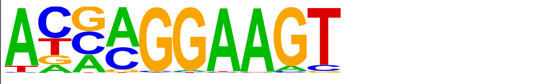	ELF5(ETS)/T47D-ELF5-ChIP-Seq(GSE30407)/Homer	1e-7	-1.771e+01	0.0000	135.0	10.09%	2577.9	6.15%	motif file (matrix)	svg
62	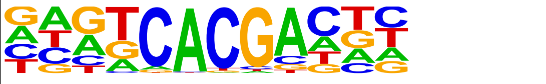	Npas4(bHLH)/Neuron-Npas4-ChIP-Seq(GSE127793)/Homer	1e-7	-1.769e+01	0.0000	191.0	14.28%	4004.1	9.55%	motif file (matrix)	svg
63	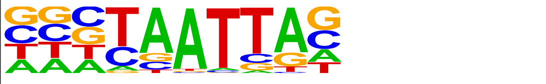	DLX2(Homeobox)/BasalGanglia-Dlx2-ChIP-seq(GSE124936)/Homer	1e-7	-1.719e+01	0.0000	142.0	10.61%	2775.9	6.62%	motif file (matrix)	svg

64	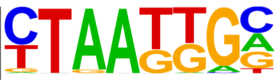	Isl1(Homeobox)/Neuron-Isl1-ChIP-Seq(GSE31456)/Homer	1e-6	-1.518e+01	0.0000	202.0	15.10%	4445.7	10.61%	motif file (matrix)	svg
65	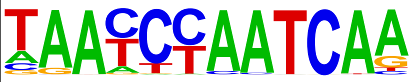	Duxbl(Homeobox)/NIH3T3-Duxbl.HA-ChIP-Seq(GSE119782)/Homer	1e-6	-1.452e+01	0.0000	15.0	1.12%	92.3	0.22%	motif file (matrix)	svg
66	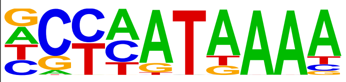	Hoxd13(Homeobox)/ChickenMSG-Hoxd13.Flag-ChIP-Seq(GSE86088)/Homer	1e-6	-1.447e+01	0.0000	124.0	9.27%	2455.7	5.86%	motif file (matrix)	svg
67	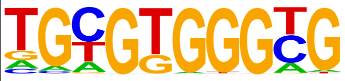	Egr1(Zf)/K562-Egr1-ChIP-Seq(GSE32465)/Homer	1e-6	-1.413e+01	0.0000	202.0	15.10%	4514.1	10.77%	motif file (matrix)	svg
68	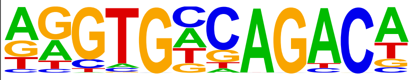	Tbox:Smad(T-box,MAD)/ESCd5-Smad2_3-ChIP-Seq(GSE29422)/Homer	1e-6	-1.407e+01	0.0000	44.0	3.29%	605.0	1.44%	motif file (matrix)	svg
69	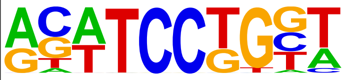	SPDEF(ETS)/VCaP-SPDEF-ChIP-Seq(SRA014231)/Homer	1e-5	-1.374e+01	0.0000	174.0	13.00%	3790.5	9.05%	motif file (matrix)	svg
70	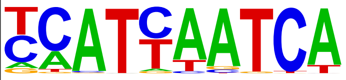	Pdx1(Homeobox)/Islet-Pdx1-ChIP-Seq(SRA008281)/Homer	1e-5	-1.360e+01	0.0000	99.0	7.40%	1875.0	4.47%	motif file (matrix)	svg
71	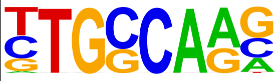	NF1-halfsite(CTF)/LNCaP-NF1-ChIP-Seq(Unpublished)/Homer	1e-5	-1.334e+01	0.0000	284.0	21.23%	6839.8	16.32%	motif file (matrix)	svg
72	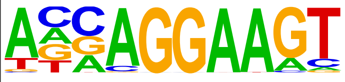	ELF3(ETS)/PDAC-ELF3-ChIP-Seq(GSE64557)/Homer	1e-5	-1.320e+01	0.0000	110.0	8.22%	2166.6	5.17%	motif file (matrix)	svg
73	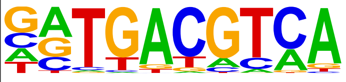	Atf1(bZIP)/K562-ATF1-ChIP-Seq(GSE31477)/Homer	1e-5	-1.318e+01	0.0000	113.0	8.45%	2242.6	5.35%	motif file (matrix)	svg
74	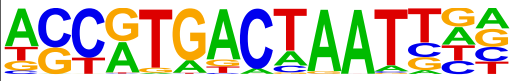	PAX3:FKHR-fusion(Paired,Homeobox)/Rh4-PAX3:FKHR-ChIP-Seq(GSE19063)/Homer	1e-5	-1.296e+01	0.0000	28.0	2.09%	316.1	0.75%	motif file (matrix)	svg
75	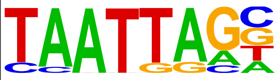	Lhx2(Homeobox)/HFSC-Lhx2-ChIP-Seq(GSE48068)/Homer	1e-5	-1.285e+01	0.0000	94.0	7.03%	1786.3	4.26%	motif file (matrix)	svg
76	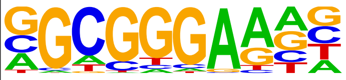	E2F6(E2F)/Hela-E2F6-ChIP-Seq(GSE31477)/Homer	1e-5	-1.204e+01	0.0000	191.0	14.28%	4362.3	10.41%	motif file (matrix)	svg
77	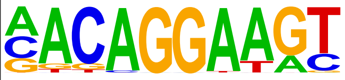	Ets1-distal(ETS)/CD4+-PolII-ChIP-Seq(Barski_et_al.)/Homer	1e-5	-1.202e+01	0.0000	52.0	3.89%	830.9	1.98%	motif file (matrix)	svg
78	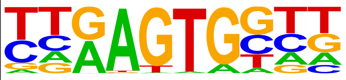	Bapx1(Homeobox)/VertebralCol-Bapx1-ChIP-Seq(GSE36672)/Homer	1e-5	-1.193e+01	0.0000	268.0	20.03%	6509.7	15.53%	motif file (matrix)	svg
79	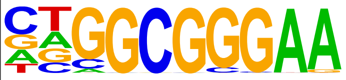	E2F1(E2F)/Hela-E2F1-ChIP-Seq(GSE22478)/Homer	1e-5	-1.187e+01	0.0000	110.0	8.22%	2233.5	5.33%	motif file (matrix)	svg
80	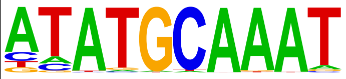	Oct2(POU,Homeobox)/Bcell-Oct2-ChIP-Seq(GSE21512)/Homer	1e-5	-1.179e+01	0.0000	27.0	2.02%	318.2	0.76%	motif file (matrix)	svg
81	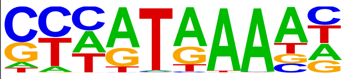	Hoxa13(Homeobox)/ChickenMSG-Hoxa13.Flag-ChIP-Seq(GSE86088)/Homer	1e-5	-1.175e+01	0.0000	188.0	14.05%	4302.9	10.27%	motif file (matrix)	svg
82	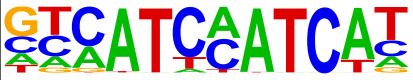	HOXA2(Homeobox)/mES-Hoxa2-ChIP-Seq(Donaldson_et_al.)/Homer	1e-4	-1.131e+01	0.0001	23.0	1.72%	253.2	0.60%	motif file (matrix)	svg
83	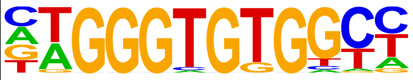	EKLF(Zf)/Erythrocyte-Klf1-ChIP-Seq(GSE20478)/Homer	1e-4	-1.006e+01	0.0002	51.0	3.81%	872.1	2.08%	motif file (matrix)	svg
84	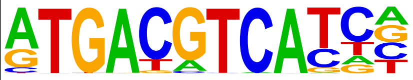	JunD(bZIP)/K562-JunD-ChIP-Seq/Homer	1e-4	-9.907e+00	0.0003	30.0	2.24%	416.4	0.99%	motif file (matrix)	svg
85	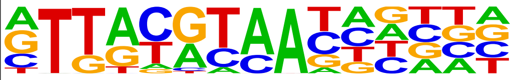	NFIL3(bZIP)/HepG2-NFIL3-ChIP-Seq(Encode)/Homer	1e-4	-9.849e+00	0.0003	64.0	4.78%	1187.3	2.83%	motif file (matrix)	svg
86	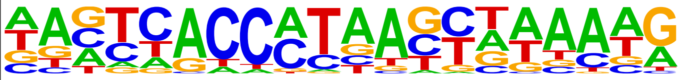	PSE(SNAPc)/K562-mStart-Seq/Homer	1e-4	-9.692e+00	0.0003	50.0	3.74%	862.8	2.06%	motif file (matrix)	svg
87	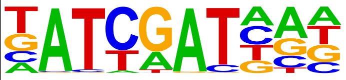	CUX1(Homeobox)/K562-CUX1-ChIP-Seq(GSE92882)/Homer	1e-4	-9.689e+00	0.0003	65.0	4.86%	1218.4	2.91%	motif file (matrix)	svg
88	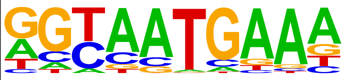	Hoxa10(Homeobox)/ChickenMSG-Hoxa10.Flag-ChIP-Seq(GSE86088)/Homer	1e-4	-9.531e+00	0.0004	55.0	4.11%	985.4	2.35%	motif file (matrix)	svg
89	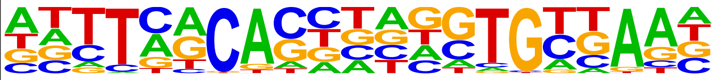	Brachyury(T-box)/Mesoendoderm-Brachyury-ChIP-exo(GSE54963)/Homer	1e-4	-9.403e+00	0.0004	37.0	2.77%	579.0	1.38%	motif file (matrix)	svg
90	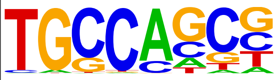	HIC1(Zf)/Treg-ZBTB29-ChIP-Seq(GSE99889)/Homer	1e-3	-9.158e+00	0.0005	385.0	28.77%	10189.8	24.32%	motif file (matrix)	svg
91	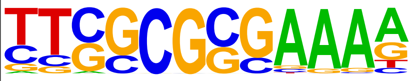	E2F(E2F)/Hela-CellCycle-Expression/Homer	1e-3	-8.583e+00	0.0009	31.0	2.32%	471.8	1.13%	motif file (matrix)	svg
92	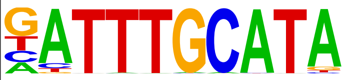	Oct11(POU,Homeobox)/NCIH1048-POU2F3-ChIP-seq(GSE115123)/Homer	1e-3	-8.553e+00	0.0009	29.0	2.17%	429.7	1.03%	motif file (matrix)	svg
93	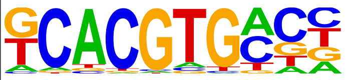	bHLHE41(bHLH)/proB-Bhlhe41-ChIP-Seq(GSE93764)/Homer	1e-3	-8.440e+00	0.0010	252.0	18.83%	6388.2	15.24%	motif file (matrix)	svg
94	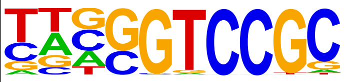	HINFP(Zf)/K562-HINFP.eGFP-ChIP-Seq(Encode)/Homer	1e-3	-8.287e+00	0.0012	147.0	10.99%	3447.2	8.23%	motif file (matrix)	svg
95	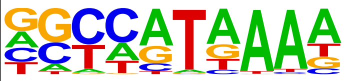	Hoxd11(Homeobox)/ChickenMSG-Hoxd11.Flag-ChIP-Seq(GSE86088)/Homer	1e-3	-7.616e+00	0.0023	193.0	14.42%	4790.9	11.43%	motif file (matrix)	svg
96	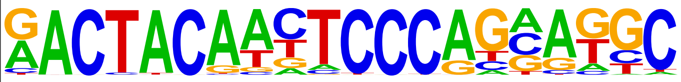	Ronin(THAP)/ES-Thap11-ChIP-Seq(GSE51522)/Homer	1e-3	-7.586e+00	0.0023	15.0	1.12%	170.0	0.41%	motif file (matrix)	svg

97	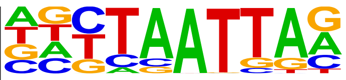	Lhx1(Homeobox)/EmbryoCarcinoma-Lhx1-ChIP-Seq(GSE70957)/Homer	1e-3	-7.571e+00	0.0023	83.0	6.20%	1781.7	4.25%	motif file (matrix)	svg
98	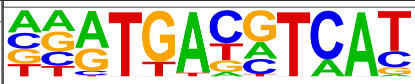	CREB5(bZIP)/LNCaP-CREB5.V5-ChIP-Seq(GSE137775)/Homer	1e-3	-7.513e+00	0.0025	51.0	3.81%	973.2	2.32%	motif file (matrix)	svg
99	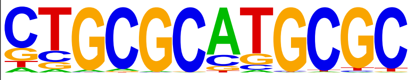	NRF1(NRF)/MCF7-NRF1-ChIP-Seq(Unpublished)/Homer	1e-3	-7.487e+00	0.0025	80.0	5.98%	1708.9	4.08%	motif file (matrix)	svg
100	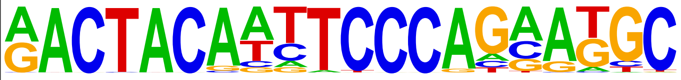	GFY-Staf(?,Zf)/Promoter/Homer	1e-3	-7.347e+00	0.0028	17.0	1.27%	212.8	0.51%	motif file (matrix)	svg
101	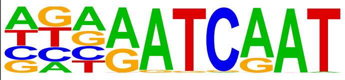	Cux2(Homeobox)/Liver-Cux2-ChIP-Seq(GSE35985)/Homer	1e-3	-7.267e+00	0.0030	45.0	3.36%	838.6	2.00%	motif file (matrix)	svg
102	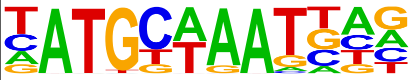	Brn1(POU,Homeobox)/NPC-Brn1-ChIP-Seq(GSE35496)/Homer	1e-3	-7.248e+00	0.0031	27.0	2.02%	422.4	1.01%	motif file (matrix)	svg
103	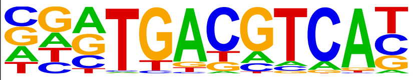	Atf7(bZIP)/3T3L1-Atf7-ChIP-Seq(GSE56872)/Homer	1e-3	-7.223e+00	0.0031	76.0	5.68%	1621.0	3.87%	motif file (matrix)	svg
104	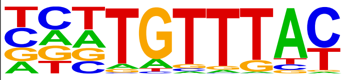	FOXK1(Forkhead)/HEK293-FOXK1-ChIP-Seq(GSE51673)/Homer	1e-3	-7.090e+00	0.0035	76.0	5.68%	1628.7	3.89%	motif file (matrix)	svg
105	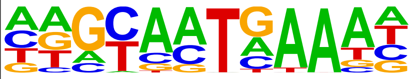	Hoxd12(Homeobox)/ChickenMSG-Hoxd12.Flag-ChIP-Seq(GSE86088)/Homer	1e-3	-6.909e+00	0.0042	127.0	9.49%	3011.7	7.19%	motif file (matrix)	svg
106	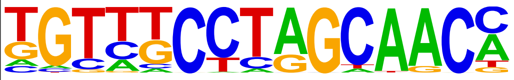	Rfx6(HTH)/Min6b1-Rfx6.HA-ChIP-Seq(GSE62844)/Homer	1e-2	-6.557e+00	0.0059	172.0	12.86%	4302.0	10.27%	motif file (matrix)	svg
107	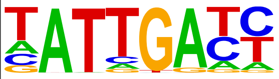	Hnf6b(Homeobox)/LNCaP-Hnf6b-ChIP-Seq(GSE106305)/Homer	1e-2	-6.505e+00	0.0061	77.0	5.75%	1691.2	4.04%	motif file (matrix)	svg
108	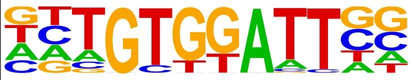	Foxh1(Forkhead)/hESC-FOXH1-ChIP-Seq(GSE29422)/Homer	1e-2	-6.391e+00	0.0068	61.0	4.56%	1281.2	3.06%	motif file (matrix)	svg
109	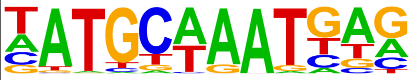	Oct6(POU,Homeobox)/NPC-Pou3f1-ChIP-Seq(GSE35496)/Homer	1e-2	-6.082e+00	0.0092	33.0	2.47%	599.7	1.43%	motif file (matrix)	svg
110	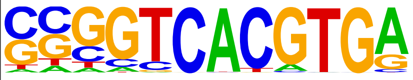	E-box(bHLH)/Promoter/Homer	1e-2	-6.061e+00	0.0093	30.0	2.24%	529.8	1.26%	motif file (matrix)	svg
111	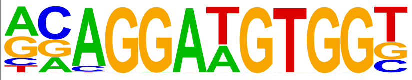	ETS:RUNX(ETS,Runt)/Jurkat-RUNX1-ChIP-Seq(GSE17954)/Homer	1e-2	-6.055e+00	0.0093	22.0	1.64%	347.4	0.83%	motif file (matrix)	svg
112	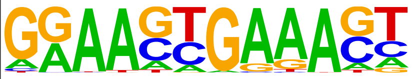	IRF8(IRF)/BMDM-IRF8-ChIP-Seq(GSE77884)/Homer	1e-2	-5.706e+00	0.0131	36.0	2.69%	687.4	1.64%	motif file (matrix)	svg
113	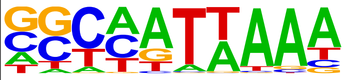	Unknown(Homeobox)/Limb-p300-ChIP-Seq/Homer	1e-2	-5.572e+00	0.0148	43.0	3.21%	867.4	2.07%	motif file (matrix)	svg
114	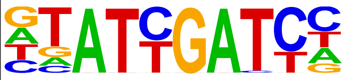	HNF6(Homeobox)/Liver-Hnf6-ChIP-Seq(ERP000394)/Homer	1e-2	-5.570e+00	0.0148	54.0	4.04%	1148.9	2.74%	motif file (matrix)	svg
115	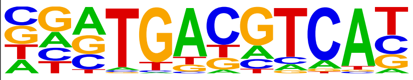	Atf2(bZIP)/3T3L1-Atf2-ChIP-Seq(GSE56872)/Homer	1e-2	-5.535e+00	0.0151	55.0	4.11%	1176.4	2.81%	motif file (matrix)	svg
116	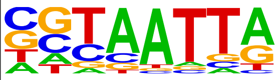	DLX5(Homeobox)/BasalGanglia-Dlx5-ChIP-seq(GSE124936)/Homer	1e-2	-5.523e+00	0.0151	59.0	4.41%	1281.0	3.06%	motif file (matrix)	svg
117	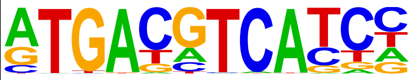	c-Jun-CRE(bZIP)/K562-cJun-ChIP-Seq(GSE31477)/Homer	1e-2	-5.429e+00	0.0165	47.0	3.51%	976.7	2.33%	motif file (matrix)	svg
118	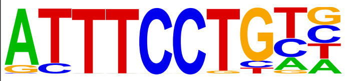	EWS:ERG-fusion(ETS)/CADO_ES1-EWS:ERG-ChIP-Seq(SRA014231)/Homer	1e-2	-5.167e+00	0.0213	78.0	5.83%	1813.8	4.33%	motif file (matrix)	svg
119	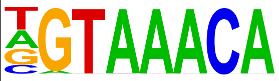	Foxo3(Forkhead)/U2OS-Foxo3-ChIP-Seq(E-MTAB-2701)/Homer	1e-2	-5.040e+00	0.0239	52.0	3.89%	1128.9	2.69%	motif file (matrix)	svg
120	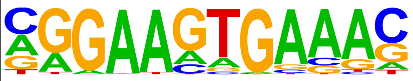	PU.1-IRF(ETS:IRF)/Bcell-PU.1-ChIP-Seq(GSE21512)/Homer	1e-2	-5.011e+00	0.0244	141.0	10.54%	3586.2	8.56%	motif file (matrix)	svg
121	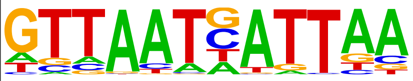	HNF1b(Homeobox)/PDAC-HNF1B-ChIP-Seq(GSE64557)/Homer	1e-2	-4.787e+00	0.0303	10.0	0.75%	126.9	0.30%	motif file (matrix)	svg
122	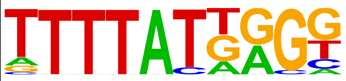	HOXB13(Homeobox)/ProstateTumor-HOXB13-ChIP-Seq(GSE56288)/Homer	1e-2	-4.779e+00	0.0303	69.0	5.16%	1599.4	3.82%	motif file (matrix)	svg
123	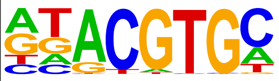	HIF-1b(HLH)/T47D-HIF1b-ChIP-Seq(GSE59937)/Homer	1e-2	-4.762e+00	0.0306	204.0	15.25%	5438.1	12.98%	motif file (matrix)	svg
124	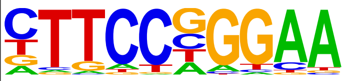	Stat3(Stat)/mES-Stat3-ChIP-Seq(GSE11431)/Homer	1e-2	-4.759e+00	0.0306	76.0	5.68%	1791.3	4.27%	motif file (matrix)	svg
125	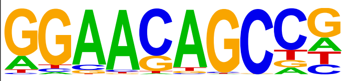	ZNF341(Zf)/EBV-ZNF341-ChIP-Seq(GSE113194)/Homer	1e-2	-4.730e+00	0.0311	117.0	8.74%	2935.2	7.00%	motif file (matrix)	svg
126	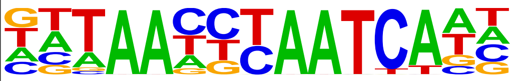	DUX4(Homeobox)/Myoblasts-DUX4.V5-ChIP-Seq(GSE75791)/Homer	1e-2	-4.615e+00	0.0346	5.0	0.37%	41.0	0.10%	motif file (matrix)	svg
