# Supplementary material for: Transcription factor PBX4 regulates limb development and haematopoiesis in mice
Source: Cell Prolif. 2024 Jan 17;57(5):e13580. doi: 10.1111/cpr.13580 (PMC11056705; doi:10.1111/cpr.13580)
Supplement: Supplementary file 9 — Table S5. Sequences of primers and other DNA. [file CPR-57-e13580-s003.docx]

|  | primer | sequence | note |
| --- | --- | --- | --- |
| *Pbx4* cDNA clone | P1s | CCCTGGGTCTGCGGCGGTTG |  |
|  | P1a1 | TGCAAACAGGTCAAAACTTTAATCGGG |  |
|  | P1a2 | GCTCAGAATGAACAGTCCACTGTC |  |
|  | P2s | AAGACTGCCGTGAGCATCCA |  |
|  | P2a | GGATTCTTTTGTTGCCAAACCA |  |
|  | primer | sequence |  |
| Expression | *Pbx4* qF | CTCTTCAGTGTGCTCTGCGA |  |
|  | *Pbx4* qR | GCTTGGCCCTGTAGTCTGAG |  |
|  | *Plzf* qF | CGACTGGGTTGTCACTATGT |  |
|  | *Plzf* qR | GACCACTGGGACCTTTCTC |  |
|  | *Kit* qF | GCCACGTCTCAGCCATCTG |  |
|  | *Kit* qR | GTCGCCAGCTTCAACTATTAACT |  |
|  | *Stra8* qF | ACAAGAGTGAGGCCCAGCAT |  |
|  | *Stra8* qR | CCTCTGGATTTTCTGAGTTGCA |  |
|  | *Sycp3* qF | ATGATGGAAACTCAGCAGCAAGAGA |  |
|  | *Sycp3* qR | TTGACACAATCGTGGAGAGAACAAC |  |
|  | *Prm2* qF | ATGGTTCGCTACCGAATGA |  |
|  | *Prm2* qR | TGATGGTGCCTCCTACATTTC |  |
|  | *WT1* qF | CTGTCGCTACGGACCCTTC |  |
|  | *WT1* qR | TGACCGTGCTGTATCCTTGG |  |
|  | primer | sequence |  |
| KI | *Pbx4* KI gRNA F | taggGGCACCTGGCTGAGTGCCAT |  |
|  | *Pbx4* KI gRNA R | aaacATGGCACTCAGCCAGGTGCC |  |
|  | KI donor | GAATTCcattccagcactgagccaagagtcaggctgtgccatgccctgcaggaacccacctaagtgtcctgtgggtacaacatgtcccaaggacaccctttgtcttgttgtttgtttaagatgatctctctacataccactggctgtcctggtattcactctttagaccaggctagccttgaattcacagagttcttcctgcctctgtctcccaagggcttaaaggaatataccatcatgacagacccatgccaacaccttcatacccattcagctggctcttcttaaaggtcactctgccagcatggccactgttcttctatccagctgcttcagcatatgcctgccatggcctcactcacagagcctctagcacacatgccatgcctgatacagtggacaaggacagtgtcctttgagcttagcacatccatccagctgtccttagacagatgggcagagtagccccaatgtgtgctgtggtcatgcacactgcagacaagcggaggctcctgcccctgtgaggaaggagcccacagtcatggccttgccacaggctgctggttctgacatcccccaaggcatttgctggaggggtgtgggctcagagcccacctggctctttctggaactcctctgctagcatgttctgtgtaggtacctttggggacacaggaactgggtagtcacccactctcatgctcttatctttccagGTCCATAGTAACTGGCAGAGGGCCGCCCCACAGCCAGCCTCATCACCTGCGGGAGAGTCTGGCAGCTTCAATTGGGATGCTGCATCTAATGGAGGAGGAGGATCTGACTACAAAGACCATGACGGTGATTATAAAGATCATGACATCGATTACAAGGACGATGACGACAAGTAAGCCGCGGGTTTCTGGCACCTGGCTGAGTGCCATAGGTGACTGGCCCTGTGTCCTGCTGATGACCTCAGAAGTCCTGGCTGCGGCTTTGCCCTTTTTCAGGCCACTTGCCTCCCCACCTTGGTGTGATTTTTTTTTTTAAAGAAACATCAGTTAAGTGACTGACAGTGGACTGTTCATTCTGAGCACCCGATTAAAGTTTTGACCTGTTTGCAttccagtgttttcaatacaggcttgtctaagtgacaggtgacaacctagatgccattatcagctctaaaaccttgtgggagctgtggcatggcctctgctcttattgggaatgggtttggctttgtggttctgttttagctcttttgtgtttggtttggggattttgagaatttgggagaatttgaagccttatcccatttcccctgtgcagcaaccaatagggccttccctccatagcctgtaccctggagcgcttgtcaggcagtcctcatggctgtgacctcatcctatcagccactatcacaacagtaacaaagtgccccttttcctacagcctgggaagaacccataacaggtggaggccaccttatccctgggacccagccaggtgtaccctgtggctgcctgctatgtgagtgtcaccatgtgctctgctgagcctgagggctgtcaagtggttagcagctacctgtcgtgactggacactctcatcctccaaaggcgctgtctccaccatcagtctgtccccacgcacatctccaggccccgcagccacttatctgggttcaccttcttctgtacagtgtcagtGAATTC | 1700bp |
|  | KI F | TCTGGCAGCTTCAATTGGGAT |  |
|  | KI R | ACCACAAAGCCAAACCCATTC |  |
|  | primer | sequence |  |
| *Pbx4* KO | *Pbx4* KO gRNA F | taggATGTGTGGTGAACTCACGAC |  |
|  | *Pbx4* KO gRNA R | aaacGTCGTGAGTTCACCACACAT |  |
|  | *Pbx4* KO 120F | CACCACTGGCTGCTGAGTCA |  |
|  | *Pbx4* KO 120R | GCAAGACACAGGCCTCACCC |  |
|  | primer | sequence |  |
| *Pbx4* KO-2 | *Pbx4* KO-2 gRNA1F | taggGAAGAGCTGGCCAGGAAGGG |  |
|  | *Pbx4* KO-2 gRNA1R | aaacCCCTTCCTGGCCAGCTCTTC |  |
|  | *Pbx4* KO-2 gRNA2F | taggTGGTCTCTTCACTGGGGTAA |  |
|  | *Pbx4* KO-2 gRNA2R | aaacTTACCCCAGTGAAGAGACCA |  |
|  | *Pbx4* KO-2 189F | TTCCCATTTGAGCAACCCTT |  |
|  | *Pbx4* KO-2 189R | CCCCATACATTAAGTAACTTTCCCA | as155R |
|  | *Pbx4* KO-2 310F | GACTGGGCATCTCTGTCTGTAG |  |
|  | *Pbx4* KO-2 310R | GTCTTCCCTCCTCCCATATTAAC |  |
|  | *Pbx4* KO-2 155F | CAAAGAAGAGCTGGCTTAGAAGG |  |
|  | *Pbx4* KO-2 155R | CCCCATACATTAAGTAACTTTCCCA | as189R |
