# Supplementary material for: Transcription factor PBX4 regulates limb development and haematopoiesis in mice
Source: Cell Prolif. 2024 Jan 17;57(5):e13580. doi: 10.1111/cpr.13580 (PMC11056705; doi:10.1111/cpr.13580)
Supplement: Supplementary file 10 — Table S6. Pbx4 KO mice no off‐target effect. [file CPR-57-e13580-s001.docx]

| sgRNA | Sequence | PAM | Gene | Score | Chromosome | Strand | Position | Misma-tches | predicted On-target | **offtarget dection** |
| --- | --- | --- | --- | --- | --- | --- | --- | --- | --- | --- |
| sgRNA1 | ATGTGTGGTGAACTCACGAC | AGG | **Pbx4** | 100 | chr8 | -1 | 69864831 | **0** | TRUE | **—** |
|  | AAATTTGATGAACTCACGAC | AGG | **Prl2b1** | 1.434 | chr13 | -1 | 27385141 | **4** | FALSE | **No** |
|  | ATGTGTGGTAAGCTCAGGAA | AGG | **Dlgap4** | 0.121 | chr2 | -1 | 156721578 | **4** | FALSE | **No** |
| sgRNA2-1 | GAAGAGCTGGCCAGGAAGGG | TGG | **Pbx4** | 100 | chr8 | 1 | 69866582 | **0** | TRUE | **—** |
|  | AAAGAGCTGCCCAGGAAGGC | CAG | **Spata2** | 1.829 | chr2 | 1 | 167481739 | **3** | FALSE | **No** |
|  | GTAGAGCTGCCCAGGAAGGA | GGG | **Cryl1** | 1.725 | chr14 | 1 | 57275423 | **3** | FALSE | **No** |
|  | GAGGAGCTGACCAGGAAGGT | AGG | **Cchcr1** | 1.610 | chr17 | 1 | 35525087 | **3** | FALSE | **No** |
|  | CCAGAGCAGGCCAGGAAGGC | CAG | **Psmb11** | 0.934 | chr14 | -1 | 54627207 | **4** | FALSE | **No** |
| sgRNA2-2 | TGGTCTCTTCACTGGGGTAA | GGG | **Pbx4** | 100 | chr8 | -1 | 69866546 | **0** | TRUE | **—** |
|  | TGGCTTCCTCACTGGGGTAA | GGG | **Pbx1** | 2.503 | chr1 | 1 | 168195806 | **3** | FALSE | **No** |
|  | TGGCTTCTTCACTGGGGTAG | GGG | **Pbx3** | 1.682 | chr2 | 1 | 34204838 | **3** | FALSE | **No** |
|  | TCTTATCTTCACTGGGGTCA | GAG | **Cxcl5** | 0.618 | chr5 | -1 | 90760535 | **4** | FALSE | **No** |
|  | TGGCCTCCTCACTAGGGTAA | GGG | **Pbx2** | 0.460 | chr17 | -1 | 34594660 | **3** | FALSE | **No** |
